# Discovery of a Series of 1,2,3-Triazole-Containing Erlotinib Derivatives With Potent Anti-Tumor Activities Against Non-Small Cell Lung Cancer

**DOI:** 10.3389/fchem.2021.789030

**Published:** 2022-01-07

**Authors:** Ge Sun, Longfei Mao, Wenjing Deng, Shuxiang Xu, Jie Zhao, Jianxue Yang, Kaitai Yao, Miaomiao Yuan, Wei Li

**Affiliations:** ^1^ Cancer Research Institute, School of Basic Medical Sciences, Southern Medical University, Guangzhou, China; ^2^ Henan Engineering Research Center of Chiral Hydroxyl Pharmaceutical, School of Chemistry and Chemical Engineering, Henan Normal University, Xinxiang, China; ^3^ Department of Neurology, The First Affiliated Hospital of Henan University of Science and Technology, Luoyang, China; ^4^ School of Nursing, Henan University of Science and Technology, Luoyang, China; ^5^ The Eighth Affiliated Hospital, Sun Yat-sen University, Shenzhen, China

**Keywords:** erlotinib, 1,2,3-triazole, NSCLC, EGFR, anti-tumor

## Abstract

Epidermal growth factor receptor (EGFR) tyrosine kinase inhibitors (TKIs) are emerging at the vanguard of therapy for non-small-cell lung cancer (NSCLC) patients with EGFR-activating mutations. However, the increasing therapeutic resistance caused by novel mutations or activated bypass pathways has impaired their performance. In this study, we link one of the commercial EGFR-TKIs, Erlotinib, to different azide compounds to synthesize a novel class of 1,2,3-triazole ring-containing Erlotinib derivatives. We discovered that several new compounds show robust antiproliferation activity against diverse NSCLC cells *in vitro* including PC-9, H460, H1975 and A549. Two of the most potent compounds, e4 and e12 have been found to be more efficient than Erlotinib in all NSCLC cell lines except PC-9. They significantly induce apoptosis and cell cycle arrest in PC-9 and H460 cells. The antitumor efficacy of compound e4 *in vivo* is close to that of Erlotinib in a PC-9 xenograft mouse model. Most Erlotinib-1,2,3-triazole compounds exhibit moderate to good inhibitory activities toward wild-type EGFR as indicated by enzyme-linked immunosorbent assay (ELISA), and the EGFR phosphorylation was inhibited in H460 and PC-9 cells exposed to e4 or e12. These data suggest that these Erlotinib-1,2,3-triazole compounds are suitable candidates for use against NSCLC and more unknown mechanisms merit further investigation.

## Introduction

It is estimated that 2.09 million new cases of lung cancer (11.6% of the total cases) were diagnosed and 1.76 million deaths were caused by lung cancer (18.4% of the total cancer deaths) globally in 2018 (Bray et al., 2018). Non-small-cell lung cancer (NSCLC) accounts for 85% of all the new cases of lung cancer, and is classified as adenocarcinoma (gland-forming), squamous cell carcinoma and large-cell carcinoma according to the histological distinctions (Travis et al., 2013; Gridelli et al., 2015). Lung cancer remains the most common malignant tumor and the leading cause of cancer deaths worldwide. While surgery is recommended for early-stage NSCLC patients, molecular targeted therapy has become the most important treatment for advanced NSCLC patients, up to 69% of which have a potentially actionable molecular target (Hirsch et al., 2017). The development of targeted therapy has significantly improved the survival of NSCLC patients over the past 2 decades.

**FIGURE 1:**
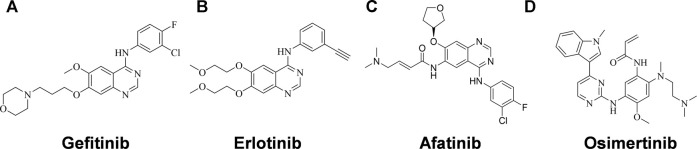
Structures of EGFR-TKIs for NSCLC therapy. **(A)** Gefitinib. **(B)** Erlotinib. **(C)** Afatinib. **(D)** Osimertinib.

Activating mutations in the ATP binding pocket of EGFR and excessive activation of the EGFR signaling pathway are observed in approximately 15–20% of NSCLC patients (Lynch et al., 2004; Paez et al., 2004), and many EGFR tyrosine kinase inhibitors (EGFR-TKIs) have been developed as the first-line therapy for NSCLC, including the first- and second-generation EGFR- TKIs Gefitinib (Figure 1A) (Muhsin et al., 2003), Erlotinib (Figure 1B) (Schettino et al., 2008) and Afatinib (Figure 1C) (Dungo and Keating, 2013), and the third-generation EGFR-TKI Osimertinib (Figure 1D) (Cross et al., 2014). EGFR-TKI inhibits the tyrosine kinase by competing with ATP for the ATP-binding site, thereby suppressing the activation and transduction of downstream signal through RAS-RAF-MEK-ERK and RAS-PI3K-PTEN-AKT-mTOR (Herbst and Bunn, 2003; Kan et al., 2010). Erlotinib is one of the earliest EGFR-TKIs, and it has been shown to be effective in NSCLC patients with or without EGFR-activating mutations, but it is even more potent in patient with EGFR activating mutations (Tsao et al., 2005; Qi et al., 2012; Kobayashi and Hagiwara, 2013). Unfortunately, just like the other first- and second-generation EGFR-TKIs, it has failed large numbers of patients who have eventually develop resistance after administration. More than 60% of the patients develop T790M mutations within the ATP-binding pocket, which prevents drug binding (Sequist et al., 2011). Although Osimertinib is the only inhibitor approved to target the T790M mutation, most third-generation EGFR-TKIs designed to overcome T790M resistance have not shown themselves superior to Osimertinib with respect to antitumor efficacy. It is necessary to identify more competent agents against NSCLC that could work quickly and effectively.

In recent years, many researchers focused on synthesizing more potent and safer quinazoline-derived EGFR inhibitors with the aim of overcoming limitations of currently marketed drugs (Bhatia et al., 2020). For example, Elkamhawy and others (Elkamhawy et al., 2015a) designed a novel series of eighteen 6-anilinoquinazolines as inhibitors of EGFR/HER-2. The authors concluded that “11” was a promising candidate through studying the structural activity relationship of the compounds along with their molecular docking studies. In addition, they also reported new quinazoline-urea based compounds with potent cytotoxic activities against TMZ-resistant glioblastoma multiforme (GBM) cells, which may be associated with plausible binding modes in translocator protein 18 kDa (TSPO). Overall, these studies proved that novel quinazoline-based compounds are promising candidates for cancer therapy possessing potency and various activity (Elkamhawy et al., 2015b).

1,2,3-triazoles are prevailing motifs that exist in different compounds with particular bioactivities, such as antitumor, antimicrobial, and antidiabetic effects (Maddili et al., 2018; Safavi et al., 2018; Saeedi et al., 2019). In this study, we aimed to design and synthesize a novel class of 1,2,3-triazole ring-containing Erlotinib derivatives with EGFR tyrosine kinase inhibitory activities and improved antitumor activities against NSCLC.

## Results

### Chemistry

The strategy for the preparation of the target compounds is illustrated in Scheme 1. Erlotinib was obtained after reaction of 4-chloro-6,7-bis (methoxy ethoxy) quinazolinone and 3-aminophenylacetylene. And the target compounds e1–e20 were obtained via click reaction of Erlotinib with different azido compounds. The reaction conditions were mild and easy to control. The structures of the key intermediates and all target compounds were confirmed by nuclear magnetic resonance (^1^H NMR and ^13^C NMR) and high-resolution mass spectrometry (HRMS) (Supplementary Figures S1–20).

**SCHEME 1:**
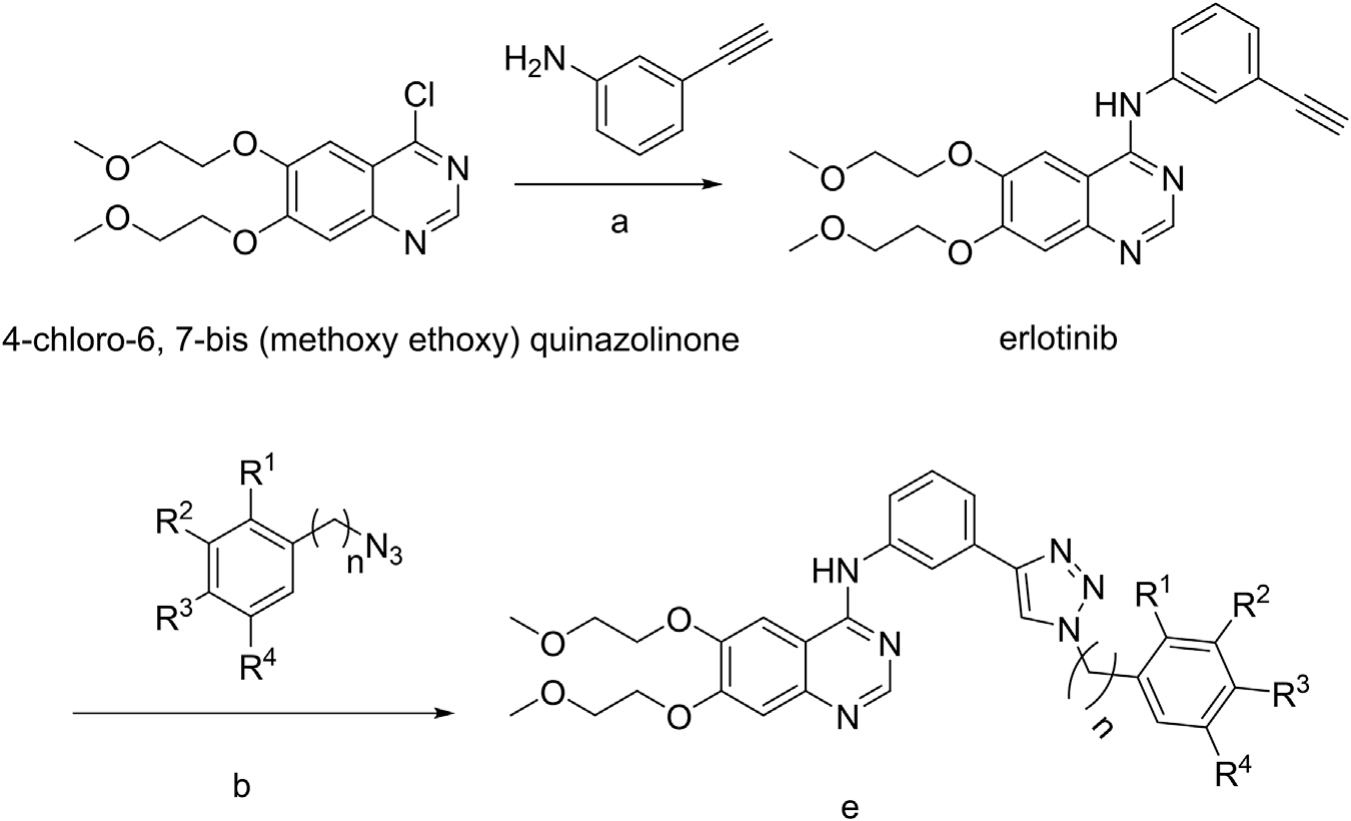
a, 85°C, isopropanol; b, copper sulfate pentahydrate, sodium ascorbate; e, Erlotinib derivatives.

### *In Vitro* Antitumor Activity Study

To probe the therapeutic potential of all the synthesized derivatives, CCK-8 (Cell Counting Kit-8) assay was performed to evaluate the *in vitro* antiproliferative activities of all the new compounds against four human NSCLC cell lines: H460 and A549 express wild type EGFR (Helfrich et al., 2006); PC-9 is sensitive to Erlotinib and characterized by expressing EGFR exon 19 deletion mutation (ΔE746-A750) (Chmielecki et al., 2011); H1975 harbored the EGFR-activating mutations L858R but also the T790M mutation, which is associated with Erlotinib resistance (Helfrich et al., 2006).

#### Antiproliferation Assay

The antiproliferative effects of the new compounds and Erlotinib were evaluated by CCK-8 assay. As the data shown in Table 1, PC-9 cells were still most sensitive to Erlotinib with an IC_50_ value of 0.21 μΜ. However, compared to Erlotinib, some compounds including e4, e6, e9, e12 and e16 presented lower but also exceptional inhibitory efficacy against PC-9 with IC_50_ values of 4.38, 3.27, 7.63, 6.36 and 7.71 μΜ. Besides, for the other three kinds of NSCLC cells, e4, e9, and e12 showed more potent antiproliferative activities than Erlotinib with IC_50_ values less than 10 μΜ.

**TABLE 1:** The antitumor activities of the designed compounds against NSCLC cells *in vitro*.

Compd no.	n	R^1^	R^2^	R^3^	R^4^	IC_50_ (μM)			
						H460	H1975	A549	PC-9
e1	1	H	H	H	H	>100	>100	>100	>100
e2	1	I	H	H	H	9.08 ± 2.81	15.71 ± 2.76	11.79 ± 1.70	11.62 ± 3.64
e3	1	Br	H	H	H	>100	16.78 ± 1.02	>100	>100
e4	1	H	Br	H	Br	5.18 ± 0.78	3.31 ± 0.47	5.80 ± 0.78	4.38 ± 0.12
e5	1	H	OCH_3_	H	H	>100	27.49 ± 7.58	9.97 ± 1.57	>100
e6	0	F	H	H	H	18.49 ± 2.00	16.52 ± 3.53	4.88 ± 0.54	3.27 ± 0.14
e7	0	H	H	F	H	>100	13.05 ± 4.41	>100	11.54 ± 1.57
e8	0	Cl	H	H	H	4.18 ± 0.49	7.19 ± 0.68	23.01 ± 0.75	>100
e9	0	Br	H	H	H	2.72 ± 0.89	6.86 ± 0.17	2.53 ± 0.09	7.63 ± 1.69
e10	0	H	H	Br	H	15.03 ± 2.10	6.36 ± 4.21	3.77 ± 0.68	>100
e11	0	OCH_3_	H	H	H	4.89 ± 0.23	10.81 ± 1.57	8.40 ± 0.29	16.02 ± 1.04
e12	0	H	H	CH_3_	H	3.77 ± 0.60	7.25 ± 0.45	5.91 ± 0.73	6.36 ± 0.11
e13	0	H	NO_2_	H	H	11.86 ± 3.63	>100	5.95 ± 1.47	22.56 ± 1.16
e14	0	H	OCH_2_CH_3_	H	H	34.08 ± 2.50	7.50 ± 0.49	>100	>100
e15	0	H	H	H	H	>100	>100	>100	>100
e16	0	CF_3_	H	H	H	10.93 ± 0.39	15.29 ± 1.55	5.75 ± 0.48	7.71 ± 1.15
e17	0	OCH_3_	H	OCH_3_	H	3.18 ± 0.11	18.30 ± 4.45	4.86 ± 0.60	>100
e18	0	OH	H	CH_3_	H	4.30 ± 0.62	10.02 ± 0.69	2.53 ± 0.20	15.24 ± 0.75
e19	0	H	OCH_3_	OCH_3_	H	>100	>100	>100	23.46 ± 10.25
e20	2	H	H	H	H	>100	>100	>100	>100
Erlotinib						13.28 ± 1.50	12.67 ± 2.76	10.20 ± 0.32	0.21 ± 0.07

IC_50_ values were obtained from three independent experiments. These results are reported as average ± SD.

#### Cell Apoptosis Assay

**FIGURE 2:**
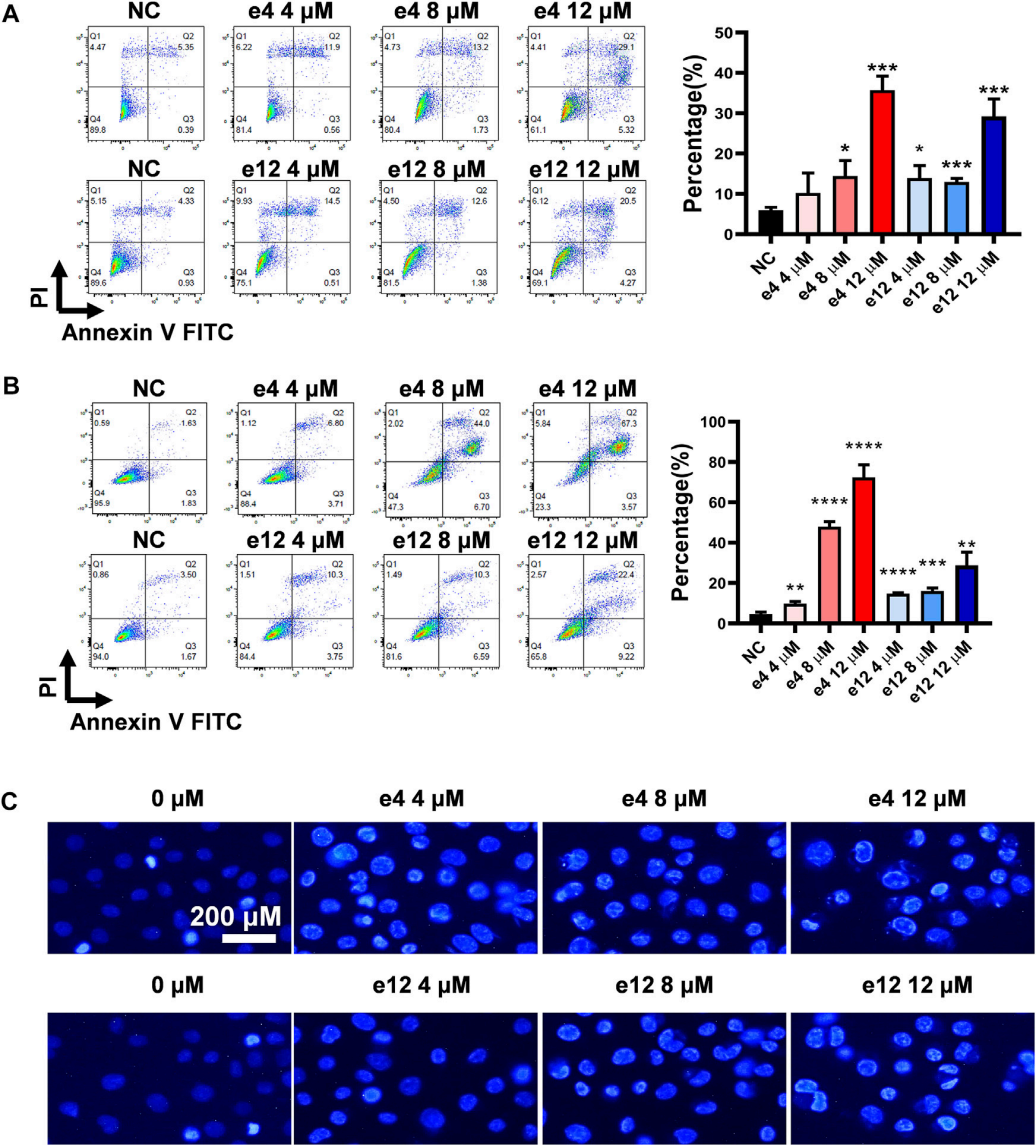
Compounds e4 and e12 induced apoptosis of PC-9 and H460 cells. PC-9 **(A)** and H460 **(B)** cells were exposed to compound e4 or e12 for 48 h. Flow cytometry analysis data from three independent experiments were summarized and shown. NC, negative control. *p-value < 0.05, **p-value < 0.01 and ***p-value < 0.001 (unpaired *t*-test). **(C)** Representative images of Hoechst 33342 staining of PC-9 cells exposed to compound e4 or e12 for 48 h. Images were analyzed with a fluorescence microscope (×200, original magnification).

To establish whether the antiproliferative efficacy is associated with apoptosis, PC-9 and H460 cells were treated with compound e4 or e12 of various concentrations (4, 8 and 12 μM) for 48 h, and then assessed with flow cytometry. As shown in Figures 2A,B, we determined significant cell apoptosis in both PC-9 and H460 cells exposed to various concentrations of e4 or e12.

To further verify the impact of compounds e4 and e12 on apoptosis of NSCLC cells, PC-9 cells were treated with either compound e4 or e12 at various concentrations for 48 h and then stained with dye Hoechst 33342. We compared PC-9 cells to cells treated with 0.1% DMSO, and they exhibited features typical of apoptosis such as condensed cell nuclei, fragmented chromatin and cell shrinkage (Brady, 2004) (Figure 2C). Taken together, these results indicate that compounds e4 and e12 induced cell apoptosis, leading to exceptional antiproliferative activities against NSCLC cells.

#### Cell Cycle Assay

**FIGURE 3:**
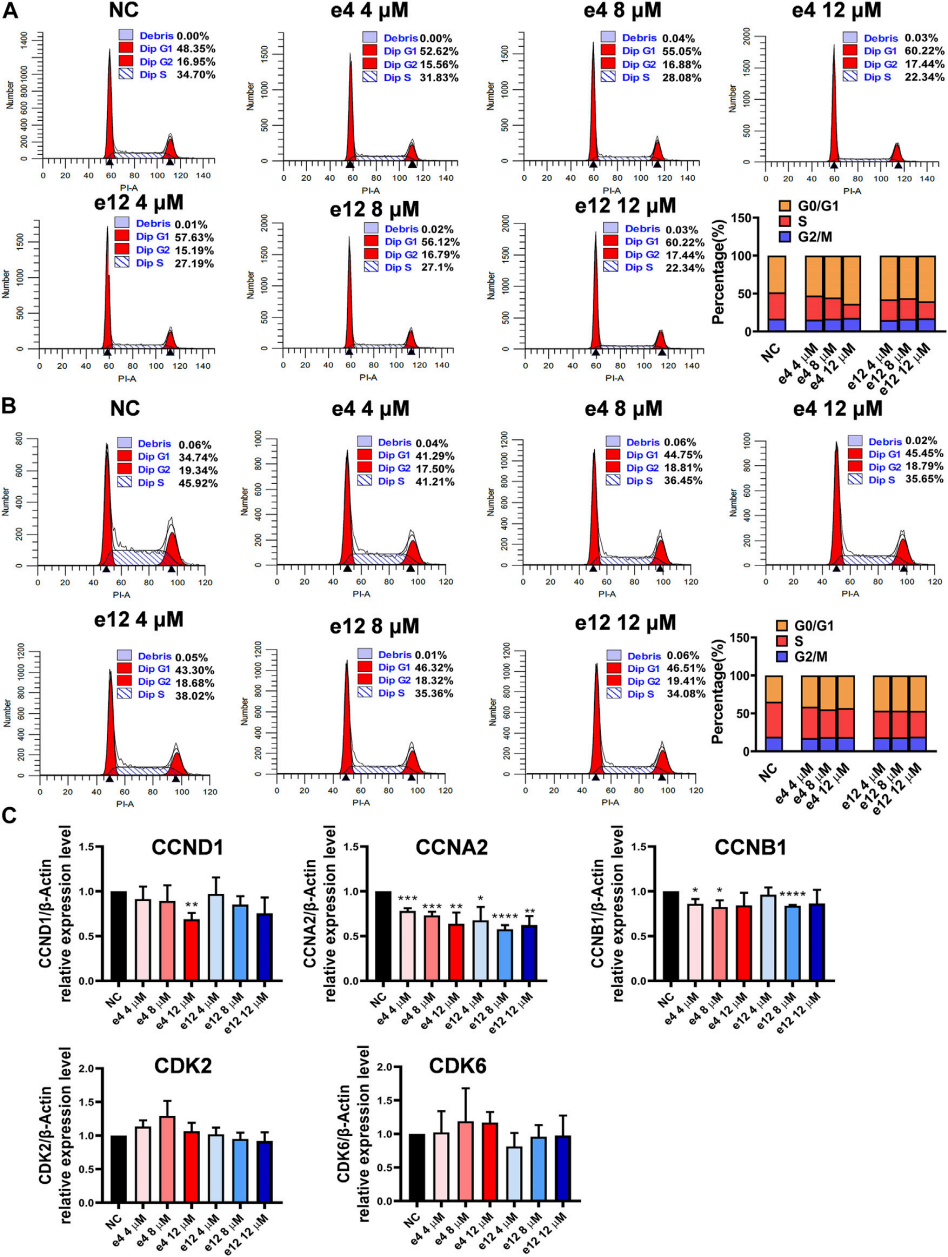
Cell cycle arrests induced by compounds e4 and e12 in PC-9 and H460 cells. PC-9 **(A)** and H460 **(B)** cells were exposed to either compound e4 or e12 at various concentrations respectively. The cell cycle and the proportions of each phase were detected through flow cytometry. This experiment was repeated three times independently with similar results. **(C)** Cell cycle-related gene analysis by qPCR in PC-9. PC-9 cells were treated with compound e4 or e12 at various concentrations for 16 h. Genes including CCND1, CCNA2, CCNB1, CDK2 and CDK6 were quantified. The results are reported as a mean ± SD of values from three independent experiments (*p < 0.05, **p < 0.01, ***p < 0.001, ****p < 0.0001 vs. Ctrl). NC, negative control.

To investigate the effects of compounds e4 and e12 on various phases of the cell cycle, PC-9 cells were treated with compound e4 or e12 of various concentrations for 24 h, and H460 cells were treated with compound e4 or e12 of various concentrations for 12 h. As shown in Figures 3A,B, the results of flow cytometry indicated that compounds e4 and e12 induced higher percentages of PC-9 (Figure 3A) and H460 (Figure 3B) cells in the G0/G1 phrase during incubation.

To better understand the mechanisms underlying cell cycle arrest induced by e4 and e12 in NSCLC cells, we assessed the expression levels of a few genes involved in cell cycle regulation, including cyclin D1 (CCND1), cyclin A2 (CCNA2), cyclin B1 (CCNB1), cyclin-dependent kinase 2 (CDK2), and cyclin-dependent kinase 6 (CDK6) in PC-9 by qPCR. As shown in Figure 3C, compared with DMSO control, e4 and e12 significantly decreased RNA levels of CCNA2 and CCNB1 in PC-9 cells over 16 h of incubation. In addition, e4 also negatively regulated the RNA expression of CCND1 at the concentration of 12 μM. CCNA2 is known to bind and activate CDK2 and thus promotes the transition through G1/S and G2/M. CCNB1 and CCND1 are essential to mitosis and G1/S transition respectively. While G2/M arrest weas not detected, the changes in these gene expression profiles interpret the G1/S arrests in PC-9 cells treated with e4 and e12. PC-9 cells exposed to the compounds for various periods should be examined further. In conclusion, e4 and e12 impeded cell proliferation by arresting the cell cycle at the G0/G1 phases in PC-9 and H460 cells, which was further confirmed by the decreased expression of genes involved in regulation of the cell cycle in PC-9 cells.

### *In Vivo* Antitumor Activity

**FIGURE 4:**
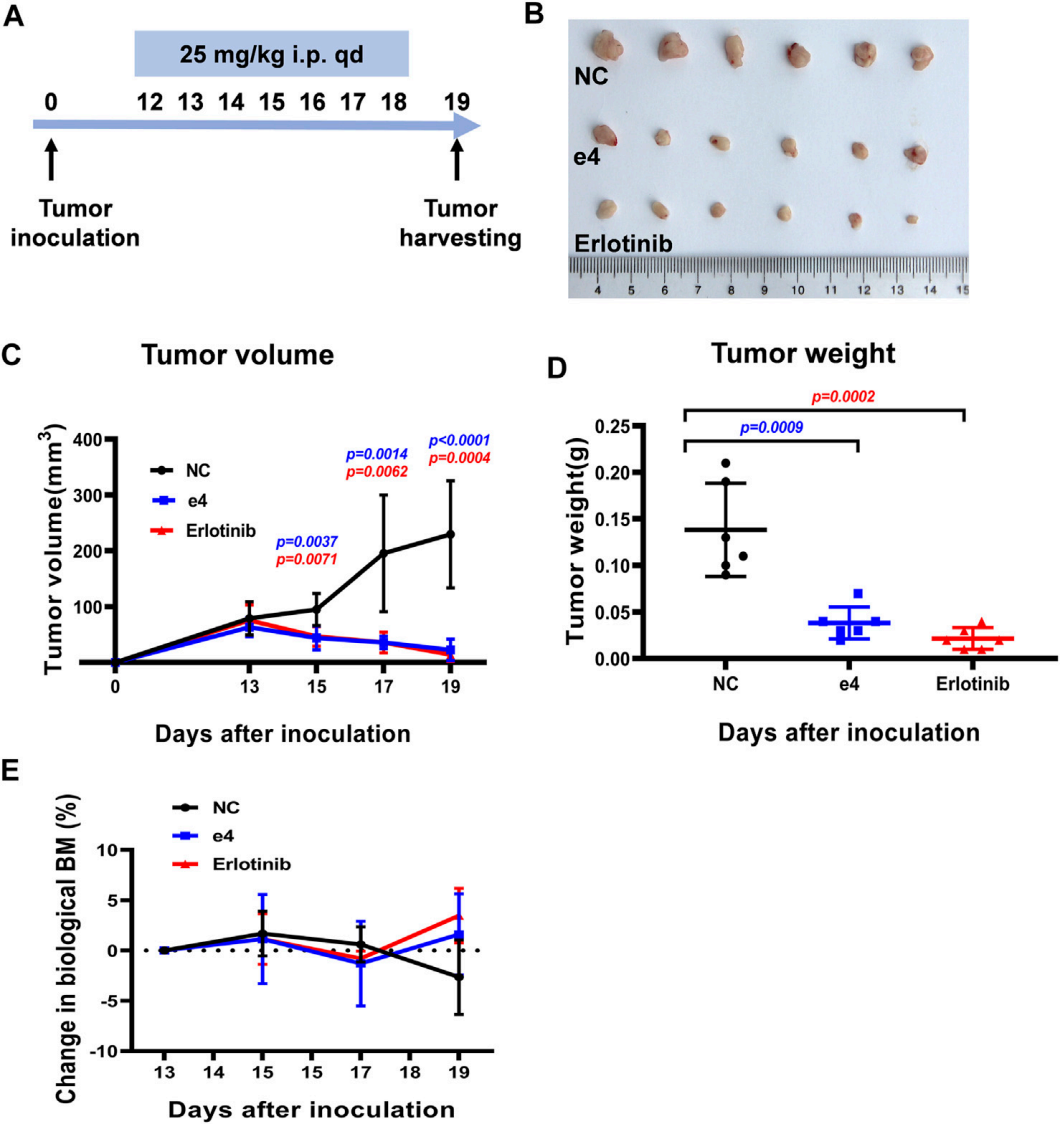
Compound e4 inhibits tumor growth of PC-9 xenograft in NSG mice. **(A)** Schematic diagram of the experimental procedure. **(B)** The images of the tumors formed by PC-9 cells treated with vehicle, compound e4 or Erlotinib 19 days after inoculation (*n* = 6). **(C)** Growth curves for the PC-9 tumors from the NSG mice after treatment with vehicle, compound e4 or Erlotinib (*n* = 6, 9 and 7 respectively). **(D)** The tumor weight of PC-9 xenograft and the corresponding statistical results were presented (*n* = 6). **(E)** The changes in the body mass of the mice after treatment. NC, negative control. Statistical significance was evaluated by the Student’s *t*-test.

Inspired by the pronounced EGFR activities and the antitumor activities of these derivatives *in vitro*, we next determine the *in vivo* antitumor efficacy using mouse tumor models. NSG mice harboring PC-9 tumors were dosed with compound e4 (25 mg/kg, qd) and Erlotinib (25 mg/kg, qd) by intraperitoneal injection for 7 consecutive days (Figure 4A). As shown in Figure 4C, treatment with e4 or Erlotinib exhibited similar potency for tumor growth inhibition over treatment with vehicle. There was no significant difference with respect to growth rate between groups treated with e4 and Erlotinib (Figures 4B,D). No significant difference was detected in the changes of body mass between the three groups after treatment (Figure 4E), indicating that compound e4 had no obvious toxicity *in vivo*.

### EGFR Inhibition Study

Aberrant EGFR signaling in NSCLC promotes cell proliferation and survival, and Erlotinib inhibits the EGFR tyrosine kinase and its transduction of downstream signaling in NSCLC cells. To clarify whether the antiproliferative efficacy of the new compounds was associated with EGFR inhibitory activities, all the new compounds were assayed for their activities to inhibit EGFR tyrosine phosphorylation *in vitro* using ELISA. Erlotinib was served as a positive control. The results were shown as IC_50_ values in Table 2. Under these conditions, the IC_50_ of Erlotinib was 4.8 nM, which was similar to previously reported values (Moyer et al., 1997; Akita and Sliwkowski, 2003) (IC_50_ = 2 nM). As illustrated in Table 1, while all the Erlotinib derivatives showed less ability to inhibit EGFR tyrosine phosphorylation (IC_50_ values ranged from 0.09 to 63.02 μM), e2, e6, e15 and e20 displayed favorable EGFR inhibitory activities (IC_50_ values < 1 μM), especially compound e15, whose IC_50_ value was 0.09 μM.

**TABLE 2:** EGFR inhibitory activities of the derivatives.

Compd no.	n	R^1^	R^2^	R^3^	R^4^	IC_50_ (μM) EGFR
e1	1	H	H	H	H	1.34
e2	1	I	H	H	H	0.32
e3	1	Br	H	H	H	68.02
e4	1	H	Br	H	Br	13.01
e5	1	H	OCH_3_	H	H	35.16
e6	0	F	H	H	H	0.69
e7	0	H	H	F	H	14.74
e8	0	Cl	H	H	H	1.99
e9	0	Br	H	H	H	3.83
e10	0	H	H	Br	H	2.13
e11	0	OCH_3_	H	H	H	49.39
e12	0	H	H	CH_3_	H	1.76
e13	0	H	NO_2_	H	H	4.74
e14	0	H	OCH_2_CH_3_	H	H	10.66
e 15	0	H	H	H	H	0.09
e16	0	CF_3_	H	H	H	32.53
e17	0	OCH_3_	H	OCH_3_	H	15.49
e18	0	OH	H	CH_3_	H	12.38
e19	0	H	OCH_3_	OCH_3_	H	28.08
e20	2	H	H	H	H	0.28
Erlotinib						0.0048

Kinase inhibitory activities of the compounds were evaluated using the Enzyme-linked immunosorbent assay (ELISA).

**FIGURE 5:**
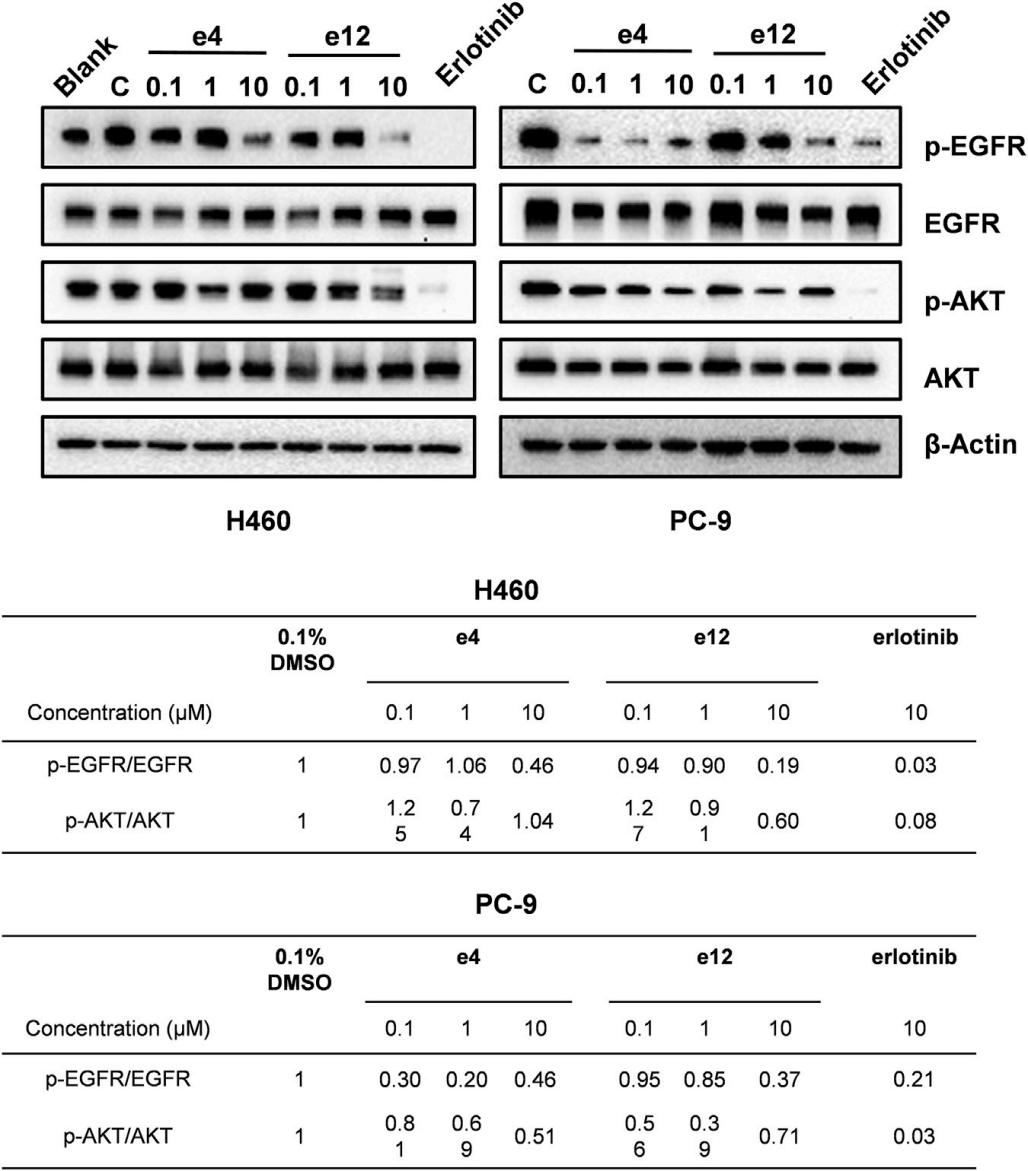
Compounds e4 and e12 suppressed EGFR phosphorylation and its transduction of downstream signaling in NSCLC cells. Protein bands (images on the top) and quantification (tables at the bottom) are presented.

We also performed western blot analysis to evaluate the impact of e4 and e12 on EGFR signaling in PC-9 and H460 cells. The results showed that e4 and e12 suppressed the phosphorylation of EGFR and AKT in both PC-9 and H460 cells, especially at a concentration of 10 μM, consistent with their potent antiproliferation activities in NSCLC cells (Figure 5). In addition, we conducted molecular docking studies to explore the potential binding modes of compound e4 in the active site of EGFR. It showed that e4 has a desirable binding affinity with EGFR but a different binding mode from that of Erlotinib (Supplementary Figure S21).

## Discussion

The survival rate of NSCLC patients has improved considerably since the adoption of EGFR-TKIs. Nevertheless, the constant emergence of novel mutations after treatment finally lead to their ineffectiveness. In this study, a new class of Erlotinib derivatives containing 1,2,3-triazole ring were designed and synthesized. Cell-based assay discovered that PC-9 cells that harbored an EGFR-activating mutation, is still most sensitive to Erlotinib. However, several of the Erlotinib derivatives (e.g., compounds e4, e9 and e12) showed more robust inhibitory activity against NSCLC cells including H460, H1975 and A549, compared with Erlotinib. We confirmed that these Erlotinib derivatives hah slightly lower but still adequate EGFR tyrosine kinase inhibitory activity when compared with that of Erlotinib. We suspect that the antiproliferative activities of the new compounds against NSCLC cells probably incompletely depend on the EGFR inhibitory effects, H460 and A549 cells do not express EGFR-activating mutations, and H1975 cells, which are resistant to Erlotinib, whereas are sensitive to some Erlotinib derivatives. This indicates that they may act via such mechanisms as the cancerous inhibitor of protein phosphatase 2A (CIP2A) pathway, through which Erlotinib induces apoptosis in NSCLC cells that do not have EGFR mutations; however, this should be investigated further (Wang et al., 2014). In addition, we confirmed that compound e4 restrains growth of EGFR-activating-mutant PC-9 xenografts and its antitumor efficacy *in vivo* is close to that of Erlotinib. As all considered above, we conclude that these Erlotinib-1,2,3-triazole compounds with EGFR tyrosine kinase inhibitory activities may serve as novel antitumor agents against NSCLC and additional mechanisms merit further investigation.

## Materials and Methods

### Materials

The 4-chloro-6,7-bis (methoxy ethoxy) quinazolinone, 3-aminophenylacetylene and Azido compounds were purchased from Acros Organics (United States). The Dulbecco’s Modified Eagle medium (DMEM), RPMI-1640 medium, phosphate buffered saline (PBS), fetal bovine serum (FBS) and trypsin were purchased from Gibco (United States). Cell Counting Kit-8 (CCK-8) was purchased from Dojindo Molecular Technologies Inc. (Japan). Annexin V/Propidium iodide (PI) staining kit was purchased from BD Biosciences (United States). Hoechst 33342 and RIPA lysis buffer was purchased from Beyotime Biotechnology CO., LTD. (China). Cell-cycle staining kit was purchased from MULTISCIENCES (LIANKE) BIOTECH, CO., LTD. (China). RNA-Quick Purification Kit was purchased from Shanghai Yishan Biotechnology CO., LTD. (China). High Capacity cDNA Reverse Transcription Kit was purchased from Applied Biosystems (United States). TB Green™ Premix Ex Taq™ II (Tli RNaseH Plus) was purchased from Takara Bio Inc. (Japan). The primary antibodies of EGFR, p-EGFR (Tyr1068), Akt, AKT1 (phospho S473) and the secondary antibodies of anti-rabbit and anti-mouse were purchased from Cell Signaling Technology, Inc. (United States). NSCLC cell lines PC-9, H460, A549 and H1975 were purchased from ATCC.

### Chemistry

The general procedures of preparation for Erlotinib and compounds e1–e20 were described in the section of results. The structures of the all target compounds including Erlotinib were confirmed by nuclear magnetic resonance (^1^H NMR and ^13^C NMR) and high-resolution mass spectrometry (HRMS) as below.

The 4-chloro-6, 7-bis (methoxy ethoxy) quinazolinone (3.1 g, 10 mmol) was suspended in 50 ml of isopropanol and 3-aminophenylacetylene (1.3 g, 11 mmol) was added. The suspension was stirred at room temperature for 5 min and then stirred at 85°C under nitrogen overnight. The orange solution was concentrated to obtain an orange solid residue which was recrystallized from EtOAc to yield Erlotinib as an off white solid (3.2 g). ^1^H NMR (600 MHz, DMSO-d_6_): δ 9.48 (s, 1H), 8.51 (s, 1H), 8.00 (s, 1H), 7.91 (d, J = 9.5 Hz, 1H), 7.87 (s, 1H), 7.41 (t, J = 7.9 Hz, 1H), 7.27–7.17 (m, 2H), 4.30 (d, J = 15.1 Hz, 4H), 4.21 (s, 1H), 3.78 (d, J = 31.1 Hz, 4H), 3.38 (s, 3H), 3.36 (s, 3H);^13^C NMR (150 Hz, DMSO-d_6_): 156.59, 154.15, 153.27, 148.61, 147.49, 140.28, 129.37, 126.81, 125.21, 123.02, 122.21, 109.39, 108.69, 103.65, 83.97, 81.03, 70.59, 70.52, 68.85, 68.52, 58.87, 58.82; HR MS(ESI)m/z: calcd for C_22_H_23_O_4_N_3_Na [M+Na]^+^ 416.1581, found 416.1585.

Aryl-azido (1.2 mmol) and Erlotinib (1.0 mmol) were added to 20 ml mixed solvent (water/tert-butanol/tetrahydrofuran = 1:1:1). The reaction was catalyzed with copper sulfate pentahydrate (0.1 mmol) and sodium ascorbate (0.2 mmol), and the mixture then stirred at 60°Cfor 5 h. When the reaction ended (monitored by TLC), the mixture was extracted with dichloromethane (20 ml × 3). The combined organic phase was washed successively with water and brine, dried over sodium sulfate and concentrated in vacuo. Finally, the residue was purified through column chromatography (CH_2_Cl_2_/MeOH) to obtain the desired compounds e1–e20 as crystalline powder.

[3-(1-Benzyl-1H-[1,2,3]triazol-4-yl)-phenyl]-[6,7-bis-(2-methoxy-ethoxy)-quinazolin-4-yl]-amine (e1). Purity 99.1%; m.p.89–92°C; ^1^H NMR (600 MHz, DMSO-d_6_): δ 9.56 (s, 1H), 8.67 (s, 1H), 8.49 (s, 1H), 8.27 (s, 1H), 7.95–7.86 (m, 2H), 7.56 (d, J = 7.7 Hz, 1H), 7.51–7.28 (m, 6H), 7.24 (s, 1H), 5.67 (s, 2H), 4.31 (d, J = 21.7 Hz, 4H), 3.78 (d, J = 32.4 Hz, 4H), 3.39 (s, 3H), 3.36 (s, 3H). ^13^C NMR (150 Hz, DMSO-d_6_): 156.83, 154.06, 153.40, 148.56, 147.44, 147.12, 140.52, 136.50, 131.39, 129.48, 129.30, 128.66, 128.42, 122.28, 122.12, 120.79, 119.23, 109.43, 108.68, 103.69, 70.60, 70.54, 68.83, 68.51, 58.88, 58.82, 53.53; HR MS (ESI) m/z: calcd for C_29_H_30_O_4_N_6_Na [M+Na]^+^ 549.2221, found 549.2231.

[6,7-Bis-(2-methoxy-ethoxy)-quinazolin-4-yl]-{3-[1-(2-iodo-benzyl)-1H-[1,2,3]triazol-4-yl]-phenyl}-amine (e2). Purity 98.4%; m.p.93–96°C; ^1^H NMR (600 MHz, DMSO-d_6_): δ 9.63 (s, 1H), 8.64 (s, 1H), 8.54 (s, 1H), 8.32 (s, 1H), 8.06–7.90 (m, 3H), 7.63 (d, J = 7.7 Hz, 1H), 7.50 (dd, J = 16.4, 8.0 Hz, 2H), 7.28 (s, 1H), 7.20 (dd, J = 11.8, 7.6 Hz, 2H), 5.75 (s, 2H), 4.35 (d, J = 20.8 Hz, 4H), 3.83 (d, J = 31.5 Hz, 4H), 3.43 (s, 3H), 3.41 (s, 3H). ^13^C NMR (150 Hz, DMSO-d_6_): 156.86, 154.09, 153.34, 148.57, 147.30, 146.93, 140.50, 140.00, 138.36, 131.32, 130.80, 130.17, 129.49, 129.38, 122.52, 122.38, 120.88, 119.30, 109.42, 108.58, 103.72, 99.70, 70.59, 70.54, 68.84, 68.52, 58.88, 58.83, 58.03; HR MS(ESI)m/z: calcd for C_29_H_29_O_4_N_6_INa [M+Na]^+^ 675.1187, found 675.1196.

[6,7-Bis-(2-methoxy-ethoxy)-quinazolin-4-yl]-{3-[1-(2-bromo-benzyl)-1H-[1,2,3]triazol-4-yl]-phenyl}-amine (e3). Purity 99.3%; m.p.94–97°C; ^1^H NMR (600 MHz, DMSO-d_6_): δ 9.60 (s, 1H), 8.63 (s, 1H), 8.50 (s, 1H), 8.27 (s, 1H), 7.98–7.84 (m, 2H), 7.72 (d, J = 7.9 Hz, 1H), 7.58 (d, J = 7.7 Hz, 1H), 7.46 (dt, J = 11.5, 7.7 Hz, 2H), 7.34 (t, J = 8.3 Hz, 1H), 7.29–7.16 (m, 2H), 5.76 (s, 2H), 4.31 (d, J = 20.5 Hz, 4H), 3.78 (d, J = 31.6 Hz, 4H), 3.38 (s, 3H), 3.36 (s, 3H). ^13^C NMR (150 Hz, DMSO-d_6_): 156.88, 154.10, 153.31, 148.58, 147.22, 146.92, 140.47, 135.28, 133.42, 131.32, 131.00, 129.94, 129.50, 128.83, 123.37, 122.53, 122.41, 120.91, 119.33, 109.41, 108.53, 103.72, 87.36, 70.60, 70.53, 68.84, 68.52, 58.88, 58.83, 53.63; HR MS(ESI)m/z: calcd for C_29_H_29_O_4_N_6_BrNa [M+Na]^+^ 627.1331, found 627.1336.

[6,7-Bis-(2-methoxy-ethoxy)-quinazolin-4-yl]-{3-[1-(3,5-dibromo-benzyl)-1H-[1,2,3]triazol-4-yl]-phenyl}-amine (e4). Purity 98.9%; m.p.102–105°C; ^1^H NMR (600 MHz, DMSO-d_6_): δ 9.58 (s, 1H), 8.72 (s, 1H), 8.49 (s, 1H), 8.28 (s, 1H), 7.99–7.89 (m, 2H), 7.86 (s, 1H), 7.64 (s, 2H), 7.57 (d, J = 7.6 Hz, 1H), 7.47 (t, J = 7.9 Hz, 1H), 7.24 (s, 1H), 5.70 (s, 2H), 4.35–4.26 (m, 4H), 3.83–3.73 (m, 4H), 3.39 (s, 3H), 3.36 (s, 3H); ^13^C NMR (150 Hz, DMSO-d_6_): 156.84, 154.06, 153.39, 148.56, 147.45, 147.22, 140.83, 140.56, 133.73, 131.21, 130.67, 129.51, 123.23, 122.41, 120.83, 119.30, 109.44, 108.68, 103.71, 70.61, 70.54, 68.84, 68.51, 58.88, 58.82, 52.01; HR MS(ESI)m/z: calcd for C_29_H_29_O_4_N_6_Br_2_Na [M+Na]^+^683.0612, found 683.0624.

[6,7-Bis-(2-methoxy-ethoxy)-quinazolin-4-yl]-{3-[1-(3-methoxy-phenyl)-1H-[1,2,3]triazol-4-yl]-phenyl}-amine (e5). Purity 99.5%; m.p.85–88°C; ^1^H NMR (600 MHz, DMSO-d_6_): δ 9.61 (s, 1H), 8.66 (s, 1H), 8.27 (s, 1H), 8.01 (s, 1H), 7.91 (d, J = 7.9 Hz, 1H), 7.57 (d, J = 7.6 Hz, 1H), 7.46 (t, J = 7.9 Hz, 1H), 7.32 (t, J = 7.9 Hz, 2H), 6.97 (s, 1H), 6.93 (d, J = 7.9 Hz, 2H), 5.63 (s, 2H), 4.32 (d, J = 9.1 Hz, 4H), 3.80 (d, J = 9.0 Hz, 4H), 3.76 (s, 3H), 3.39 (s, 3H), 3.37 (s, 3H).^13^C NMR (150 Hz, DMSO-d_6_): 159.96, 156.75, 154.04, 148.63, 147.09, 140.46, 137.91, 131.41, 130.47, 129.50, 122.35, 122.13, 120.87, 120.51, 119.31, 114.27, 113.98, 108.94, 103.82, 87.75, 70.60, 70.54, 68.85, 68.54, 58.88, 58.83, 55.61, 53.46, 22.56; HR MS(ESI)m/z: calcd for C_30_H_33_O_5_N_6_ [M+H]^+^ 557.2512, found 557.2508.

[6,7-Bis-(2-methoxy-ethoxy)-quinazolin-4-yl]-{3-[1-(2-fluoro-phenyl)-1H-[1,2,3]triazol-4-yl]-phenyl}-amine (e6). Purity 98.6%; m.p.83–86°C; ^1^H NMR (600 MHz, DMSO-d_6_): δ 9.62 (s, 1H), 9.11 (s, 1H), 8.51 (s, 1H), 8.39 (s, 1H), 8.04–7.86 (m, 3H), 7.73–7.59 (m, 3H), 7.56–7.46 (m, 2H), 7.25 (s, 1H), 4.37–4.28 (m, 4H), 3.83–3.74 (m, 4H), 3.39 (s, 3H), 3.37 (s, 3H). ^13^C NMR (150 Hz, DMSO-d_6_): 156.85, 155.22, 154.08, 153.41, 148.57, 147.40, 140.64, 131.88, 130.76, 129.61, 126.56, 126.08, 123.40, 122.74, 121.08, 119.48, 117.75, 117.62, 109.46, 108.69, 103.71, 70.61, 70.54, 68.85, 68.51, 58.88, 58.82; HR MS(ESI)m/z: calcd for C_28_H_27_O_4_N_6_FNa [M+Na]^+^ 553.1970, found 553.1979.

[6,7-Bis-(2-methoxy-ethoxy)-quinazolin-4-yl]-{3-[1-(4-fluoro-phenyl)-1H-[1,2,3]triazol-4-yl]-phenyl}-amine (e7). Purity 97.9%; m.p.88–91°C; ^1^H NMR (600 MHz, DMSO-d_6_): δ 9.73 (s, 1H), 9.32 (s, 1H), 8.57 (s, 1H), 8.38 (s, 1H), 8.14–7.86 (m, 4H), 7.67 (d, J = 7.6 Hz, 1H), 7.52 (dt, J = 12.5, 8.3 Hz, 3H), 7.26 (s, 1H), 4.41–4.25 (m, 4H), 3.85–3.72 (m, 4H), 3.39 (s, 3H), 3.37 (s, 3H); ^13^C NMR (150 Hz, DMSO-d_6_): 162.98, 161.35, 156.98, 156.96, 154.21, 153.11, 148.67, 147.80, 140.47, 133.71, 130.95, 129.63, 122.88, 122.82, 120.45, 119.61, 117.36, 117.20, 108.32, 103.79, 70.59, 70.53, 68.87, 68.57, 58.89, 58.83; HR MS(ESI)m/z: calcd for C_28_H_27_O_4_N_6_FNa [M+Na]^+^ 553.1970, found 553.1979.

[6,7-Bis-(2-methoxy-ethoxy)-quinazolin-4-yl]-{3-[1-(2-chloro-phenyl)-1H-[1,2,3]triazol-4-yl]-phenyl}-amine (e8). Purity 99.0%; m.p.131–134°C; ^1^H NMR (600 MHz, DMSO-d_6_): δ 9.62 (s, 1H), 9.08 (s, 1H), 8.51 (s, 1H), 8.40 (s, 1H), 7.94 (d, J = 10.6 Hz, 2H), 7.85–7.79 (m, 2H), 7.70–7.61 (m, 3H), 7.52 (t, J = 7.9 Hz, 1H), 7.25 (s, 1H), 4.36–4.27 (m, 4H), 3.84–3.74 (m, 4H), 3.39 (s, 3H), 3.37 (s, 3H); ^13^C NMR (150 Hz, DMSO-d_6_): 156.86, 154.09, 153.40, 148.58, 147.44, 146.99, 140.64, 135.03, 131.08, 130.89, 129.61, 129.13, 129.01, 128.96, 124.19, 122.67, 121.04, 119.48, 109.44, 108.66, 103.72, 70.61, 70.54, 68.85, 68.52, 58.88, 58.83; HR MS(ESI)m/z: calcd for C_28_H_27_O_4_N_6_ClNa [M+Na]^+^ 569.1675, found 569.1678.

[6,7-Bis-(2-methoxy-ethoxy)-quinazolin-4-yl]-{3-[1-(2-bromo-phenyl)-1H-[1,2,3]triazol-4-yl]-phenyl}-amine (e9). Purity 97.7%; m.p.93–97°C; ^1^H NMR (600 MHz, DMSO-d_6_): δ 9.63 (s, 1H), 9.05 (s, 1H), 8.51 (s, 1H), 8.40 (s, 1H), 8.00–7.89 (m, 3H), 7.77 (dd, J = 7.8, 1.5 Hz, 1H), 7.67 (t, J = 7.7 Hz, 2H), 7.60 (t, J = 8.6 Hz, 1H), 7.52 (t, J = 7.9 Hz, 1H), 7.25 (s, 1H), 4.35–4.27 (m, 4H), 3.83–3.75 (m, 4H), 3.39 (s, 3H), 3.37 (s, 3H); ^13^C NMR (150 Hz, DMSO-d_6_): 156.87, 154.10, 153.39, 148.58, 147.40, 146.92, 140.63, 136.72, 134.14, 132.58, 130.94, 129.61, 129.49, 129.24, 124.20, 122.64, 121.02, 119.47, 109.44, 108.64, 103.72, 100.00, 70.61, 70.54, 68.85, 68.52, 58.88, 58.83; HR MS(ESI)m/z: calcd for C_28_H_27_O_4_N_6_BrNa [M+Na]^+^ 613.1169, found 613.1180.

[6,7-Bis-(2-methoxy-ethoxy)-quinazolin-4-yl]-{3-[1-(4-bromo-phenyl)-1H-[1,2,3]triazol-4-yl]-phenyl}-amine (e10). Purity 98.2%; m.p.105–108°C; ^1^H NMR (600 MHz, DMSO-d_6_): δ 9.63 (s, 1H), 9.37 (s, 1H), 8.51 (s, 1H), 8.38 (s, 1H), 7.96 (dd, J = 16.5, 7.6 Hz, 4H), 7.86 (d, J = 8.8 Hz, 2H), 7.66 (d, J = 7.6 Hz, 1H), 7.53 (t, J = 7.9 Hz, 1H), 7.25 (s, 1H), 4.32 (d, J = 24.8 Hz, 4H), 3.79 (d, J = 34.8 Hz, 4H), 3.39 (s, 3H), 3.37 (s, 3H); ^13^C NMR (150 Hz, DMSO-d_6_): 156.86, 154.09, 153.40, 148.58, 147.97, 147.44, 140.63, 136.32, 133.32, 130.82, 129.62, 122.79, 122.38, 121.82, 121.01, 120.19, 119.48, 109.43, 108.66, 103.68, 70.60, 70.54, 68.84, 68.51, 58.88, 58.82; HR MS(ESI)m/z: calcd for C_28_H_27_O_4_N_6_BrNa [M+Na]^+^ 613.1169, found 613.1177.

[6,7-Bis-(2-methoxy-ethoxy)-quinazolin-4-yl]-{3-[1-(2-methoxy-phenyl)-1H-[1,2,3]triazol-4-yl]-phenyl}-amine (e11). Purity 98.7%; m.p.87–90°C; ^1^H NMR (600 MHz, DMSO-d_6_): δ 9.62 (s, 1H), 8.94 (s, 1H), 8.50 (s, 1H), 8.36 (s, 1H), 7.98–7.91 (m, 2H), 7.69 (dd, J = 21.0, 7.7 Hz, 2H), 7.58 (t, J = 7.9 Hz, 1H), 7.50 (t, J = 7.9 Hz, 1H), 7.36 (d, J = 8.2 Hz, 1H), 7.25 (s, 1H), 7.19 (t, J = 7.6 Hz, 1H), 4.32 (d, J = 25.5 Hz, 4H), 3.90 (s, 3H), 3.79 (d, J = 34.6 Hz, 4H), 3.39 (s, 3H), 3.37 (s, 3H); ^13^C NMR (150 Hz, DMSO-d_6_): 156.88, 154.08, 153.41, 152.32, 148.56, 147.45, 146.67, 140.57, 131.39, 131.21, 129.52, 126.43, 126.21, 123.94, 122.54, 121.36, 121.04, 119.43, 113.49, 109.45, 108.67, 103.73, 68.84, 68.51, 58.88, 58.52, 56.65; HR MS(ESI)m/z: calcd for C_29_H_30_O_5_N_6_Na [M+Na]^+^ 565.2170, found 565.2172.

[6,7-Bis-(2-methoxy-ethoxy)-quinazolin-4-yl]-[3-(1-p-tolyl-1H-[1,2,3]triazol-4-yl)-phenyl]-amine (e12). Purity 97.4%; m.p.95–98°C; ^1^H NMR (600 MHz, DMSO-d_6_): δ 9.63 (s, 1H), 9.28 (s, 1H), 8.51 (s, 1H), 8.37 (s, 1H), 7.95 (d, J = 9.9 Hz, 2H), 7.87 (d, J = 8.3 Hz, 2H), 7.67 (d, J = 7.6 Hz, 1H), 7.52 (t, J = 7.9 Hz, 1H), 7.45 (d, J = 8.3 Hz, 2H), 7.25 (s, 1H), 4.32 (d, J = 25.1 Hz, 4H), 3.79 (d, J = 35.1 Hz, 4H), 3.39 (s, 3H), 3.37 (s, 3H), 2.51 (s, 3H); ^13^C NMR (150 Hz, DMSO-d_6_): 156.88, 154.09, 153.40, 148.58, 147.70, 147.40, 140.59, 138.83, 134.89, 131.06, 130.75, 129.57, 122.69, 121.02, 120.36, 120.02, 119.46, 109.43, 108.64, 103.69, 70.60, 70.54, 68.84, 68.52, 58.88, 58.82, 21.07; HR MS(ESI)m/z: calcd for C_29_H_31_O_4_N_6_ [M+H]^+^ 527.2401, found 527.2410.

[6,7-Bis-(2-methoxy-ethoxy)-quinazolin-4-yl]-{3-[1-(3-nitro-phenyl)-1H-[1,2,3]triazol-4-yl]-phenyl}-amine (e13). Purity 99.2%; m.p.98–101°C; ^1^H NMR (600 MHz, DMSO-d_6_): δ 9.64 (s, 1H), 9.60 (s, 1H), 8.83 (t, J = 2.1 Hz, 1H), 8.50 (d, J = 12.4 Hz, 2H), 8.41 (s, 1H), 8.37 (d, J = 7.5 Hz, 1H), 7.99–7.94 (m, 3H), 7.69 (d, J = 7.8 Hz, 1H), 7.55 (t, J = 7.9 Hz, 1H), 7.25 (s, 1H), 4.32 (d, J = 25.9 Hz, 4H), 3.79 (d, J = 36.0 Hz, 4H), 3.39 (s, 3H), 3.37 (s, 3H); ^13^C NMR (150 Hz, DMSO-d_6_): 156.86, 154.09, 153.40, 149.06, 148.59, 147.45, 140.67, 137.70, 132.09, 130.64, 129.68, 126.40, 123.61, 122.92, 121.03, 120.61, 119.55, 115.08, 109.43, 108.66, 103.67, 70.61, 70.54, 68.84, 68.52, 58.88, 58.83; HR MS(ESI)m/z: calcd for C_28_H_27_O_6_N_7_Na [M+Na]^+^ 580.1915, found 580.1923.

[6,7-Bis-(2-methoxy-ethoxy)-quinazolin-4-yl]-{3-[1-(3-ethoxy-phenyl)-1H-[1,2,3]triazol-4-yl]-phenyl}-amine (e14). Purity 98.3%; m.p.110–114°C; ^1^H NMR (600 MHz, DMSO-d_6_): δ 9.63 (s, 1H), 9.36 (s, 1H), 8.51 (s, 1H), 8.37 (s, 1H), 7.95 (d, J = 10.1 Hz, 2H), 7.67 (d, J = 7.7 Hz, 1H), 7.55 (d, J = 36.4 Hz, 4H), 7.25 (s, 1H), 7.08 (d, J = 10.2 Hz, 1H), 4.32 (d, J = 24.9 Hz, 4H), 4.17 (q, J = 7.0 Hz, 2H), 3.79 (d, J = 35.2 Hz, 4H), 3.39 (s, 3H), 3.37 (s, 3H), 1.39 (t, J = 7.0 Hz, 3H); ^13^C NMR (150 Hz, DMSO-d_6_): 159.98, 156.87, 154.08, 153.42, 148.57, 147.84, 147.49, 140.61, 138.13, 131.37, 130.97, 129.59, 122.77, 121.01, 120.19, 119.48, 115.27, 112.27, 109.46, 108.69, 106.46, 103.68, 70.61, 70.54, 68.84, 68.51, 64.12, 58.88, 58.82, 15.02; HR MS(ESI)m/z: calcd for C_30_H_32_O_5_N_6_Na [M+Na]^+^ 579.2326, found 579.2332.

[6,7-Bis-(2-methoxy-ethoxy)-quinazolin-4-yl]-[3-(1-phenyl-1H-[1,2,3]triazol-4-yl)-phenyl]-amine (e15). Purity 96.8%; m.p.137–140°C; ^1^H NMR (600 MHz, DMSO-d_6_): δ 9.63 (s, 1H), 9.34 (s, 1H), 8.50 (s, 1H), 8.38 (s, 1H), 7.99 (d, J = 7.6 Hz, 2H), 7.94 (d, J = 9.8 Hz, 2H), 7.66 (dd, J = 16.3, 8.6 Hz, 3H), 7.53 (d, J = 18.2 Hz, 2H), 7.24 (s, 1H), 4.32 (d, J = 25.9 Hz, 4H), 3.78 (d, J = 35.2 Hz, 4H), 3.39 (s, 3H), 3.36 (s, 3H); ^13^C NMR (150 Hz, DMSO-d_6_): 156.88, 154.08, 153.42, 148.58, 147.81, 147.45, 140.61, 137.13, 130.99, 130.42, 129.59, 129.21, 122.73, 121.03, 120.49, 120.15, 119.48, 109.44, 108.66, 103.69, 70.61, 70.54, 68.84, 68.51, 58.88, 58.82; HR MS(ESI)m/z: calcd for C_28_H_28_O_4_N_6_Na [M+Na]^+^ 535.2064, found 535.2069.

[6,7-Bis-(2-methoxy-ethoxy)-quinazolin-4-yl]-{3-[1-(2-trifluoromethyl-phenyl)-1H-[1,2,3]triazol-4-yl]-phenyl}-amine (e16). Purity 97.7%; m.p.113–116°C; ^1^H NMR (400 MHz, DMSO-d_6_): δ 9.62 (s, 1H), 9.05 (s, 1H), 8.51 (s, 1H), 8.39 (s, 1H), 8.08 (d, J = 7.1 Hz, 1H), 7.93 (d, J = 61.3 Hz, 5H), 7.65 (d, J = 7.8 Hz, 1H), 7.52 (t, J = 7.9 Hz, 1H), 7.24 (s, 1H), 4.32 (d, J = 18.3 Hz, 4H), 3.78 (d, J = 25.0 Hz, 4H), 3.39 (s, 3H), 3.37 (s, 3H); ^13^C NMR (100 Hz, DMSO-d_6_): 156.83, 154.04, 153.35, 148.54, 147.33, 146.97, 140.62, 134.57, 131.77, 130.75, 129.84, 129.63, 127.97, 125.55, 125.24, 124.74, 122.72, 121.94, 121.01, 119.49, 109.39, 108.53, 103.59, 70.57, 70.50, 68.78, 68.47, 58.85, 58.80; HR MS (ESI)m/z: calcd for C_29_H_27_O_4_N_6_F_3_Na [M+Na]^+^ 603.1938, found 603.1945.

[6,7-Bis-(2-methoxy-ethoxy)-quinazolin-4-yl]-{3-[1-(2,4-dimethoxy-phenyl)-1H-[1,2,3]triazol-4-yl]-phenyl}-amine (e17). Purity 98.5%; m.p.85–88°C; ^1^H NMR (400 MHz, DMSO-d_6_): δ 9.66 (s, 1H), 8.88 (s, 1H), 8.55 (s, 1H), 8.39 (s, 1H), 8.02–7.92 (m, 2H), 7.70 (d, J = 7.8 Hz, 1H), 7.63 (d, J = 8.7 Hz, 1H), 7.54 (t, J = 7.9 Hz, 1H), 7.29 (s, 1H), 6.92 (d, J = 2.5 Hz, 1H), 6.78 (dd, J = 8.8, 2.6 Hz, 1H), 4.37 (d, J = 19.0 Hz, 4H), 3.93 (s, 3H), 3.92 (s, 3H), 3.84 (d, J = 25.5 Hz, 4H), 3.44 (s, 3H), 3.42 (s, 3H); ^13^C NMR (100 Hz, DMSO-d_6_): 161.72, 156.84, 154.01, 153.70, 153.37, 148.52, 146.49, 140.50, 131.29, 129.49, 127.48, 124.06, 122.46, 120.99, 119.62, 119.34, 108.60, 105.72, 103.61, 99.99, 70.57, 70.51, 68.79, 68.47, 58.86, 58.80, 56.66, 56.18; HR MS (ESI) m/z: calcd for C_30_H_32_O_6_N_6_Na [M+Na]^+^ 595.2276, found 595.2285.

2-(4-{3-[6,7-Bis-(2-methoxy-ethoxy)-quinazolin-4-ylamino]-phenyl}-[1,2,3]triazol-1-yl)-5-methyl-phenol (e18)¶. Purity 98.8%; m.p.100–103°C; ^1^H NMR (400 MHz, DMSO-d_6_): δ 9.66 (s, 1H), 8.98 (s, 1H), 8.55 (s, 1H), 8.40 (s, 1H), 7.99 (d, J = 12.3 Hz, 2H), 7.73 (dd, J = 14.5, 7.8 Hz, 2H), 7.63 (t, J = 7.9 Hz, 1H), 7.55 (t, J = 7.9 Hz, 1H), 7.41 (d, J = 7.7 Hz, 1H), 7.29 (s, 1H), 7.24 (t, J = 7.6 Hz, 1H), 4.37 (d, J = 19.7 Hz, 4H), 3.95 (s, 3H), 3.83 (d, J = 25.8 Hz, 4H), 3.44 (s, 3H), 3.42 (s, 3H); ^13^C NMR (100 Hz, DMSO-d_6_): 156.85, 154.02, 153.39, 152.29, 148.52, 147.37, 146.65, 140.53, 131.40, 131.18, 129.53, 126.45, 126.17, 123.98, 122.55, 121.35, 121.03, 119.39, 113.45, 109.40, 108.58, 103.60, 70.57, 70.51, 68.78, 68.47, 58.87, 58.81, 56.62; HR MS(ESI)m/z: calcd for C_29_H_30_O_5_N_6_Na [M+Na]^+^ 565.2170, found 565.2175.

N-(3-(1-(3,4-dimethoxyphenyl)-1H-1,2,3-triazol-4-yl)phenyl)-6,7-bis(2-methoxyethoxy)quinazolin-4-amine (e19). Purity 97.9%; m.p.106–109°C; ^1^H NMR (400 MHz, DMSO-d_6_): δ 9.70 (s, 1H), 9.32 (s, 1H), 8.57 (s, 1H), 8.40 (s, 1H), 8.01 (d, J = 10.2 Hz, 2H), 7.71 (d, J = 7.7 Hz, 1H), 7.58 (d, J = 27.3 Hz, 3H), 7.30 (s, 1H), 7.24 (d, J = 8.8 Hz, 1H), 4.37 (d, J = 19.7 Hz, 4H), 3.95 (s, 3H), 3.91 (s, 3H), 3.84 (d, J = 25.9 Hz, 4H), 3.44 (s, 3H), 3.42 (s, 3H).);^13^C NMR (100 Hz, DMSO-d_6_): 157.31, 154.49, 153.84, 150.26, 149.82, 149.00, 147.98, 147.89, 141.03, 131.55, 130.97, 129.97, 123.11, 121.40, 120.60, 119.84, 112.96, 109.86, 109.09, 105.43, 104.09, 71.03, 70.96, 69.26, 68.93, 59.30, 59.24, 56.81, 56.73; HR MS(ESI) m/z: calcd for C_30_H_32_O_6_N_6_Na [M+Na]^+^ 595.2276, found 595.2281.

[6,7-Bis-(2-methoxy-ethoxy)-quinazolin-4-yl]-[3-(1-phenethyl-1H-[1,2,3]triazol-4-yl)-phenyl]-amine (e20). Purity 98.4%; m.p.109–112°C; ^1^H NMR (600 MHz, DMSO-d_6_): δ 9.56 (s, 1H), 8.53 (s, 1H), 8.49 (s, 1H), 8.24 (s, 1H), 7.93 (s, 1H), 7.89 (d, J = 8.9 Hz, 1H), 7.51 (d, J = 7.7 Hz, 1H), 7.45 (t, J = 7.8 Hz, 1H), 7.29 (t, J = 7.4 Hz, 2H), 7.22 (dd, J = 13.1, 6.9 Hz, 4H), 4.68 (t, J = 7.3 Hz, 2H), 4.31 (d, J = 22.8 Hz, 4H), 3.78 (d, J = 32.9 Hz, 4H), 3.38 (s, 3H), 3.36 (s, 3H), 3.24 (t, J = 7.3 Hz, 2H); ^13^C NMR (150 Hz, DMSO-d_6_): 156.84, 154.06, 153.39, 148.56, 147.42, 146.59, 140.51, 138.11, 131.54, 129.48, 129.17, 127.08, 122.19, 121.88, 120.69, 119.17, 109.43, 108.66, 103.68, 70.60, 70.53, 68.83, 68.51, 58.87, 58.82, 51.12, 36.04; HR MS(ESI)m/z: calcd for C_30_H_32_O_4_N_6_Na [M+Na]^+^563.2377, found 563.2381.

### Biological Assay

#### Cell Culture

The human NSCLC cells PC-9, H460, A549 and H1975 were cultured in RPMI-1640 complete growth medium containing 100 U/ml penicillin–streptomycin and 10% FBS. The cells were incubated in a humidified atmosphere at 37°C containing 5% CO_2_. The compounds were dissolved in DMSO to make a 20 μM stock solution and diluted to the concentration of working solutions with complete growth medium before administration.

#### CCK-8 Assay

Cells were seeded in 96-well plates: 3,000–5,000 cells/well in 100 ul. One day after seeding, the test compounds at concentration between 1.0 and 16.0 μ M or 0.1% DMSO (as control) were added to cells in triplicate. 48 h later, CCK-8 was added and incubated the plate for 1–4 h in the incubator. The absorbance at 450 nm were measured using a microplate reader (Bio-Tek). The concentration causing 50% inhibition of cell growth (IC_50_) was determined by Graph Pad Prism 8.0 software.

#### Flow Cytometry Detection for Cell Apoptosis

Cell-apoptosis analysis was carried out by flow cytometry using the Annexin V/PI apoptosis kit according to the manufacturer’s manual. Briefly, PC-9 or H460 (3 × 10^4^–5 × 10^4^/well) cells were seeded in 12-well plates for 24 h, and then treated with 0.1% DMSO (as control) or various concentrations of compound e4 or e12 for 48 h, respectively. Cells were harvested, washed with PBS, and then incubated with 100ul of 1X Annexin V binding buffer containing 1 μl PI and 1 μl FITC Annexin V for 15 min at RT in the dark. 200 µl of 1X binding buffer were added to each tube for flow cytometry analysis (BD LSRFortessa™ Flow Cytometer). The percentages of apoptotic cells were analyzed using FlowJo soft.

#### Hoechst Staining for Cell Apoptosis

PC-9 cells were seeded in 96-well plates, then treated with 0.1% DMSO (as control) or various concentrations of compound e4 or e12 for 48 h. Culture medium from the cells was removed and replaced with medium containing dye Hoechst 33342. Cells were incubated at 37°C for 5–15 min, washed with PBS twice, and then observed and imaged under the Nikon Ti-U microscope*.*

#### Flow Cytometry Detection for Cell Cycle

PC-9 or H460 (2 × 10^5^–3 × 10^5^/well) cells were seeded in six-well plates for 24 h, and then treated with 0.1% DMSO (as control), various concentrations of either compound e4 or e12 for 24 h (PC-9) or 12 h (H460), respectively. The treated cells were harvested, washed with PBS, and then stained using the cell-cycle staining kit according to the manufacturer’s manual. The distribution of cell-cycle phases with different DNA contents was determined by flow cytometry (BD LSRFortessa™ Flow Cytometer) and analyzed using ModFit LT software.

#### qPCR Assay for Cell Cycle-Related Gene Analysis

PC-9 cells were incubated in 12-well plates for 24 h, and then treated with 0.1% DMSO, various concentrations of either compound e4 or e12 for 16 h. Total RNA of cells was extracted and cDNA was synthesized. The primers for the target products were designed as in Table 3.

**TABLE 3:** Primer sequences for qPCR analysis.

CCND1	Forward	AGC​TGT​GCA​TCT​ACA​CCG​AC
	Reverse	GAA​ATC​GTG​CGG​GGT​CAT​TG
CDK6	Forward	GTG​ACC​AGC​AGC​GGA​CAA​AT
	Reverse	TGG​ACT​GGA​GCA​AGA​CTT​CG
CDK2	Forward	GCT​TTT​GGA​GTC​CCT​GTT​CG
	Reverse	GCG​AGT​CAC​CAT​CTC​AGC​AA
CCNA(2)	Forward	CGG​TAC​TGA​AGT​CCG​GGA​AC
	Reverse	CAT​GAA​TGG​TGA​ACG​CAG​GC
CCNB(1)	Forward	GCA​GCA​GGA​GCT​TTT​TGC​TT
	Reverse	CCA​GGT​GCT​GCA​TAA​CTG​GA
β-Actin	Forward	CAATGTGGCCGAGGACTT TG
	Reverse	CAT​TCT​CCT​TAG​AGA​GAA​GTG​G

#### *In Vivo* Antitumor Activity Study

PC-9 xenografts were established by subcutaneous transplantation of cancer cells into the flanks of NSG mice (male, 4–6-week old). Once the tumors in the transplanted mice become palpable, the mice were divided randomly into three groups: one group was injected intraperitoneally with vehicle (5% DMSO, 30% PEG300 and 65% ddH_2_O; qd), one with compound e4 (25 mg/kg, qd) and one with Erlotinib (25 mg/kg, qd) in vehicle every day for 7 consecutive days. Tumor volumes were measured using calipers and calculated with the formula: V = length × width^2^/2. The mouse body mass was recorded as well. 19 days after inoculation, the mice were sacrificed and the tumors were harvested and weighed.

#### EGFR Kinase Assay

Kinase inhibitory activities of compounds were evaluated using the Enzyme-linked immunosorbent assay (ELISA). The kinase enzyme of EGFR was purchased from Carna Bioscience (Kobe, Japan). A total of 10 ng/ml anti phosphotyrosine (PY713) antibody (abcam, Cambridge Science Park, United Kingdom) was precoated in 96-well ELISA plates. Active kinases were incubated with indicated drugs in 1 X reaction buffer (50 mmol/L HEPES pH 7.4, 20 mmol/L MgCl_2_, 0.1 mmol/L MnCl_2_, 1 mmol/L DTT) containing 20 μmol/L substrate (NH2-ETVYSEVRK-biotin) at 25°C for 1 h. Then, a total of 3 μmol/L ATP was added and the reaction was continued for 2 h. The products of reaction were transferred into 96-well ELISA plates containing antibody and incubated at 25°C for 30 min. After incubation, the wells were washed with PBS and then incubated with horseradish peroxidase (HRP)-conjugated streptavidin. The wells were visualized using 3,3′,5,5′-tetramethylbenzidine (TMB), and chromogenic reaction was ended with 2 mol/L H_2_SO_4_, the absorbance was read with a multimode plate reader (PerkinElmer, United States) at 450 nm.

#### Western Blot Analysis

H460 cells were seeded in six-well plates with serum-free medium overnight, and then treated complete medium containing 0.1% DMSO, various concentrations (0.1, 1 and 10 μM) of either compound e4 or e12 for 2 h, respectively. Complete medium with or without Erlotinib (10 μM) were used as control. Then the H460 cells were treated with complete medium containing EGF (10 ng/ml) for 5 min. Then the cells were harvested and washed with PBS, centrifuged, resuspended in 100–200 μl of RIPA lysis buffer containing Protease and Phosphatase Inhibitor Cocktail (1X), and then centrifuged for 5 min at 12,000 rpm at 4°C. The supernatant was used for protein analysis. Total protein extracts were separated by 7.5% SDS polyacrylamide gel electrophoresis and transferred onto NC membranes. Membrane was blocked with 5% skimmed milk or BSA in TBS-T (10 mM Tris [pH 7.4], 150 mM NaCl, and 0.1% Tween20) for 1 h at RT, then incubated with a 1:1,000 dilution of anti-EGFR (D38B1), anti-Akt (pan) (C67E7),anti-p-EGFR (Tyr1068) andanti-AKT1 (phospho S473) [EP2109Y] primary antibodyor 1:5,000 dilution of anti-β-Actin (8H10D10) primary antibody overnight at 4°C. Then the membrane was washed 5 min (X3) and incubated at 1:3,000 dilution of anti-rabbit secondary antibodies or 1:1,000 dilution of anti-mouse secondary antibodies for 1 h at RT. After washing with TBST 5 min (X3), protein bands were developed by chemiluminescence. The protein levels were quantified by the gray values of the bands in the resulting images by ImageJ, and the control group was used as the standard. For PC-9, cells were incubated in six-well plates with complete medium overnight, and then treated with 0.1% DMSO, various concentrations (0.1, 1 and 10 μM) of either compound e4 or e12 for 12 h. Erlotinib (10 μM) was used as control. The PC-9 cells were harvested and analyzed as above.

### Statistical Analysis

All values are presented as means ± SD. Significant differences were evaluated using GraphPad Prism 8 software. The Student’s *t*-test was used to determine significant differences between two groups. A two-tailed *p*-value of <0.05 was considered statistically significant in all experiments.

## Data Availability

The original contributions presented in the study are included in the article/Supplementary Material, further inquiries can be directed to the corresponding authors.
