# Supplementary figures and images for: Discovery of a Series of 1,2,3-Triazole-Containing Erlotinib Derivatives With Potent Anti-Tumor Activities Against Non-Small Cell Lung Cancer
Source: Front Chem. 2022 Jan 7;9:789030. doi: 10.3389/fchem.2021.789030 (PMC8776995; doi:10.3389/fchem.2021.789030)

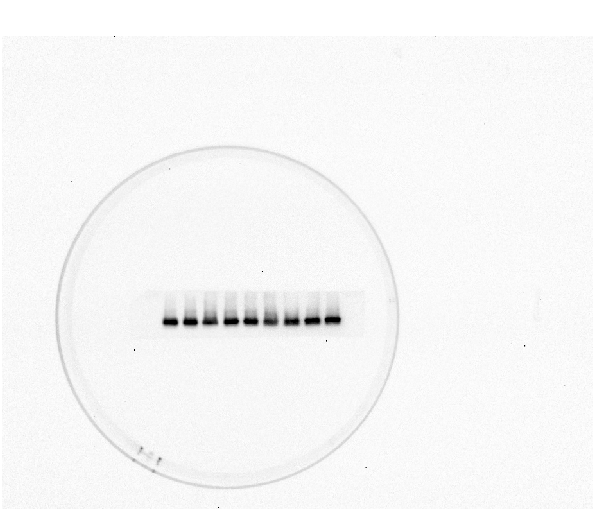

Supplement: Supplementary file 2 [file DataSheet14.ZIP › 7. Western blot pictures/20200430 SG H460 e AKT_18.tif]

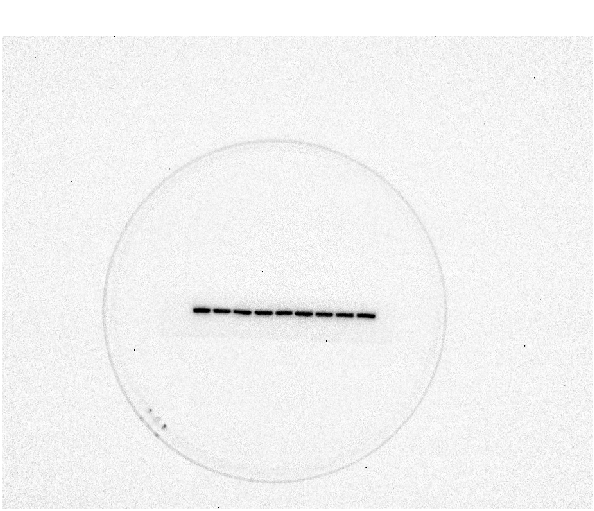

Supplement: Supplementary file 2 [file DataSheet14.ZIP › 7. Western blot pictures/20200430 SG H460 e b-Actin_06.tif]

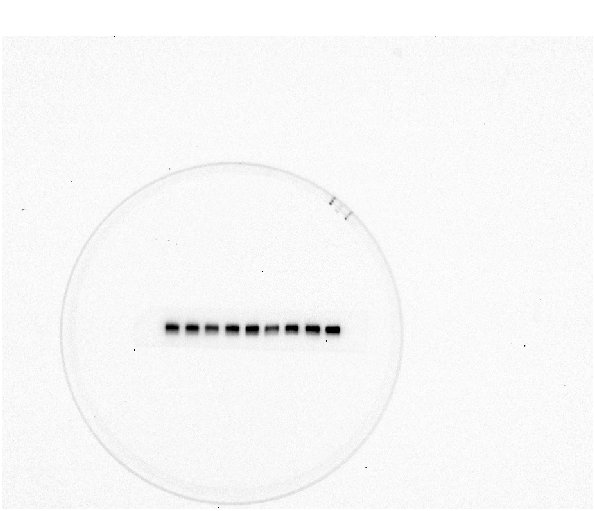

Supplement: Supplementary file 2 [file DataSheet14.ZIP › 7. Western blot pictures/20200430 SG H460 e EGFR_10.tif]

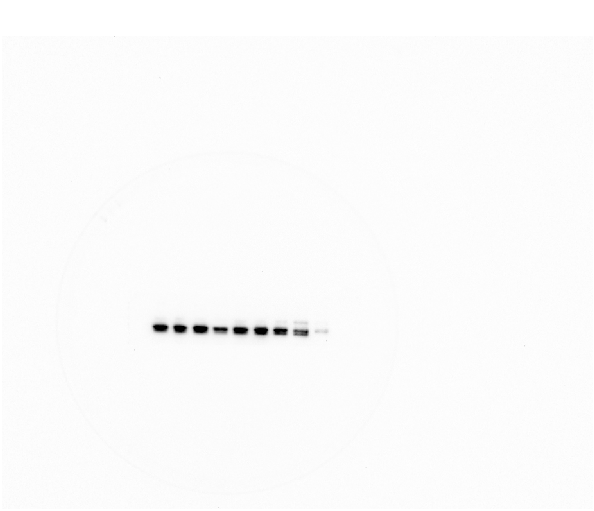

Supplement: Supplementary file 2 [file DataSheet14.ZIP › 7. Western blot pictures/20200430 SG H460 e p_AKT_4.tif]

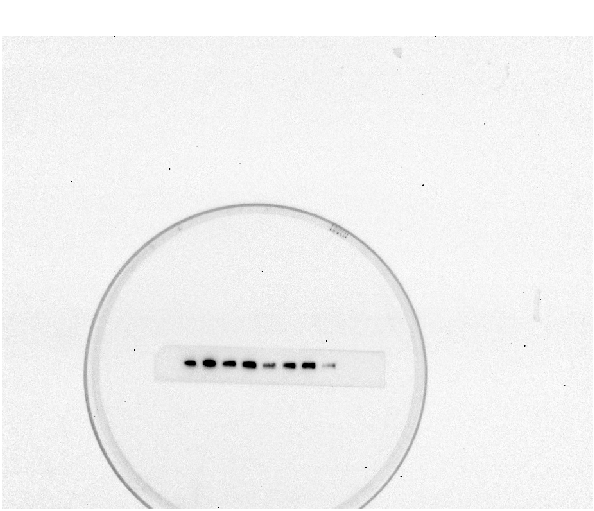

Supplement: Supplementary file 2 [file DataSheet14.ZIP › 7. Western blot pictures/20200430 SG H460 e P_EGFR_12.tif]

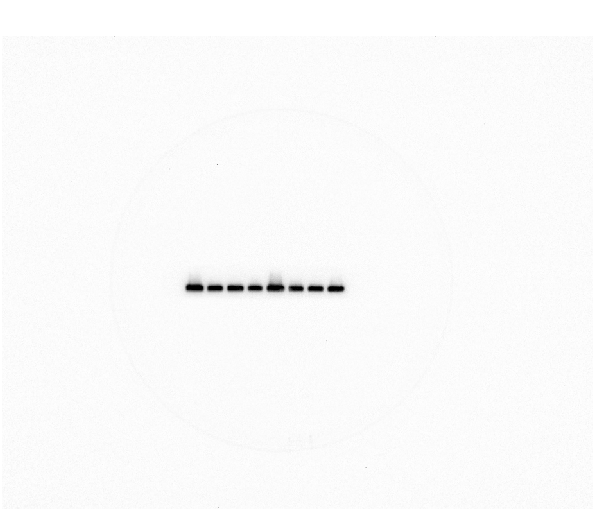

Supplement: Supplementary file 2 [file DataSheet14.ZIP › 7. Western blot pictures/20200503 SG PC-9 e AKT_05.tif]

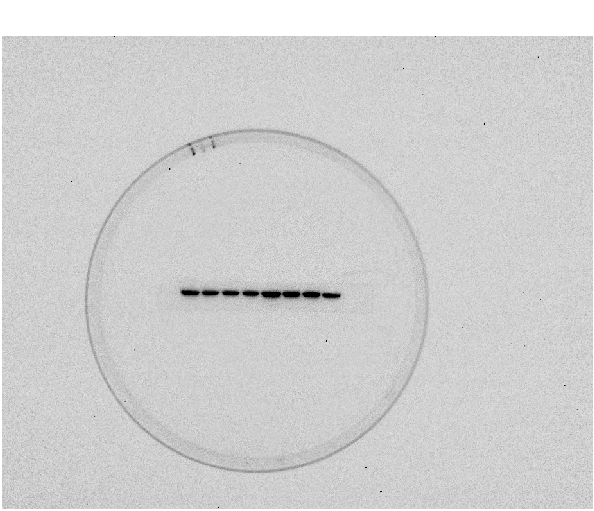

Supplement: Supplementary file 2 [file DataSheet14.ZIP › 7. Western blot pictures/20200503 SG PC-9 e b-Actin_10.tif]

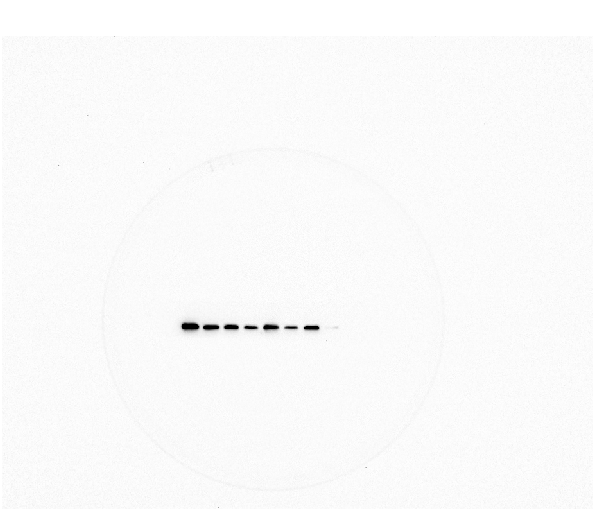

Supplement: Supplementary file 2 [file DataSheet14.ZIP › 7. Western blot pictures/20200503 SG PC-9 e p-AKT_08.tif]

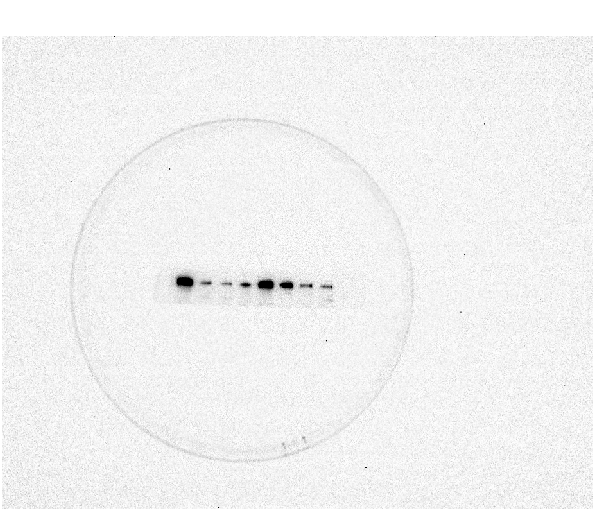

Supplement: Supplementary file 2 [file DataSheet14.ZIP › 7. Western blot pictures/20200503 SG PC-9 e p-EGFR_7.tif]

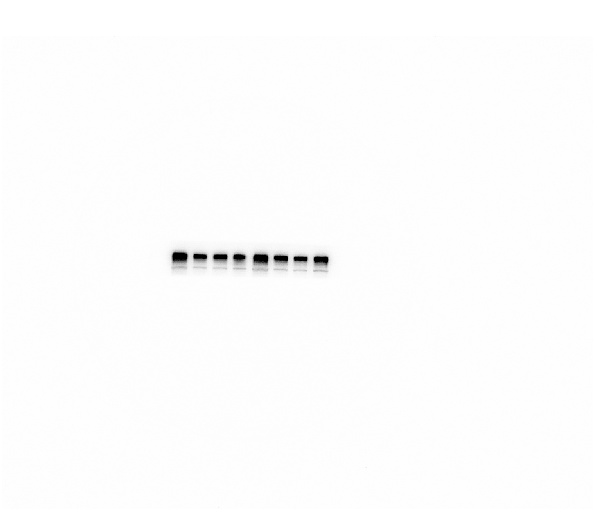

Supplement: Supplementary file 2 [file DataSheet14.ZIP › 7. Western blot pictures/2020503 SG PC-9 e EGFR_3.tif]

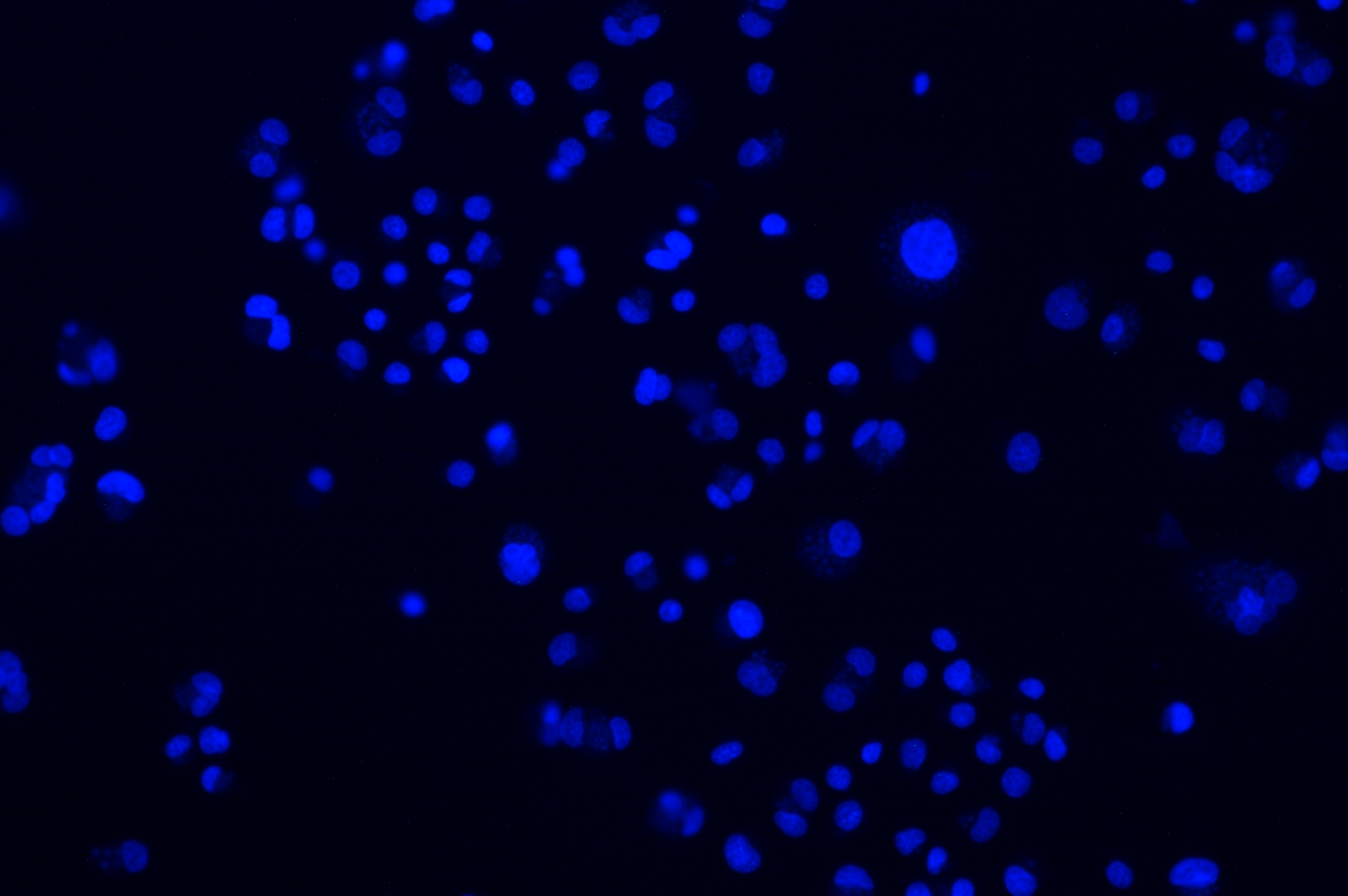

Supplement: Supplementary file 14 [file DataSheet6.ZIP › 3. PC-9 Hoechst staining/PC-9 Hoechst staining e12 12uM 2.tif]

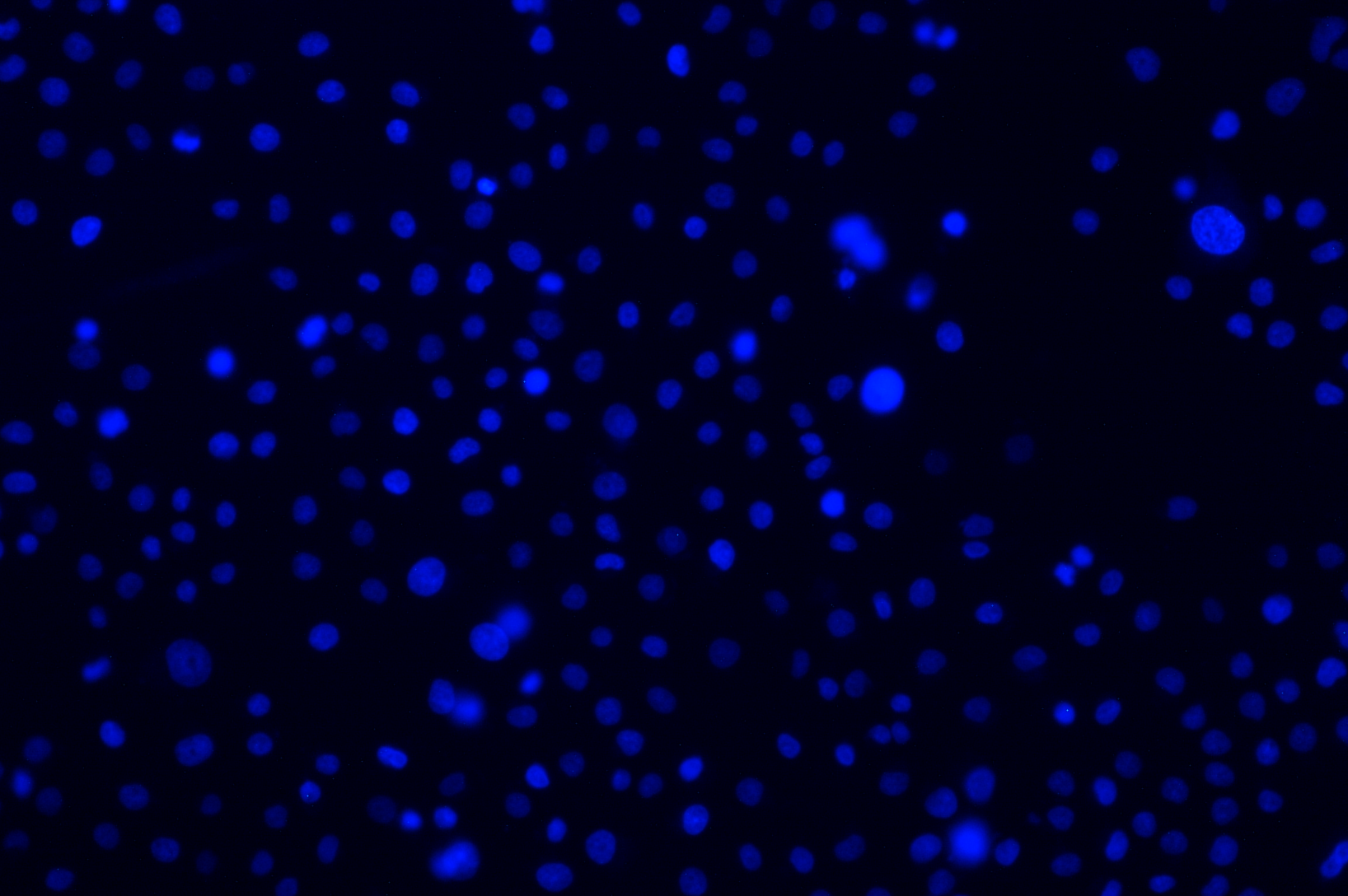

Supplement: Supplementary file 14 [file DataSheet6.ZIP › 3. PC-9 Hoechst staining/PC-9 Hoechst staining e12 4uM 5.tif]

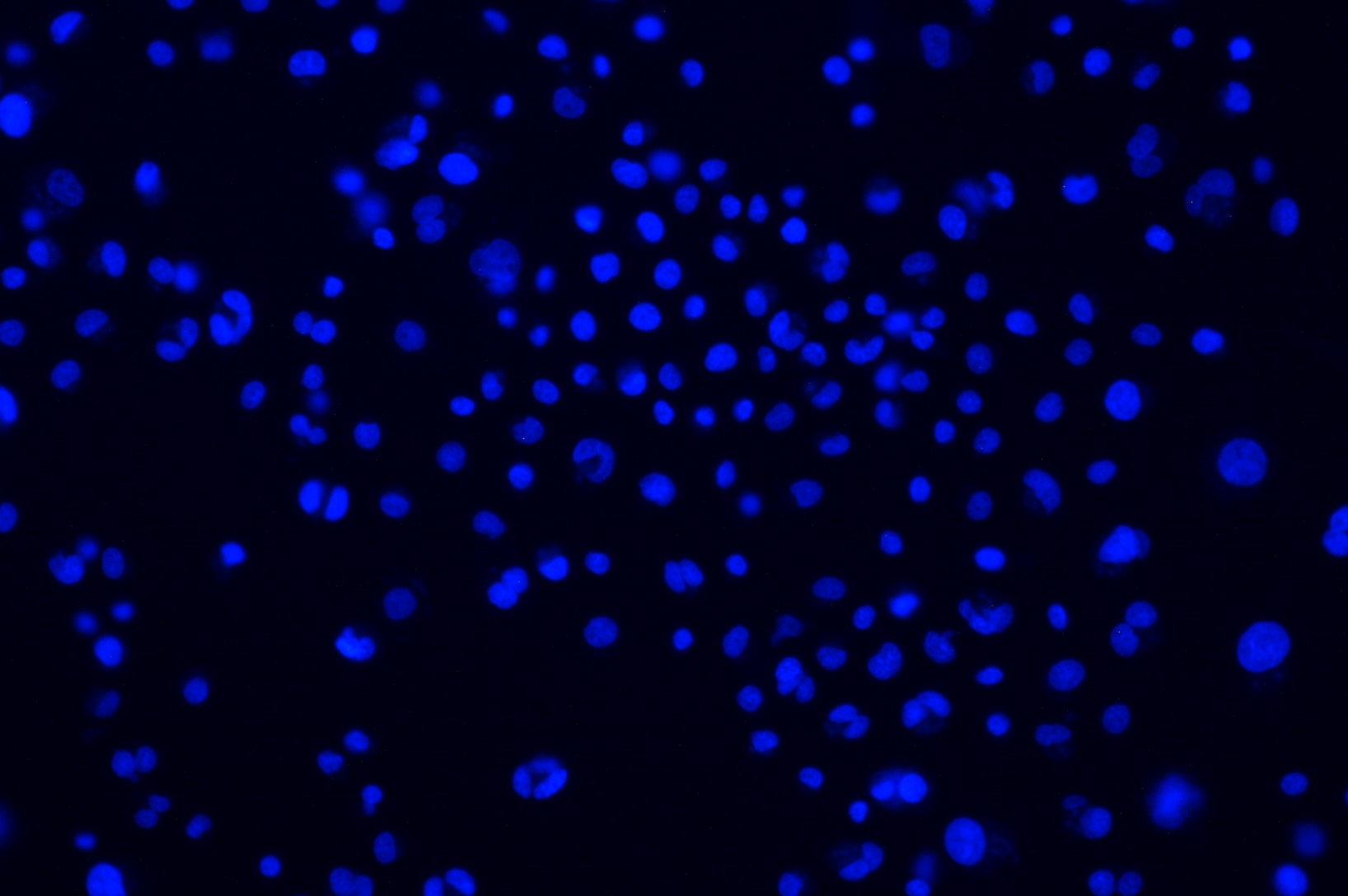

Supplement: Supplementary file 14 [file DataSheet6.ZIP › 3. PC-9 Hoechst staining/PC-9 Hoechst staining e12 8uM 7.tif]

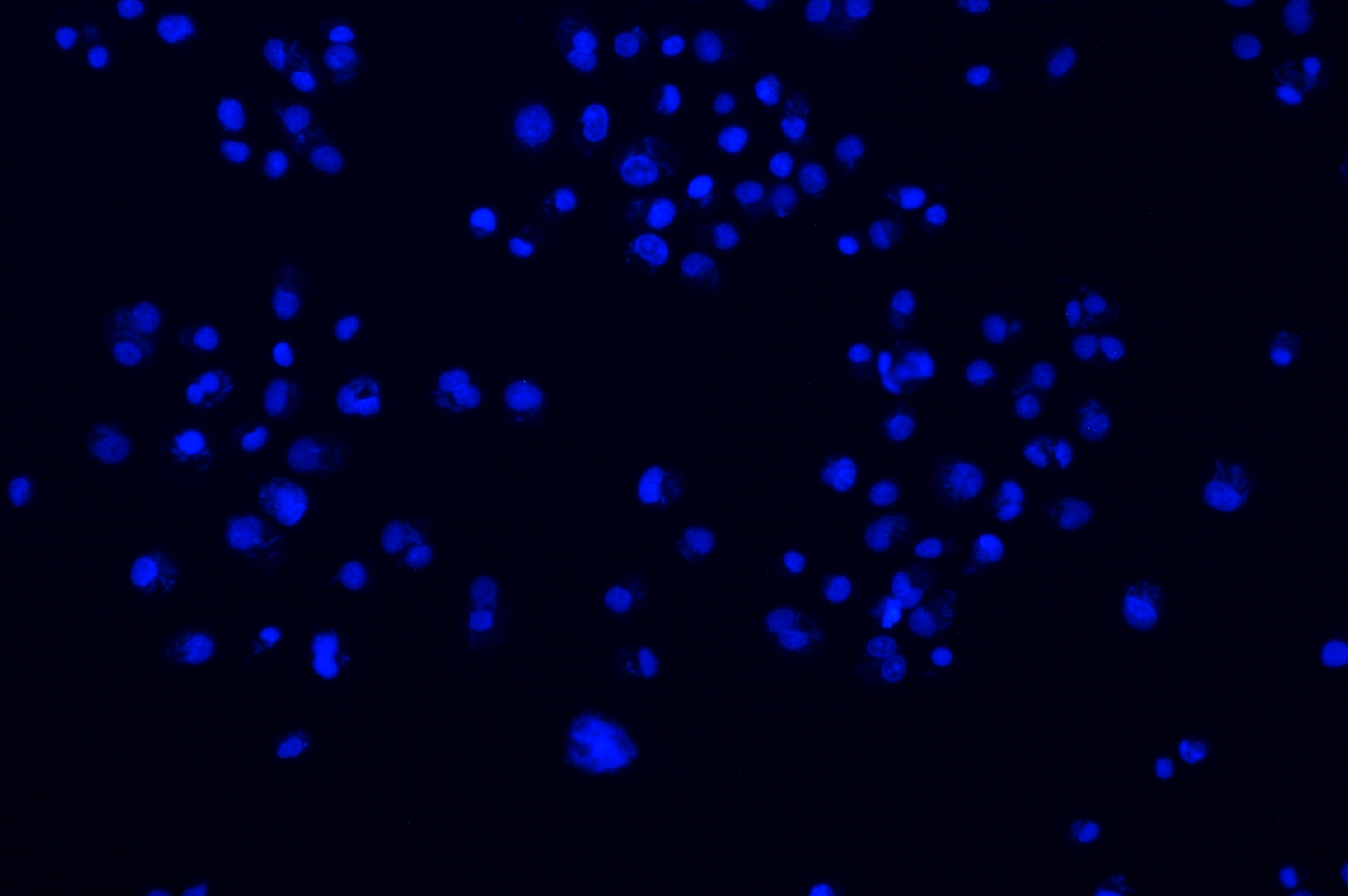

Supplement: Supplementary file 14 [file DataSheet6.ZIP › 3. PC-9 Hoechst staining/PC-9 Hoechst staining e4 12uM.tif]

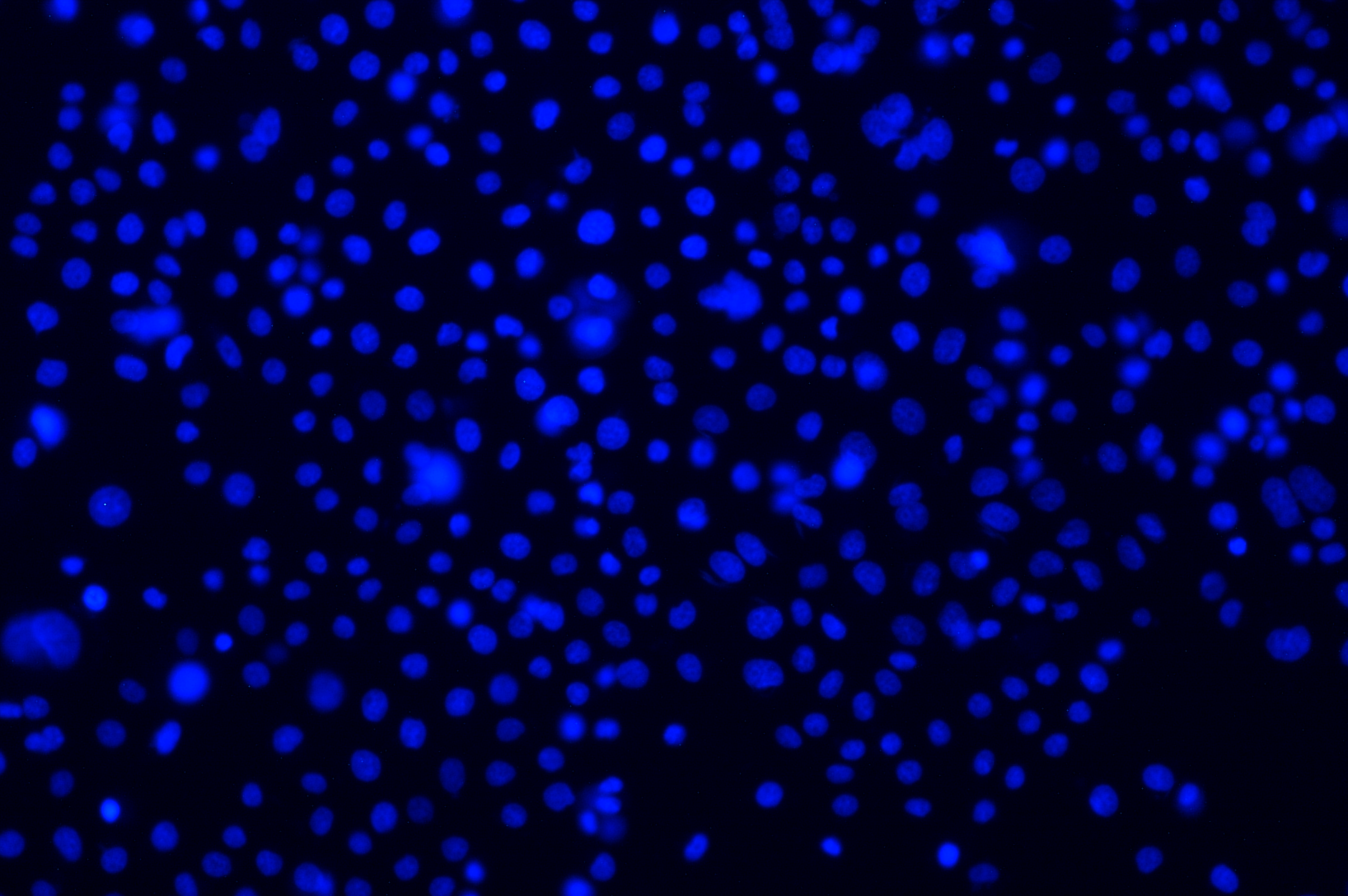

Supplement: Supplementary file 14 [file DataSheet6.ZIP › 3. PC-9 Hoechst staining/PC-9 Hoechst staining e4 4uM 2.tif]

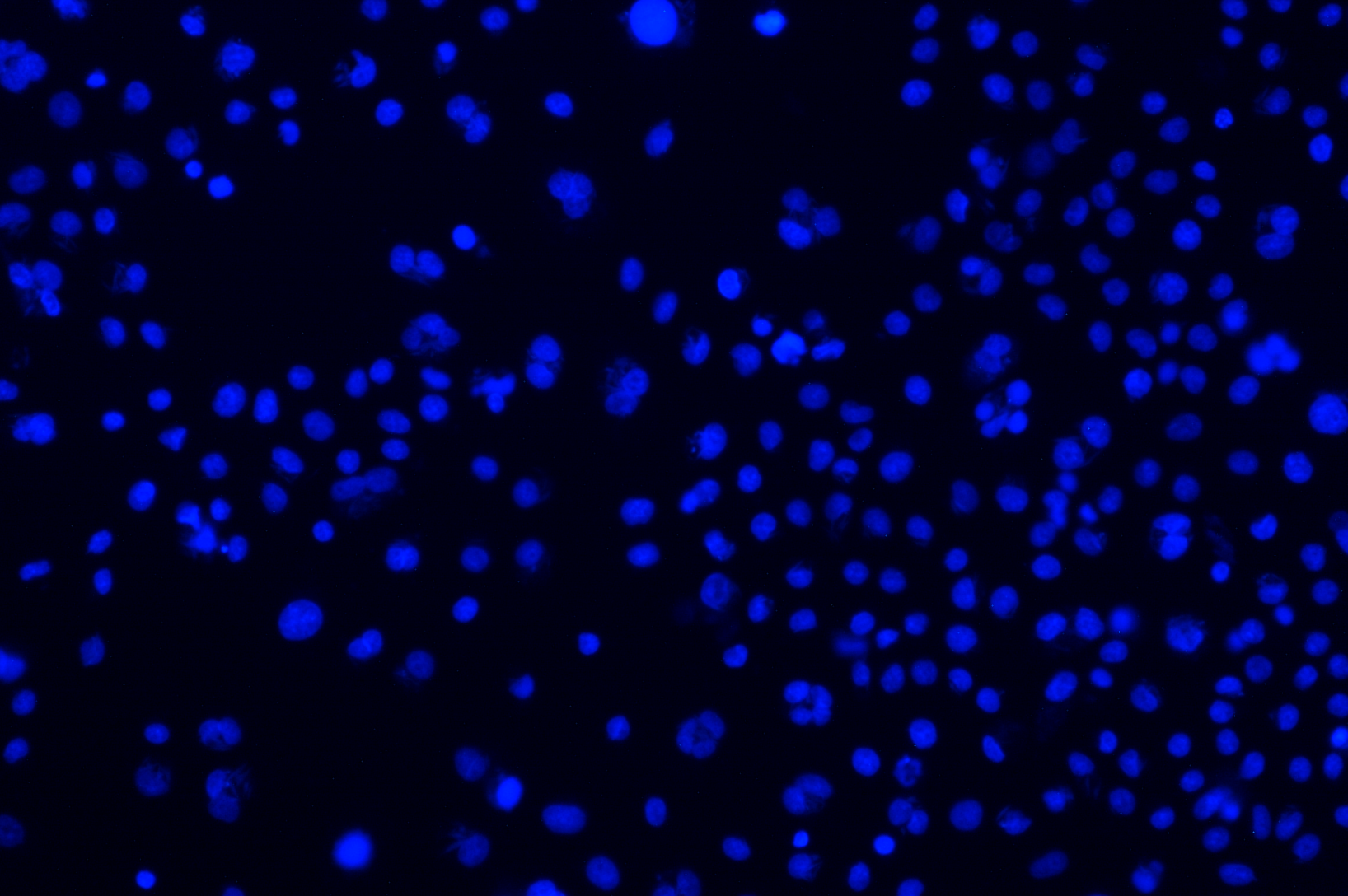

Supplement: Supplementary file 14 [file DataSheet6.ZIP › 3. PC-9 Hoechst staining/PC-9 Hoechst staining e4 8uM 3.tif]

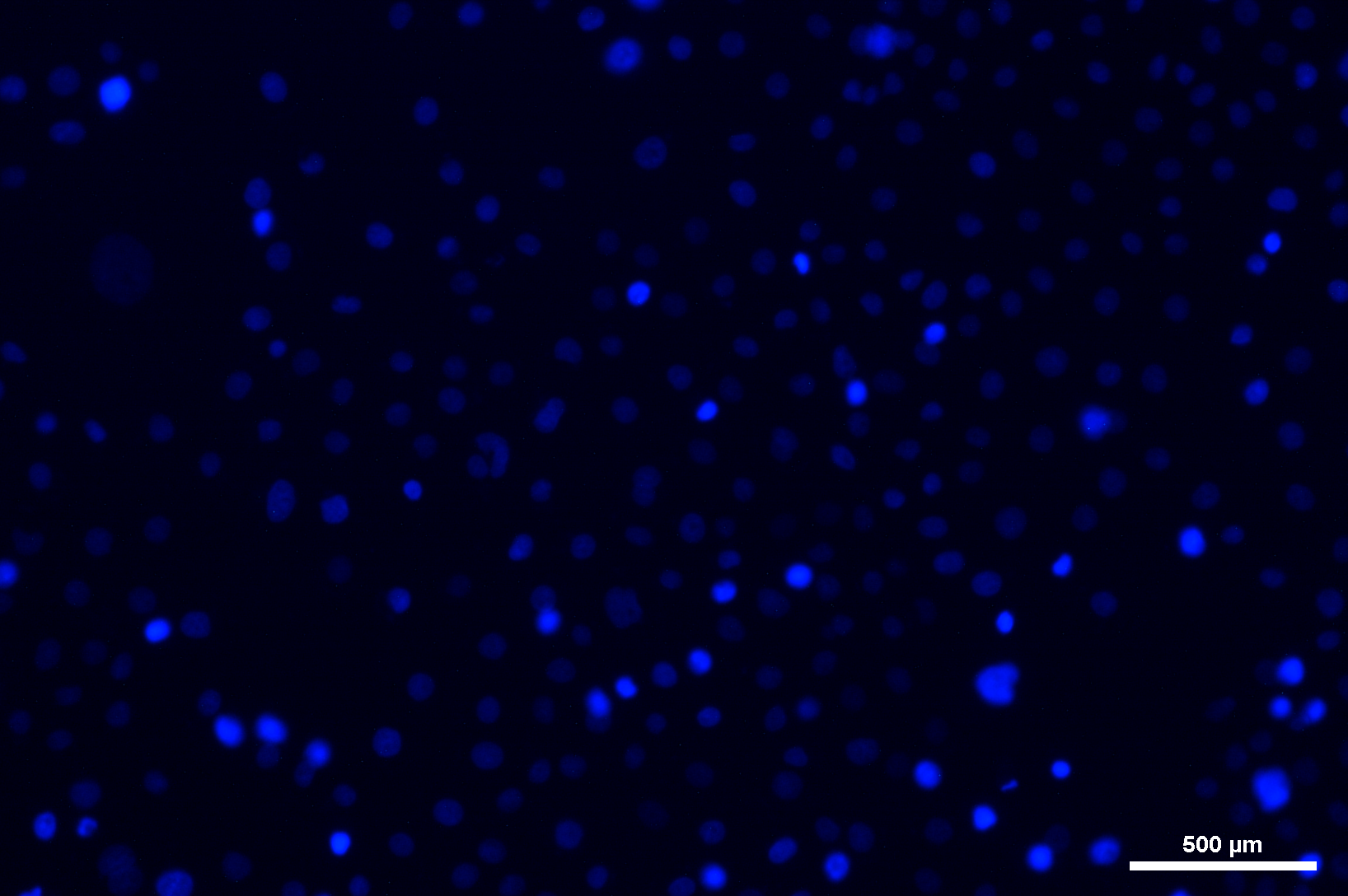

Supplement: Supplementary file 14 [file DataSheet6.ZIP › 3. PC-9 Hoechst staining/PC-9 Hoechst staining NC1 1.tif]

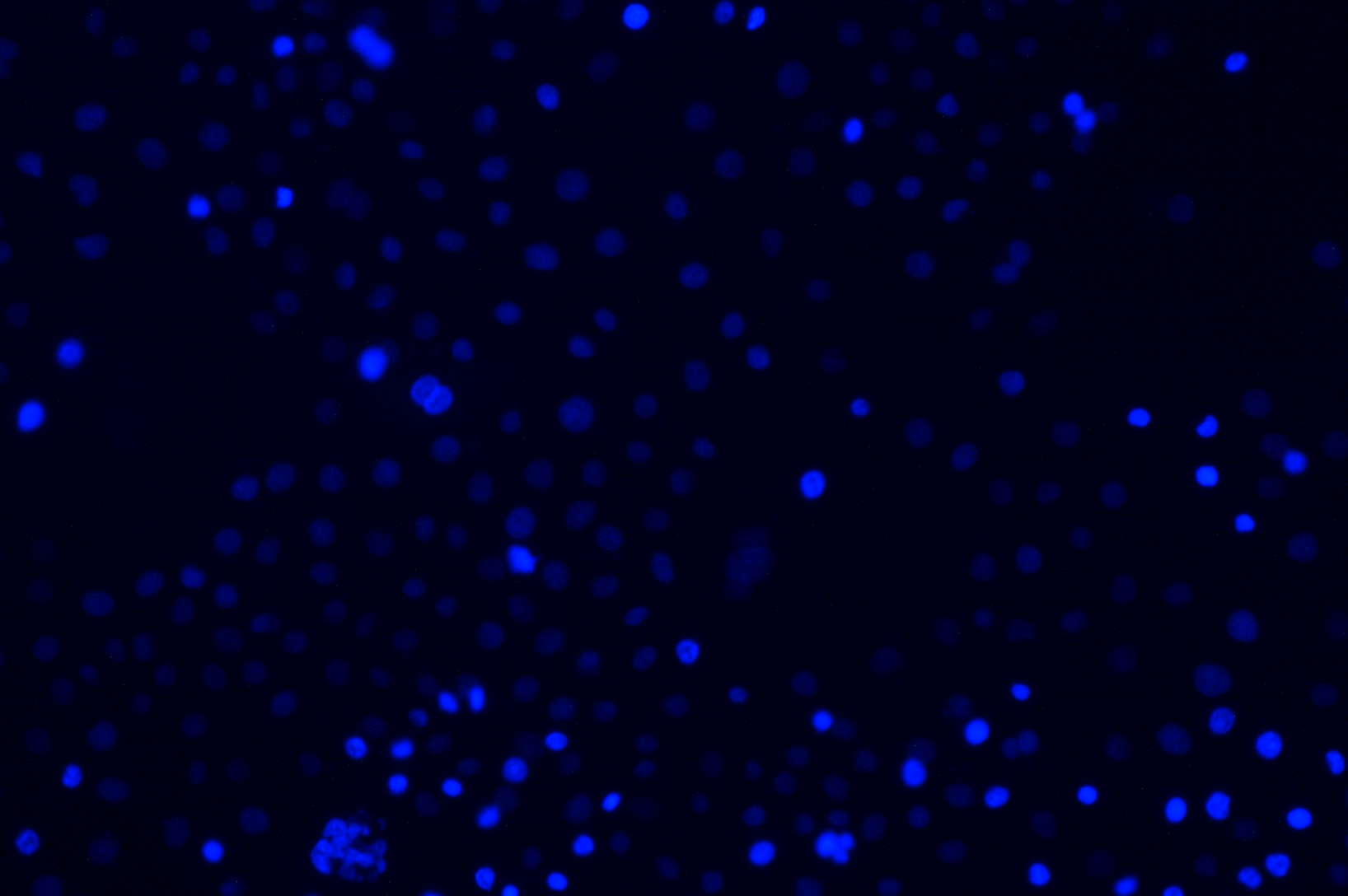

Supplement: Supplementary file 14 [file DataSheet6.ZIP › 3. PC-9 Hoechst staining/PC-9 Hoechst staining NC2 3.tif]

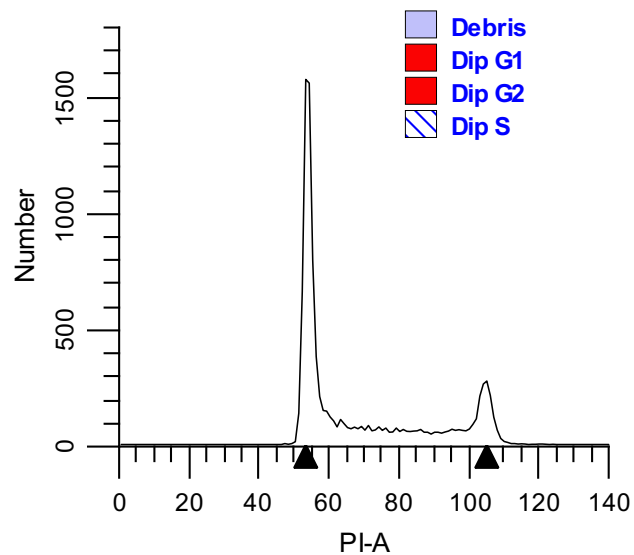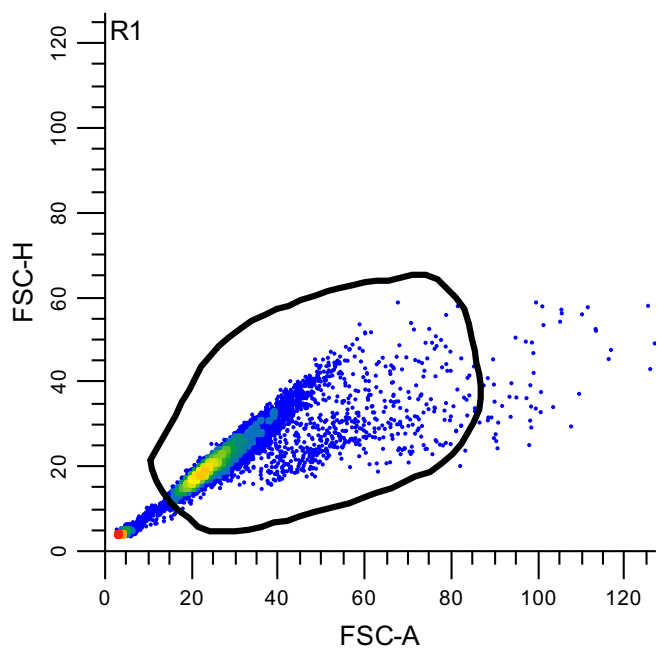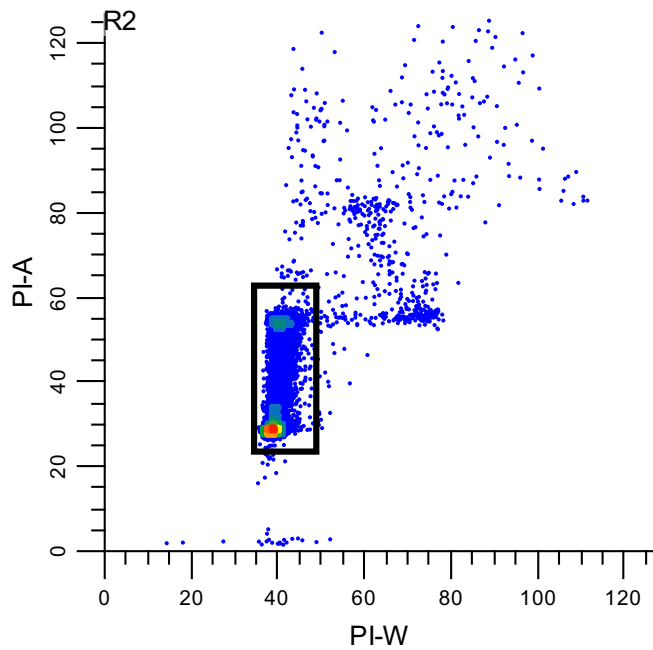

Supplement: Supplementary file 22 [file DataSheet7.zip › H460 Cell cycle-1/rpt_20191202 H460 12h_460 E4 4UM_002.fcs.pdf]
