# Supplementary material for: Discovery of a Series of 1,2,3-Triazole-Containing Erlotinib Derivatives With Potent Anti-Tumor Activities Against Non-Small Cell Lung Cancer
Source: Front Chem. 2022 Jan 7;9:789030. doi: 10.3389/fchem.2021.789030 (PMC8776995; doi:10.3389/fchem.2021.789030)

File analyzed: 20200919 PC-9 24H_001_NC_001.fcs
Date analyzed: 19-Sep-2020
Model: 1Dn0n_DSD
Analysis type: Manual analysis
Auto Linearity: No

Ploidy Mode: First cycle is diploid

Diploid: 100.00 %
  Dip G1: 49.08 % at 59.88
  Dip G2: 13.40 % at 116.77
  Dip S: 37.53 %   G2/G1: 1.95
  %CV: 2.01

Total S-Phase: 37.53 %
Total B.A.D.: 0.00 %   no aggs

Debris: 0.07 %
Aggregates: %
Modeled events: 9503
All cycle events: 9497
Cycle events per channel: 164
RCS: 2.452

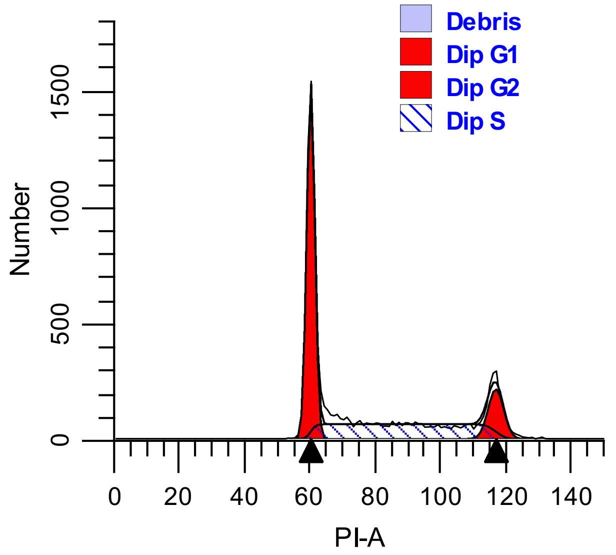

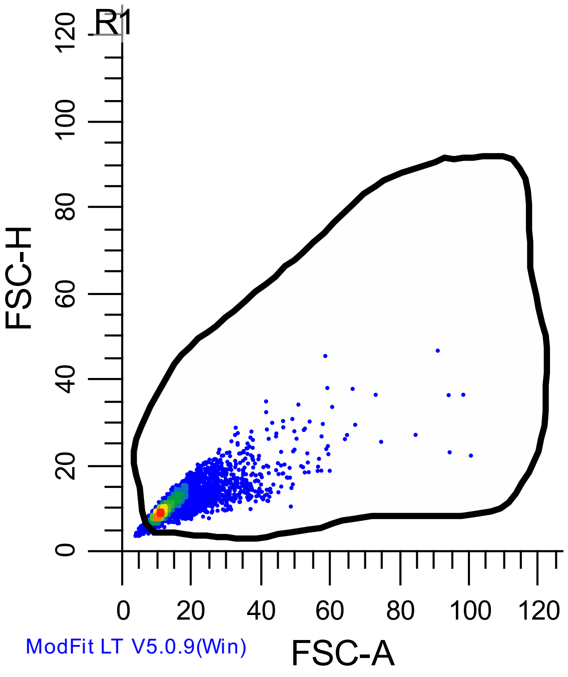

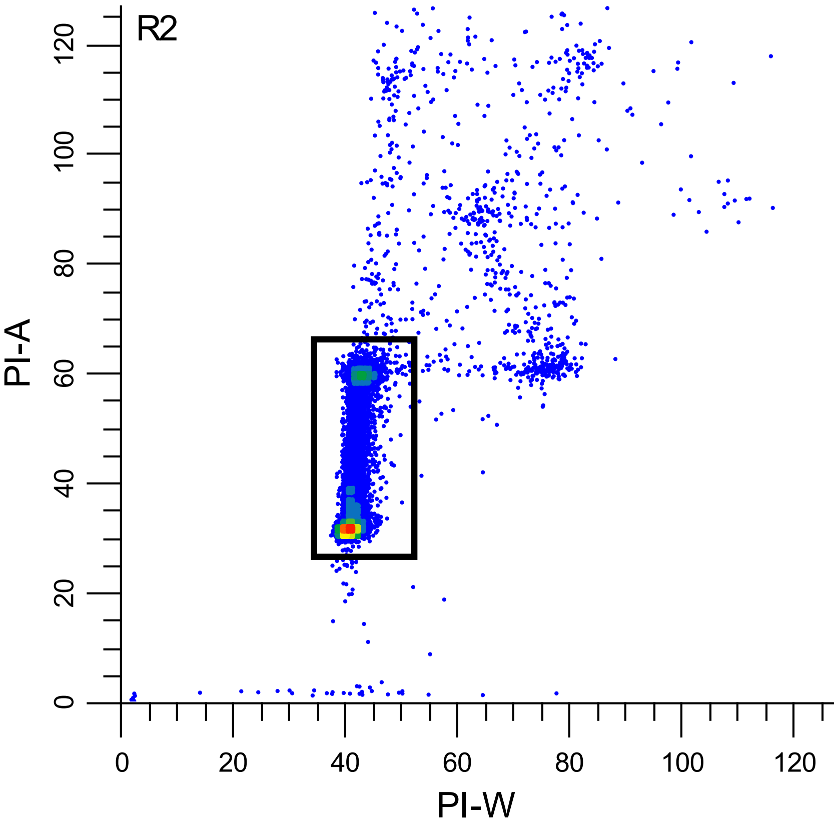

Supplement: Supplementary file 3 [file DataSheet11.zip › PC-9 Cell cycle-2/rpt_20200919 PC-9 24H_001_NC_001.fcs.pdf]
