# Supplementary material for: Discovery of a Series of 1,2,3-Triazole-Containing Erlotinib Derivatives With Potent Anti-Tumor Activities Against Non-Small Cell Lung Cancer
Source: Front Chem. 2022 Jan 7;9:789030. doi: 10.3389/fchem.2021.789030 (PMC8776995; doi:10.3389/fchem.2021.789030)
Supplement: Supplementary file 4 [file DataSheet8.zip › H460 Cell cycle-2/rpt_20200810 h460 e 12h_e4 12uM_004.fcs.pdf]

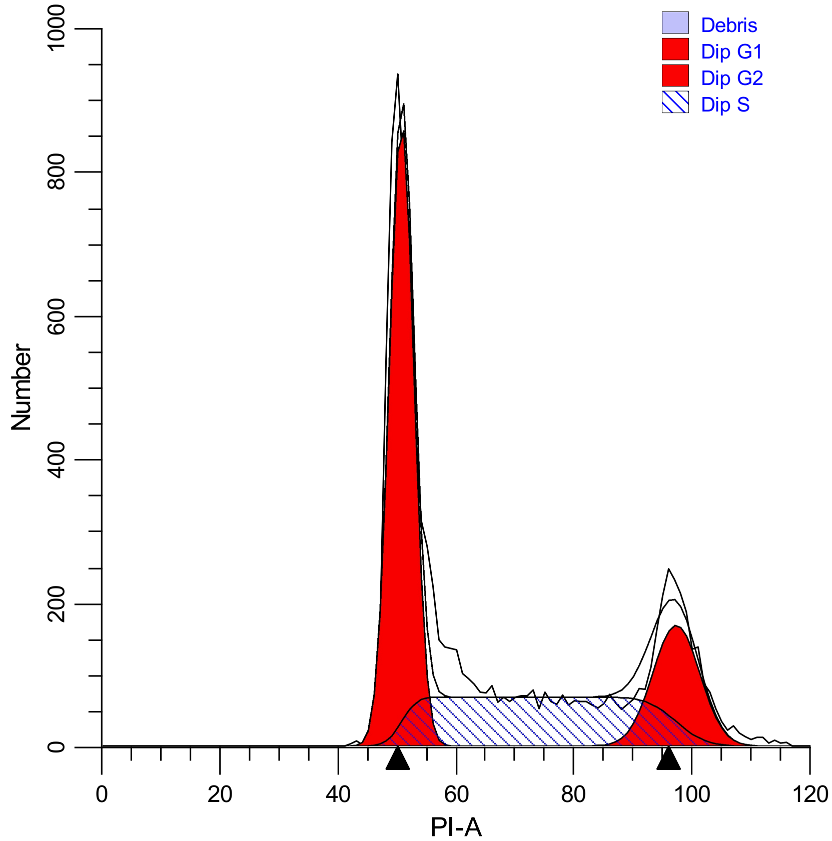

File analyzed: 20200810 h460 e 12h_e4 12uM_0
Date analyzed: 11-Aug-2020
Model: 1Dn0n_DSD
Analysis type: Manual analysis
Auto Linearity: No

Ploidy Mode: First cycle is diploid

Diploid: 100.00 %
Dip G1: 48.29 % at 50.64
Dip G2: 17.85 % at 97.24
Dip S: 33.86 %   G2/G1: 1.92
%CV: 4.05

Total S-Phase: 33.86 %
Total B.A.D.: 0.00 %   no aggs

Debris: 0.05 %
Aggregates: %
Modeled events: 9361
All cycle events: 9356
Cycle events per channel: 197
RCS: 6.129

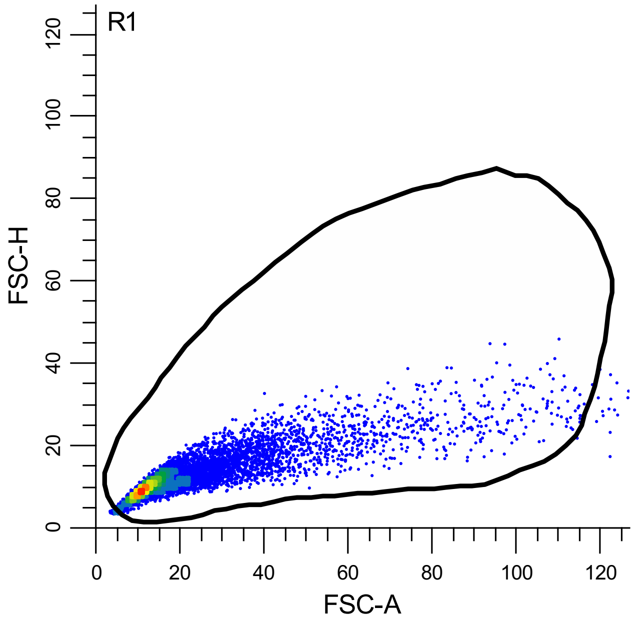

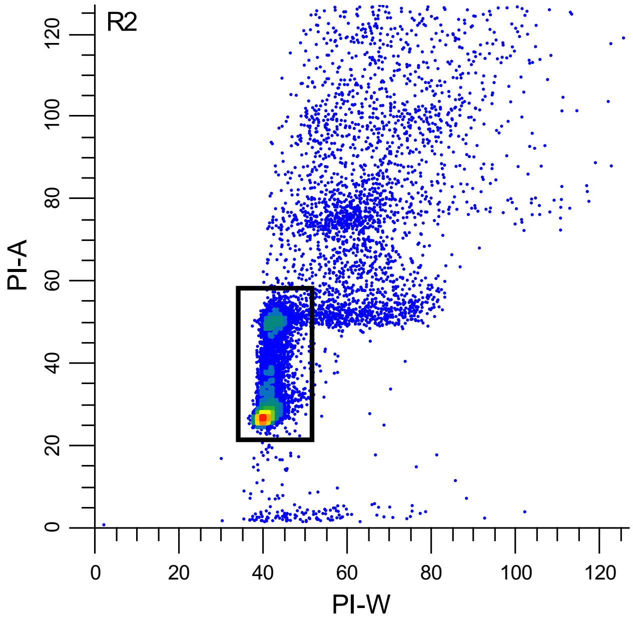

ModFit LT V5.0.9(Win)
