# Supplementary material for: Discovery of a Series of 1,2,3-Triazole-Containing Erlotinib Derivatives With Potent Anti-Tumor Activities Against Non-Small Cell Lung Cancer
Source: Front Chem. 2022 Jan 7;9:789030. doi: 10.3389/fchem.2021.789030 (PMC8776995; doi:10.3389/fchem.2021.789030)
Supplement: Supplementary file 4 [file DataSheet8.zip › H460 Cell cycle-2/rpt_20200810 h460 e 12h_e4 4uM_002.fcs.pdf]

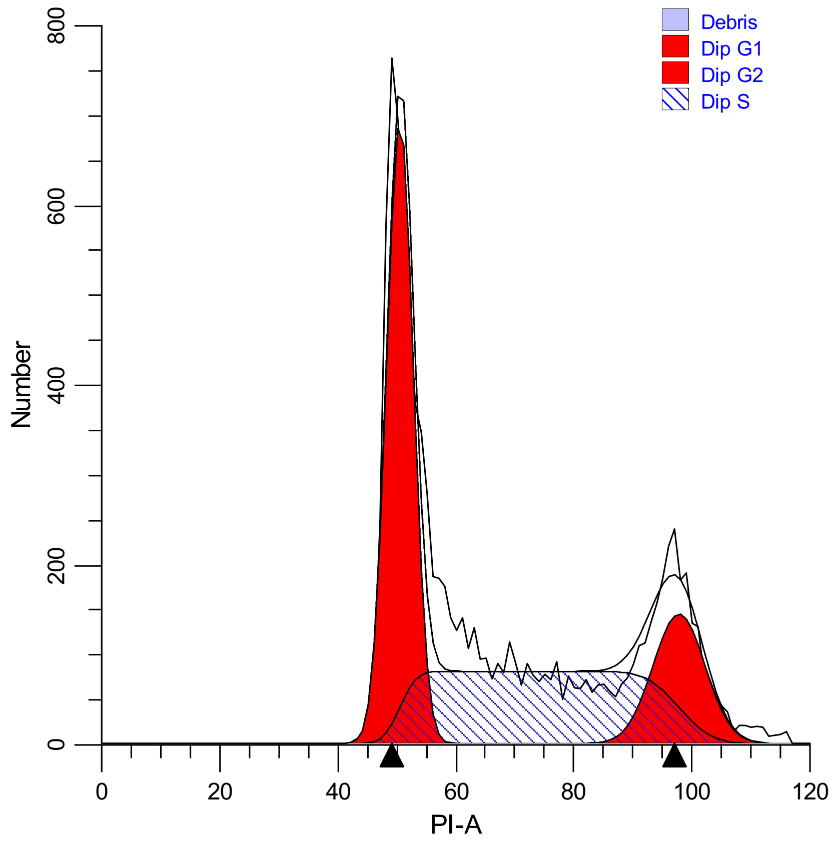

File analyzed: 20200810 h460 e 12h_e4 4uM_002.fcs
Date analyzed: 11-Aug-2020
Model: 1Dn0n_DSD
Analysis type: Manual analysis
Auto Linearity: No

Ploidy Mode: First cycle is diploid

Diploid: 100.00 %
Dip G1: 42.32 % at 50.36
Dip G2: 16.95 % at 97.70
Dip S: 40.73 % G2/G1: 1.94
%CV: 4.48

Total S-Phase: 40.73 %
Total B.A.D.: 0.00 % no aggs

Debris: 0.00 %
Aggregates: %
Modeled events: 9349
All cycle events: 9349
Cycle events per channel: 193
RCS: 6.306

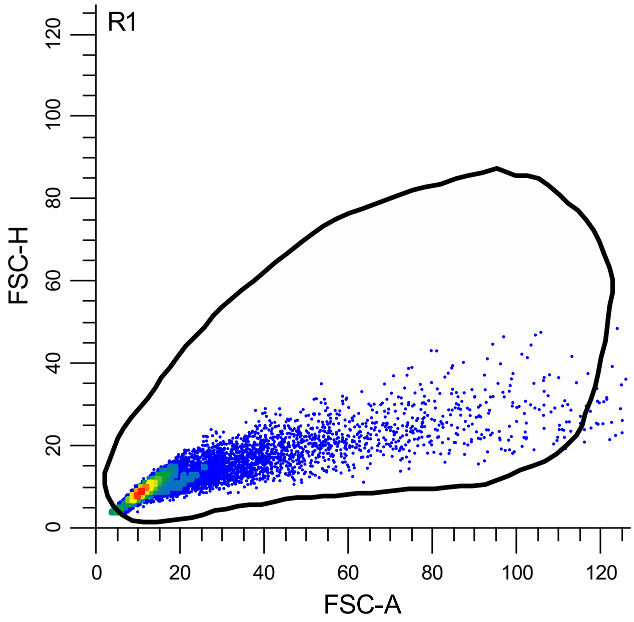

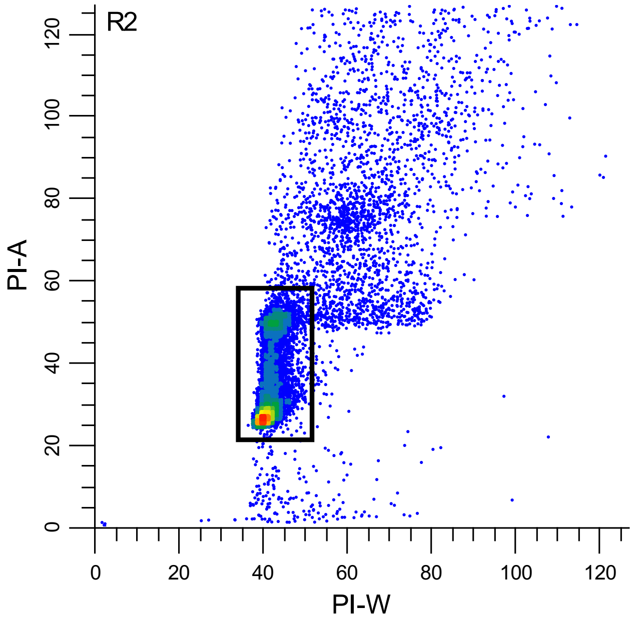

ModFit LT V5.0.9(Win)
