# Supplementary material for: Discovery of a Series of 1,2,3-Triazole-Containing Erlotinib Derivatives With Potent Anti-Tumor Activities Against Non-Small Cell Lung Cancer
Source: Front Chem. 2022 Jan 7;9:789030. doi: 10.3389/fchem.2021.789030 (PMC8776995; doi:10.3389/fchem.2021.789030)
Supplement: Supplementary file 5 [file DataSheet9.zip › H460 Cell cycle-3/rpt_20200812 h460 e 12h_001_e12 4uM_005.fcs.pdf]

File analyzed: 20200812 h460 e 12h_001_e12 4uM_005.fcs
Date analyzed: 27-Aug-2020
Model: 1Dn0n_DSD
Analysis type: Manual analysis
Auto Linearity: No

Ploidy Mode: First cycle is diploid

Diploid: 100.00 %
Dip G1: 43.30 % at 49.88
Dip G2: 18.68 % at 96.77
Dip S: 38.02 %   G2/G1: 1.94
%CV: 3.27

Total S-Phase: 38.02 %
Total B.A.D.: 0.00 %   no aggs

Debris: 0.05 %
Aggregates: %
Modeled events: 9329
All cycle events: 9324
Cycle events per channel: 195
RCS: 5.135

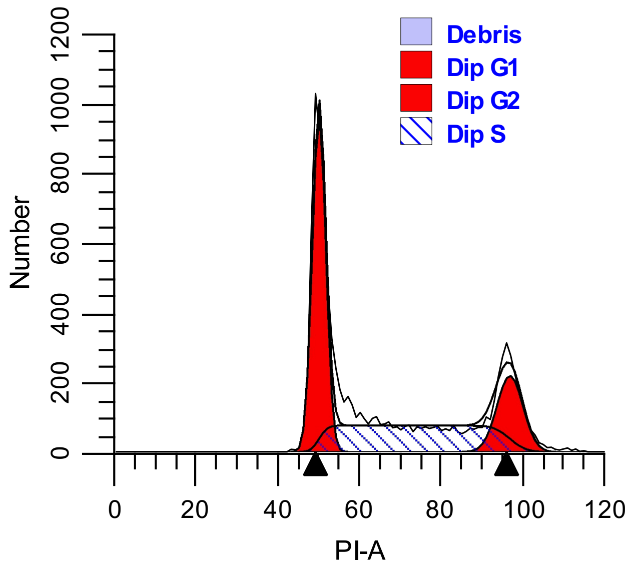

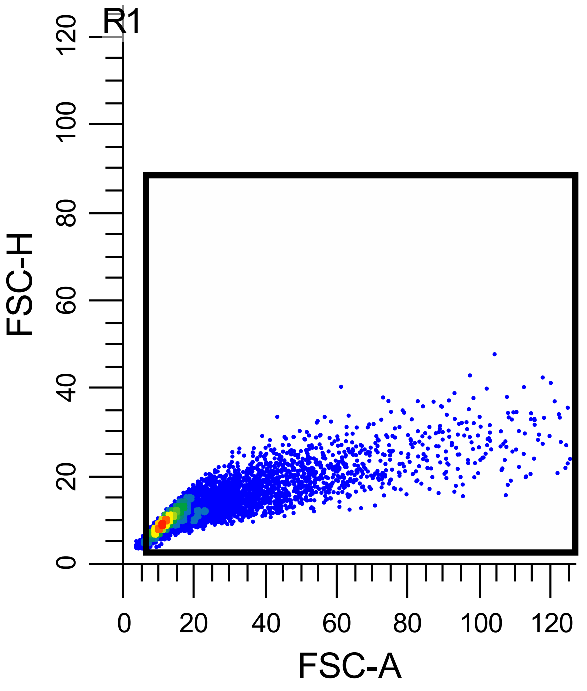

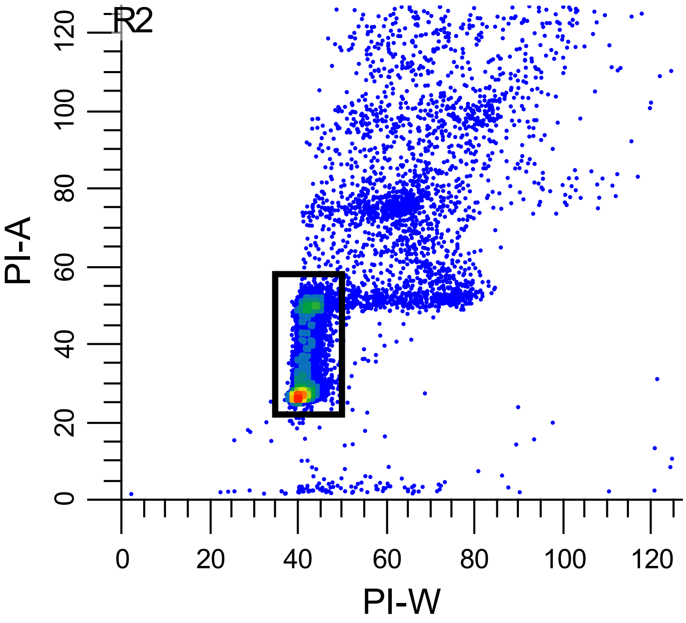

ModFit LT V5.0.9(Win)
