# Supplementary material for: Discovery of a Series of 1,2,3-Triazole-Containing Erlotinib Derivatives With Potent Anti-Tumor Activities Against Non-Small Cell Lung Cancer
Source: Front Chem. 2022 Jan 7;9:789030. doi: 10.3389/fchem.2021.789030 (PMC8776995; doi:10.3389/fchem.2021.789030)
Supplement: Supplementary file 5 [file DataSheet9.zip › H460 Cell cycle-3/rpt_20200812 h460 e 12h_001_e12 8uM_006.fcs.pdf]

File analyzed: 20200812 h460 e 12h_001_e12 8uM_006.fcs
Date analyzed: 27-Aug-2020
Model: 1Dn0n_DSD
Analysis type: Manual analysis
Auto Linearity: No

Ploidy Mode: First cycle is diploid

Diploid: 100.00 %
Dip G1: 46.32 % at 49.83
Dip G2: 18.32 % at 96.67
Dip S: 35.36 %   G2/G1: 1.94
%CV: 3.21

Total S-Phase: 35.36 %
Total B.A.D.: 0.00 %   no aggs

Debris: 0.01 %
Aggregates: %
Modeled events: 9381
All cycle events: 9380
Cycle events per channel: 196
RCS: 5.119

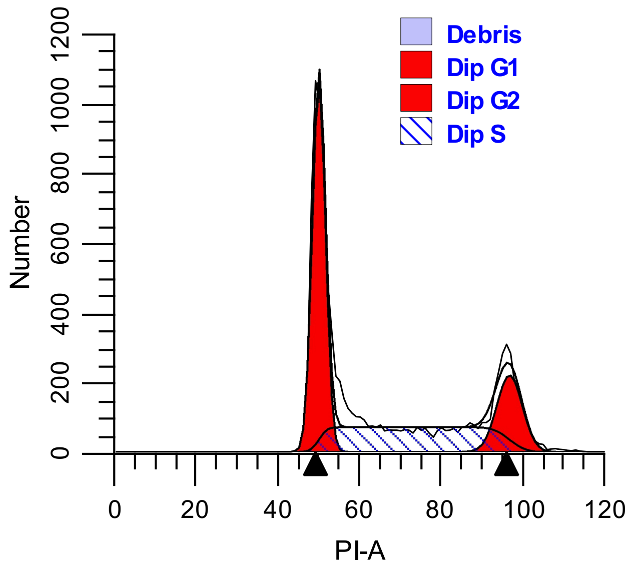

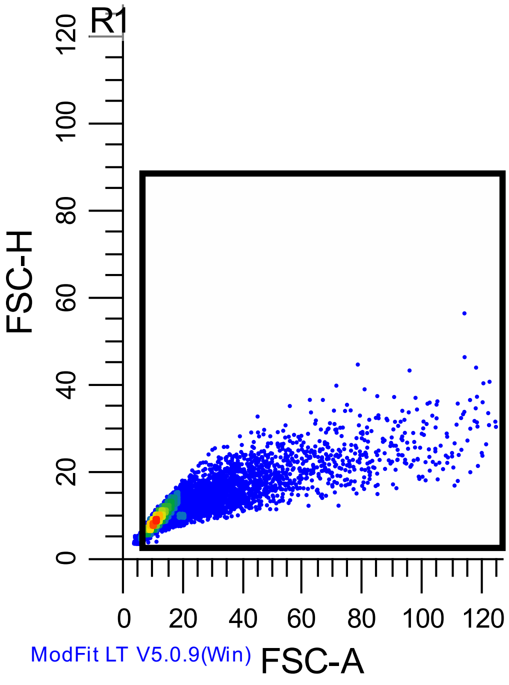

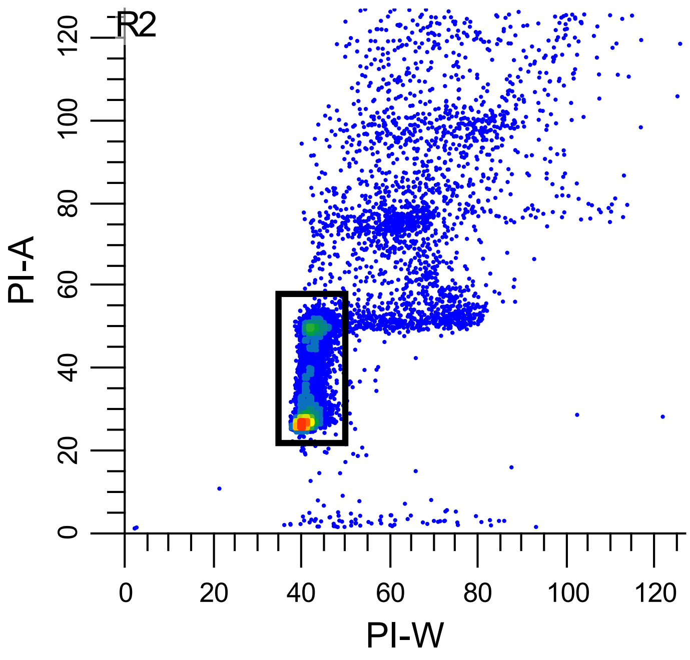

ModFit LT V5.0.9(Win)
