# Supplementary material for: Discovery of a Series of 1,2,3-Triazole-Containing Erlotinib Derivatives With Potent Anti-Tumor Activities Against Non-Small Cell Lung Cancer
Source: Front Chem. 2022 Jan 7;9:789030. doi: 10.3389/fchem.2021.789030 (PMC8776995; doi:10.3389/fchem.2021.789030)
Supplement: Supplementary file 5 [file DataSheet9.zip › H460 Cell cycle-3/rpt_20200812 h460 e 12h_001_e4 8uM_003.fcs.pdf]

File analyzed: 20200812 h460 e 12h_001_e4 8uM_003.fcs
Date analyzed: 27-Aug-2020
Model: 1Dn0n_DSD
Analysis type: Manual analysis
Auto Linearity: No

Ploidy Mode: First cycle is diploid

Diploid: 100.00 %
Dip G1: 44.75 % at 50.80
Dip G2: 18.81 % at 98.55
Dip S: 36.45 % G2/G1: 1.94
%CV: 3.01

Total S-Phase: 36.45 %
Total B.A.D.: 0.00 % no aggs

Debris: 0.06 %
Aggregates: %
Modeled events: 9405
All cycle events: 9400
Cycle events per channel: 193
RCS: 4.837

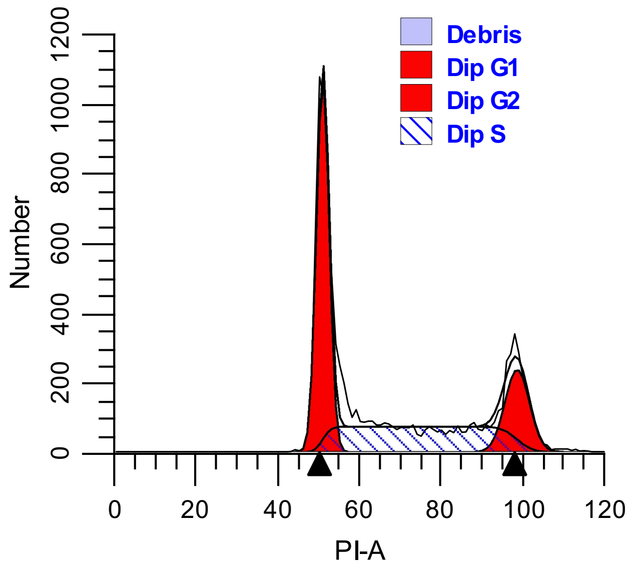


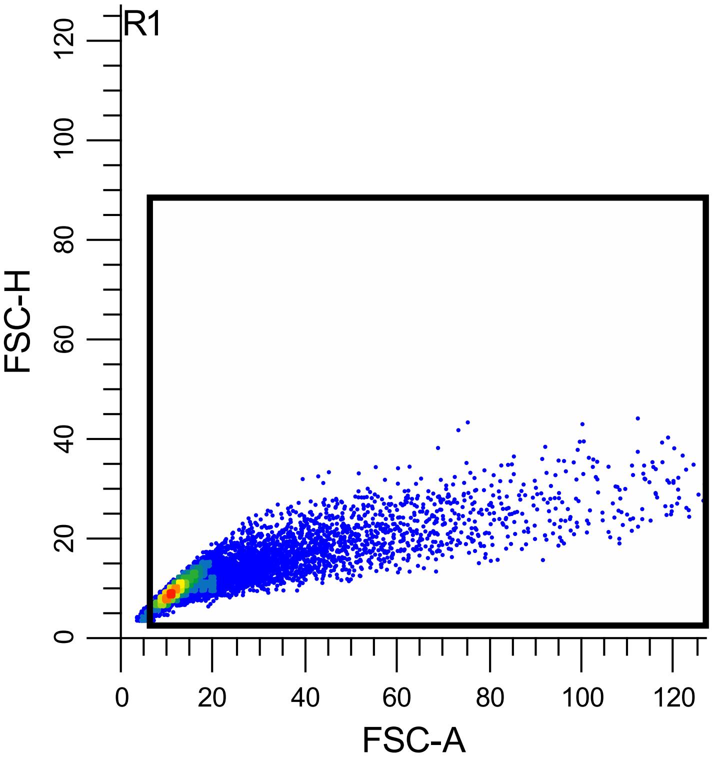


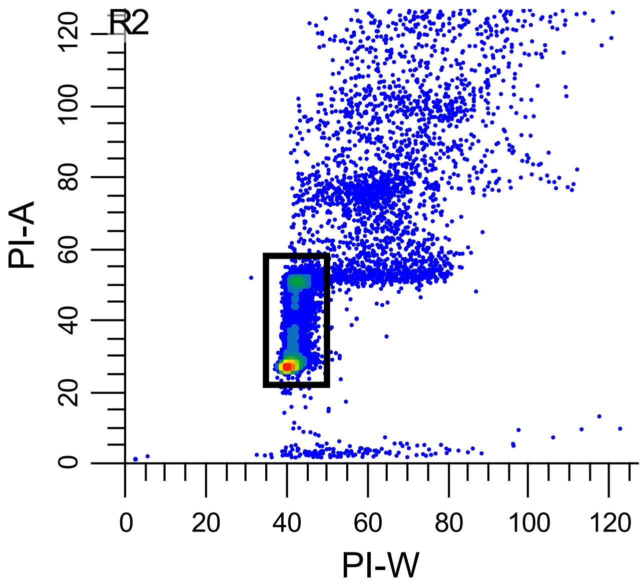


ModFit LT V5.0.9(Win)
