# Supplementary material for: Discovery of a Series of 1,2,3-Triazole-Containing Erlotinib Derivatives With Potent Anti-Tumor Activities Against Non-Small Cell Lung Cancer
Source: Front Chem. 2022 Jan 7;9:789030. doi: 10.3389/fchem.2021.789030 (PMC8776995; doi:10.3389/fchem.2021.789030)
Supplement: Supplementary file 5 [file DataSheet9.zip › H460 Cell cycle-3/rpt_20200812 h460 e 12h_001_NC1_001.fcs.pdf]

File analyzed: 20200812 h460 e 12h_001_NC1_001.fcs
Date analyzed: 27-Aug-2020
Model: 1Dn0n_DSD
Analysis type: Manual analysis
Auto Linearity: No

Ploidy Mode: First cycle is diploid

Diploid: 100.00 %
Dip G1: 34.74 % at 49.71
Dip G2: 19.34 % at 96.44
Dip S: 45.92 % G2/G1: 1.94
%CV: 3.60

Total S-Phase: 45.92 %
Total B.A.D.: 0.00 % no aggs

Debris: 0.06 %
Aggregates: %
Modeled events: 9417
All cycle events: 9412
Cycle events per channel: 197
RCS: 5.382

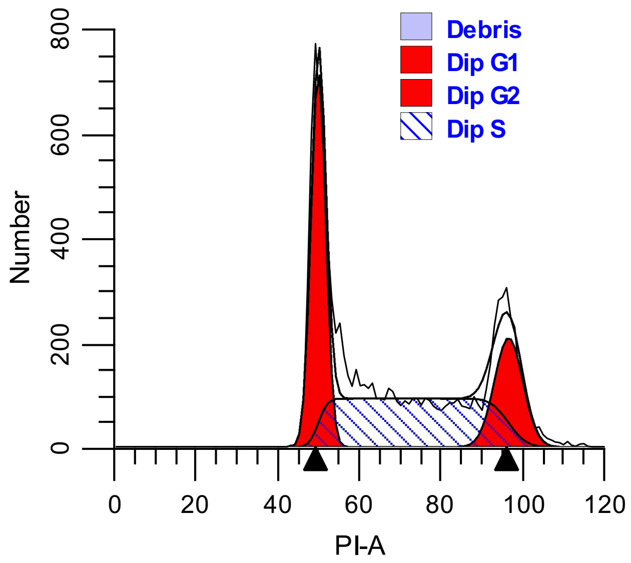

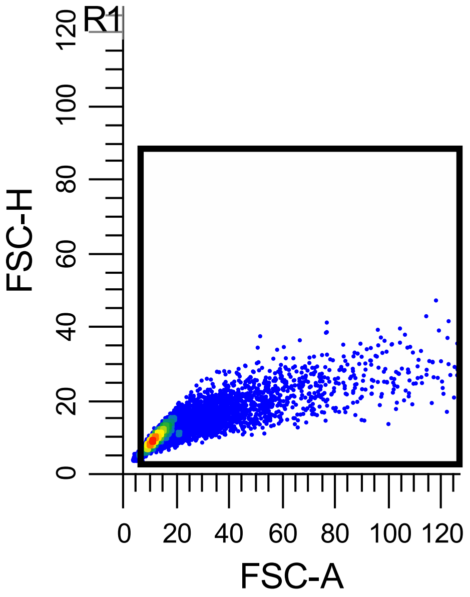

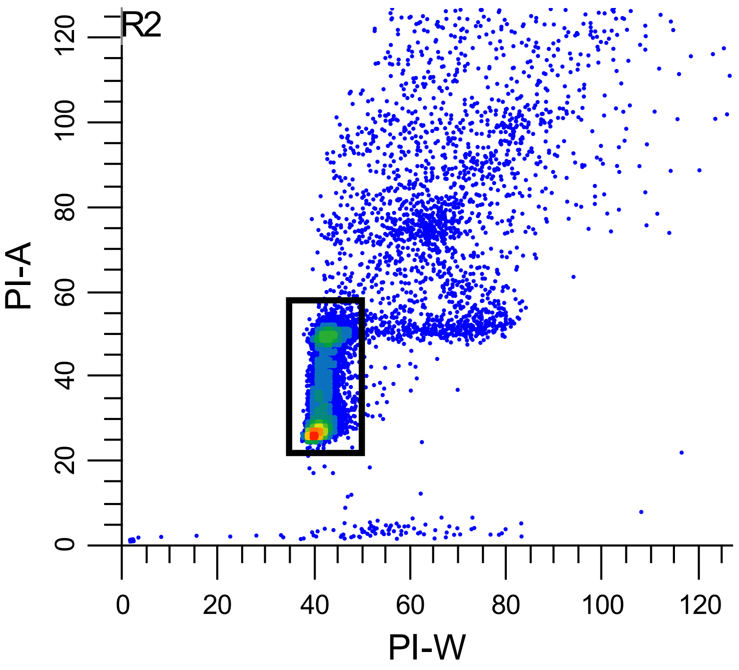

ModFit LT V5.0.9(Win)
