# Supplementary material for: Discovery of a Series of 1,2,3-Triazole-Containing Erlotinib Derivatives With Potent Anti-Tumor Activities Against Non-Small Cell Lung Cancer
Source: Front Chem. 2022 Jan 7;9:789030. doi: 10.3389/fchem.2021.789030 (PMC8776995; doi:10.3389/fchem.2021.789030)
Supplement: Supplementary file 8 [file DataSheet13.zip › 6. EGFR Kinase Assay/EGFR inhibitory results.docx]

| Compd no. | n | R^1^ | R^2^ | R^3^ | R^4^ | IC_50_ (μM)  EGFR |
| --- | --- | --- | --- | --- | --- | --- |
| e1 | 1 | H | H | H | H | 1.34 |
| e2 | 1 | I | H | H | H | 0.32 |
| e3 | 1 | Br | H | H | H | 68.02 |
| e4 | 1 | H | Br | H | Br | 13.01 |
| e5 | 1 | H | OCH_3_ | H | H | 35.16 |
| e6 | 0 | F | H | H | H | 0.69 |
| e7 | 0 | H | H | F | H | 14.74 |
| e8 | 0 | Cl | H | H | H | 1.99 |
| e9 | 0 | Br | H | H | H | 3.83 |
| e10 | 0 | H | H | Br | H | 2.13 |
| e11 | 0 | OCH_3_ | H | H | H | 49.39 |
| e12 | 0 | H | H | CH_3_ | H | 1.76 |
| e13 | 0 | H | NO_2_ | H | H | 4.74 |
| e14 | 0 | H | OCH_2_CH_3_ | H | H | 10.66 |
| e 15 | 0 | H | H | H | H | 0.09 |
| e16 | 0 | CF_3_ | H | H | H | 32.53 |
| e17 | 0 | OCH_3_ | H | OCH_3_ | H | 15.49 |
| e18 | 0 | OH | H | CH_3_ | H | 12.38 |
| e19 | 0 | H | OCH_3_ | OCH_3_ | H | 28.08 |
| e20 | 2 | H | H | H | H | 0.28 |
| Erlotinib |  |  |  |  |  | 0.0048 |
