# Supplementary material for: Discovery of a Series of 1,2,3-Triazole-Containing Erlotinib Derivatives With Potent Anti-Tumor Activities Against Non-Small Cell Lung Cancer
Source: Front Chem. 2022 Jan 7;9:789030. doi: 10.3389/fchem.2021.789030 (PMC8776995; doi:10.3389/fchem.2021.789030)
Supplement: Supplementary file 11 [file DataSheet3.PDF]

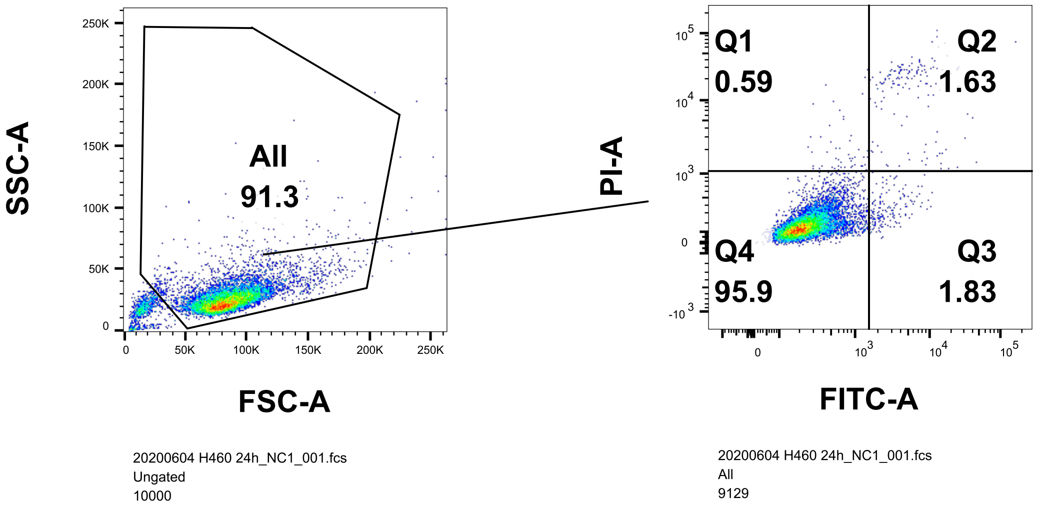
SSC-A
FSC-A
All
91.3
250K
200K
150K
100K
50K
0
0
50K
100K
150K
200K
250K
20200604 H460 24h_NC1_001.fcs
Ungated
10000
PI-A
FITC-A
Q1
0.59
Q2
1.63
Q3
1.83
Q4
95.9
20200604 H460 24h_NC1_001.fcs
All
9129

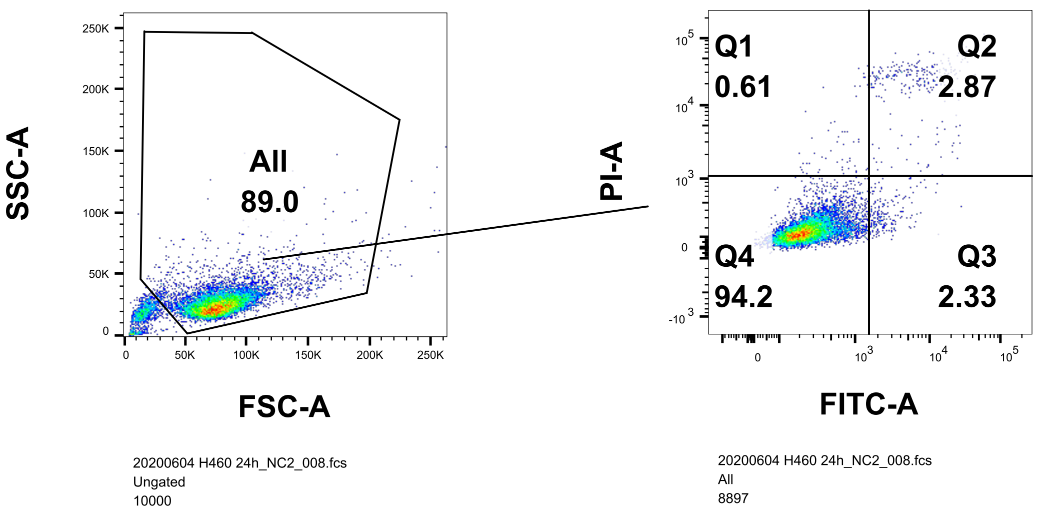

SSC-A
250K
200K
150K
100K
50K
0
All
89.0
0
50K
100K
150K
200K
250K
FSC-A
PI-A
Q1
0.61
Q2
2.87
Q4
94.2
Q3
2.33
FITC-A
20200604 H460 24h_NC2_008.fcs
Ungated
10000
20200604 H460 24h_NC2_008.fcs
All
8897

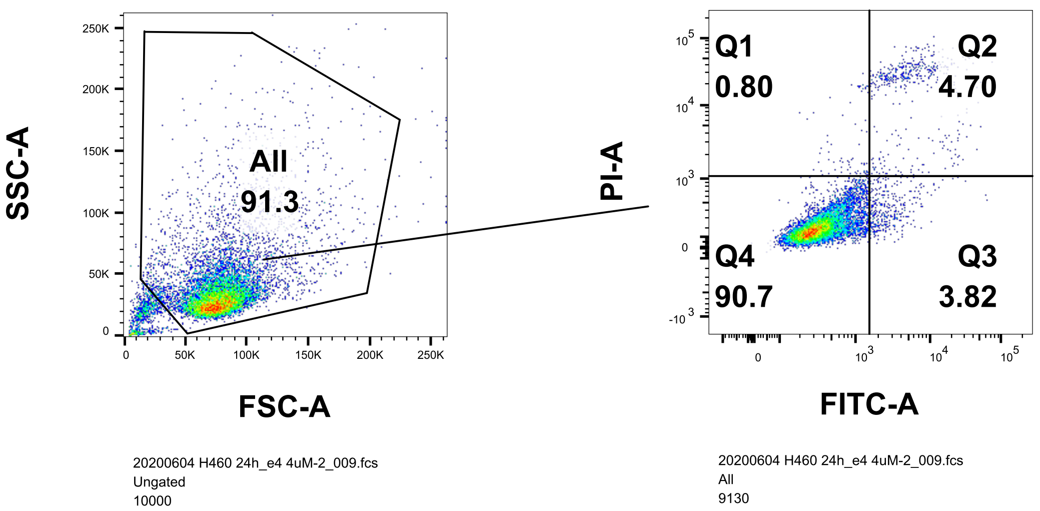
SSC-A
FSC-A
All
91.3
250K
200K
150K
100K
50K
0
50K
100K
150K
200K
250K
20200604 H460 24h_e4 4uM-2_009.fcs
Ungated
10000
PI-A
FITC-A
Q1
0.80
Q2
4.70
Q4
90.7
Q3
3.82
20200604 H460 24h_e4 4uM-2_009.fcs
All
9130

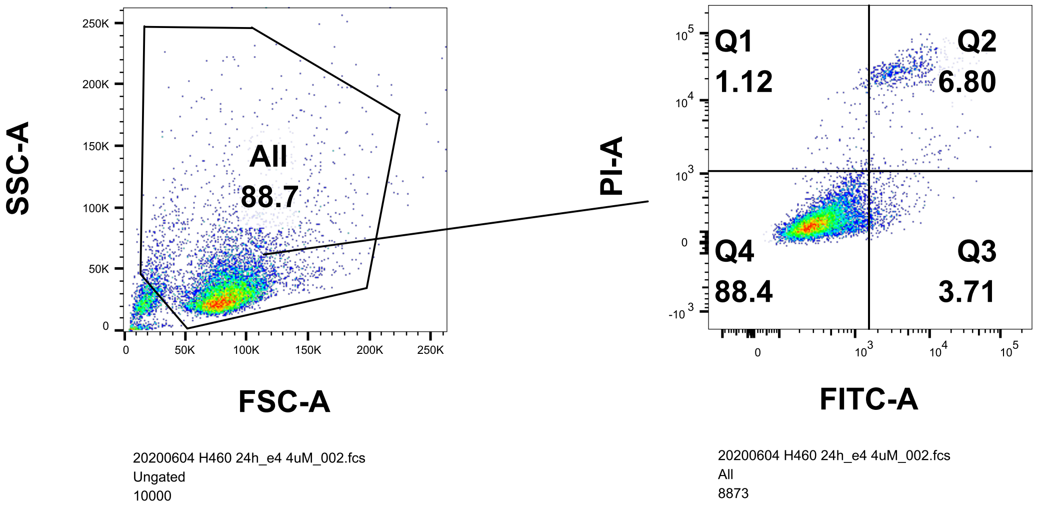
SSC-A
250K
200K
150K
100K
50K
0
All
88.7
0
50K
100K
150K
200K
250K
FSC-A
20200604 H460 24h_e4 4uM_002.fcs
Ungated
10000
PI-A
Q1
1.12
Q2
6.80
Q4
88.4
Q3
3.71
FITC-A
20200604 H460 24h_e4 4uM_002.fcs
All
8873

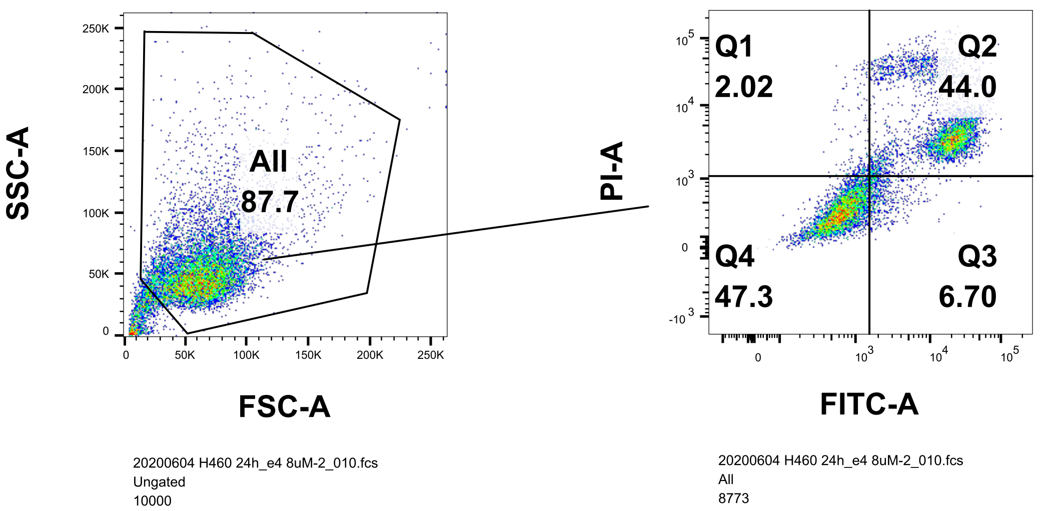
SSC-A
250K
200K
150K
100K
50K
0
All
87.7
0
50K
100K
150K
200K
250K
FSC-A
20200604 H460 24h_e4 8uM-2_010.fcs
Ungated
10000
PI-A
Q1
2.02
Q2
44.0
Q4
47.3
Q3
6.70
FITC-A
20200604 H460 24h_e4 8uM-2_010.fcs
All
8773

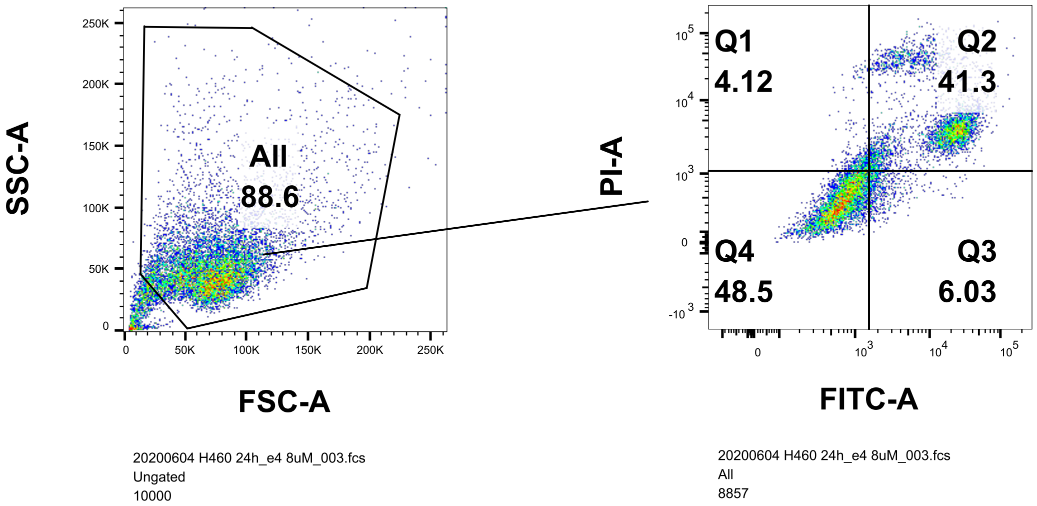

SSC-A
FSC-A
All
88.6
250K
200K
150K
100K
50K
0
50K
100K
150K
200K
250K
20200604 H460 24h_e4 8uM_003.fcs
Ungated
10000
PI-A
FITC-A
Q1
4.12
Q2
41.3
Q3
6.03
Q4
48.5
20200604 H460 24h_e4 8uM_003.fcs
All
8857

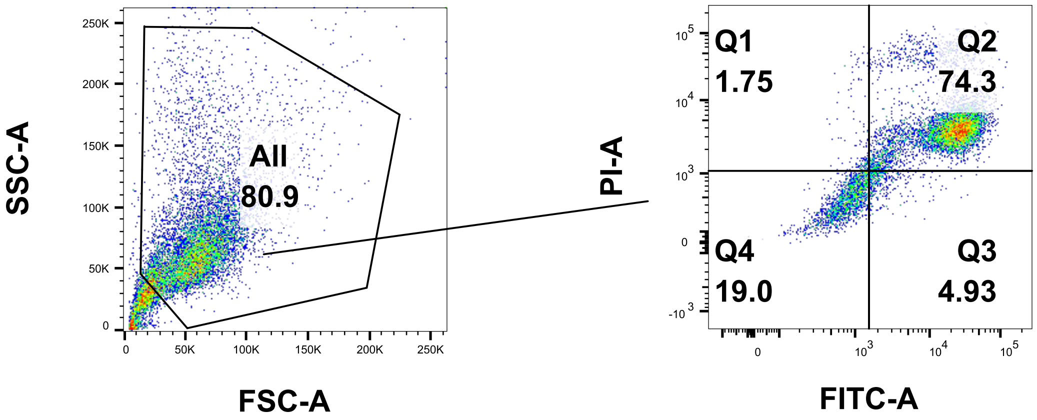

20200604 H460 24h_e4 12uM-2_011.fcs
Ungated
10000

20200604 H460 24h_e4 12uM-2_011.fcs
All
8093

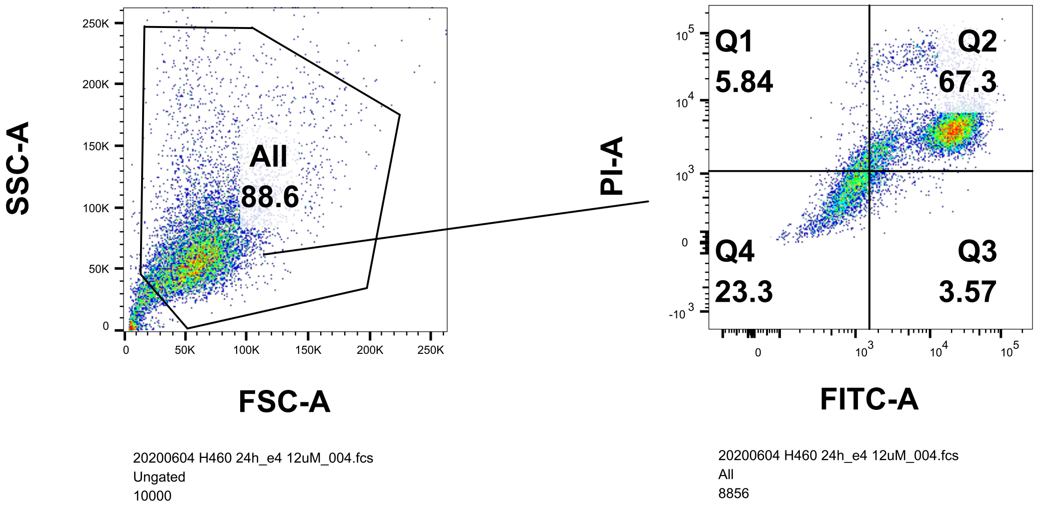
SSC-A
250K
200K
150K
100K
50K
0
0
50K
100K
150K
200K
250K
All
88.6
FSC-A
20200604 H460 24h_e4 12uM_004.fcs
Ungated
10000
PI-A
Q1
5.84
Q2
67.3
Q4
23.3
Q3
3.57
FITC-A
20200604 H460 24h_e4 12uM_004.fcs
All
8856
