# Supplementary material for: Discovery of a Series of 1,2,3-Triazole-Containing Erlotinib Derivatives With Potent Anti-Tumor Activities Against Non-Small Cell Lung Cancer
Source: Front Chem. 2022 Jan 7;9:789030. doi: 10.3389/fchem.2021.789030 (PMC8776995; doi:10.3389/fchem.2021.789030)

File analyzed: 20200916 PC-9 24H_e4 4uM_002.fcs
Date analyzed: 16-Sep-2020
Model: 1Dn0n_DSD
Analysis type: Manual analysis
Auto Linearity: No

Ploidy Mode: First cycle is diploid

Diploid: 100.00 %
Dip G1: 52.62 % at 57.53
Dip G2: 15.56 % at 110.45
Dip S: 31.83 %   G2/G1: 1.92
%CV: 2.41

Total S-Phase: 31.83 %
Total B.A.D.: 0.00 %   no aggs

Debris: 0.00 %
Aggregates: %
Modeled events: 9687
All cycle events: 9687
Cycle events per channel: 180
RCS: 2.468

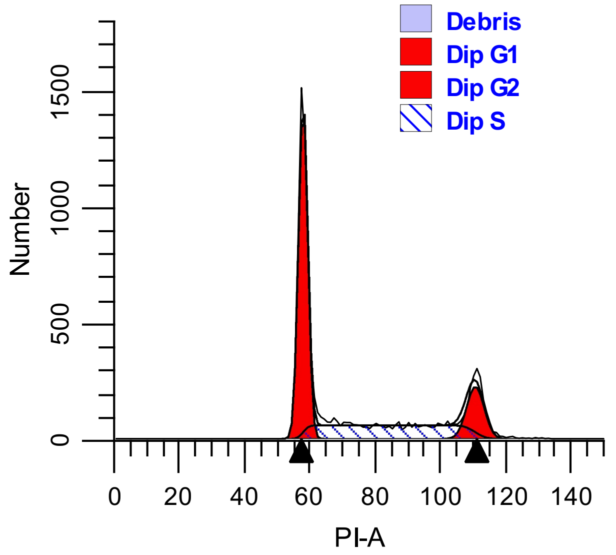

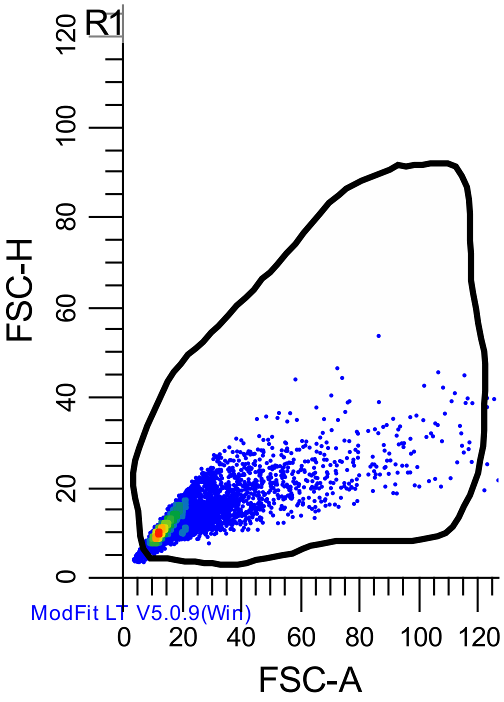

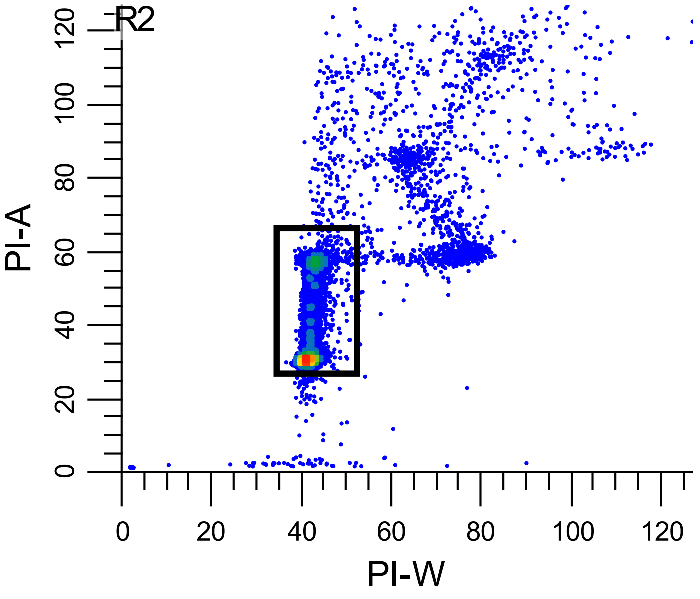

Supplement: Supplementary file 13 [file DataSheet10.zip › PC-9 Cell cycle-1/rpt_20200916 PC-9 24H_e4 4uM_002.fcs.pdf]
