# Supplementary material for: Discovery of a Series of 1,2,3-Triazole-Containing Erlotinib Derivatives With Potent Anti-Tumor Activities Against Non-Small Cell Lung Cancer
Source: Front Chem. 2022 Jan 7;9:789030. doi: 10.3389/fchem.2021.789030 (PMC8776995; doi:10.3389/fchem.2021.789030)

File analyzed: 20200916 PC-9 24H_e4 8uM_003.fcs
Date analyzed: 16-Sep-2020
Model: 1Dn0n_DSD
Analysis type: Manual analysis
Auto Linearity: No

Ploidy Mode: First cycle is diploid

Diploid: 100.00 %
Dip G1: 55.05 % at 58.90
Dip G2: 16.88 % at 114.27
Dip S: 28.08 % G2/G1: 1.94
%CV: 2.17

Total S-Phase: 28.08 %
Total B.A.D.: 0.00 % no aggs

Debris: 0.04 %
Aggregates: %
Modeled events: 9781
All cycle events: 9776
Cycle events per channel: 173
RCS: 2.011

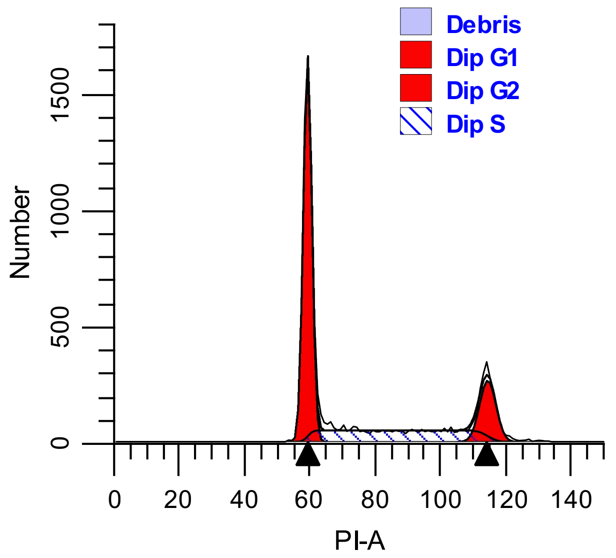

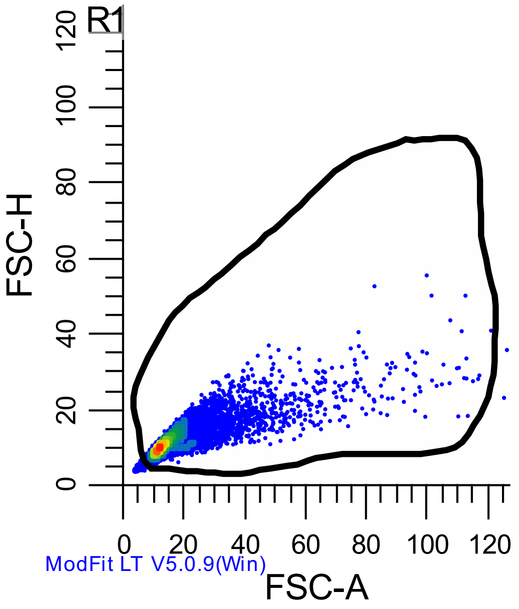

ModFit LT V5.0.9(Win)

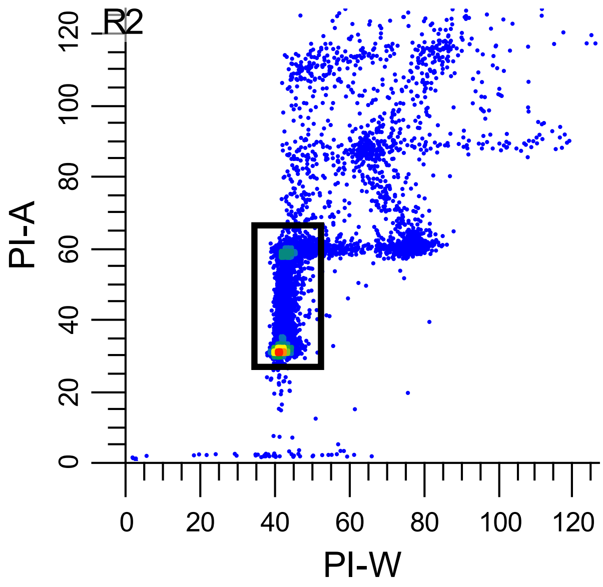

Supplement: Supplementary file 13 [file DataSheet10.zip › PC-9 Cell cycle-1/rpt_20200916 PC-9 24H_e4 8uM_003.fcs.pdf]
