# Supplementary material for: Discovery of a Series of 1,2,3-Triazole-Containing Erlotinib Derivatives With Potent Anti-Tumor Activities Against Non-Small Cell Lung Cancer
Source: Front Chem. 2022 Jan 7;9:789030. doi: 10.3389/fchem.2021.789030 (PMC8776995; doi:10.3389/fchem.2021.789030)
Supplement: Supplementary file 15 [file DataSheet1.pdf]

Supplementary Information

Discovery of a series of 1,2,3-triazole-containing Erlotinib derivatives with potent anti-tumor activities against non-small cell lung cancer (NSCLC)

Ge Sun[a,#], Longfei Mao[b,#], Wenjing Deng[a], Shuxiang Xu[a], Jie Zhao[b], Jianxue Yang[c,d], Kaitai Yao[a*], Miaomiao Yuan[e*] and Wei Li[b*]

[a]Cancer Research Institute, School of Basic Medical Sciences, Southern Medical University, Guangzhou 510515, Guangdong, China

[b]School of Chemistry and Chemical Engineering, Henan Normal University, Henan Engineering Research Center of Chiral Hydroxyl Pharmaceutical, Xinxiang 453007, China.

[c]Department of Neurology， The First Affiliated Hospital of Henan University of Science and Technology 450052, Henan, China.

[d]School of Nursing, Henan University of Science and Technology 450062, Henan, China

[e]The Eighth Affiliated Hospital, Sun Yat-sen University, Shenzhen 518033, Guangdong, China.

***Corresponding authors:**

Kaitai Yao, Southern Medical University, Guangzhou 510515, Guangdong, China. E-mail: ktyao1931@163.com

Miaomiao Yuan, The Eighth Affiliated Hospital, Sun Yat-sen University, Shenzhen

518033, Guangdong, China. E-mail: yuanmm2019@163.com

Wei Li, School of Chemistry and Chemical Engineering, Henan Normal University, Henan Engineering Research Center of Chiral Hydroxyl Pharmaceutical, Xinxiang 453007, China. E-mail: liweigq@163.com

Table of Contents

Figure S1-1. ^{1}H NMR spectrum (600 MHz, DMSO-d$_6$) of compound e1

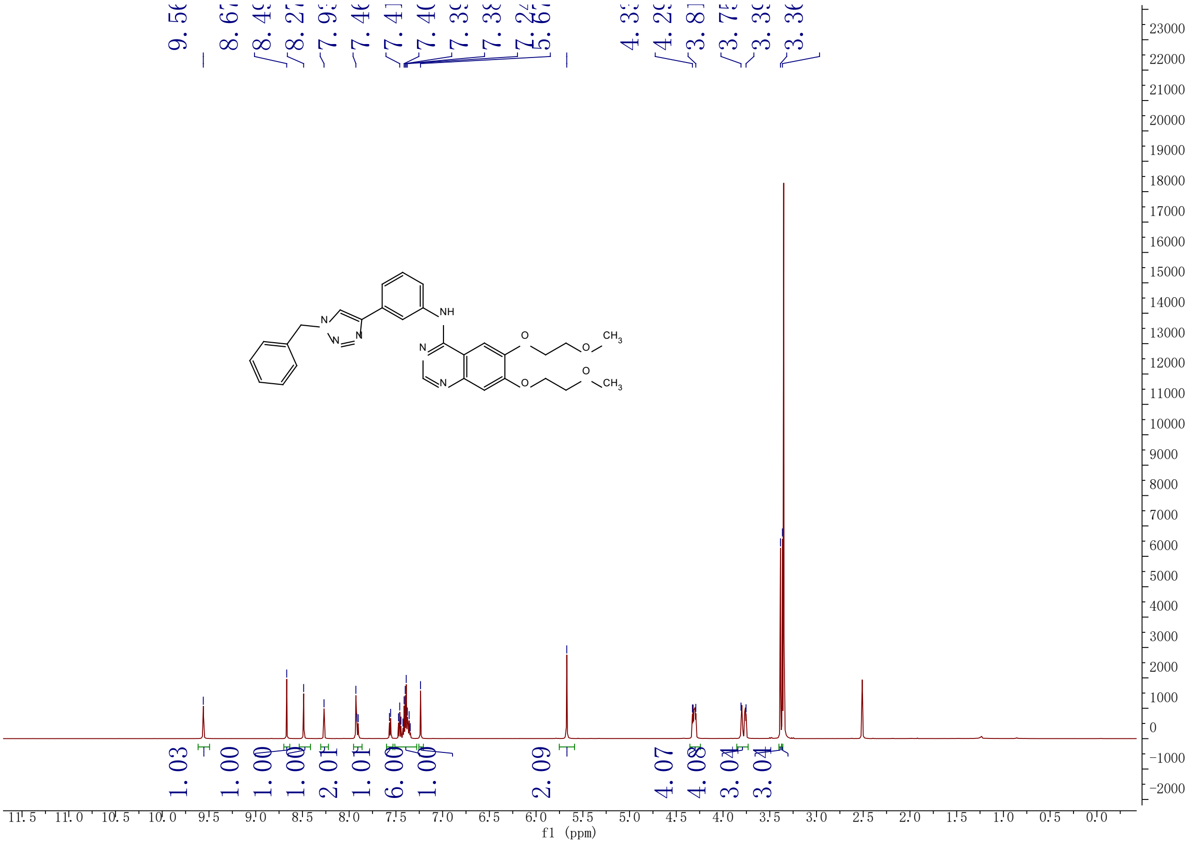

Figure S1-2. ^{13}C NMR spectrum (150 MHz, DMSO-d_6) of compound e1

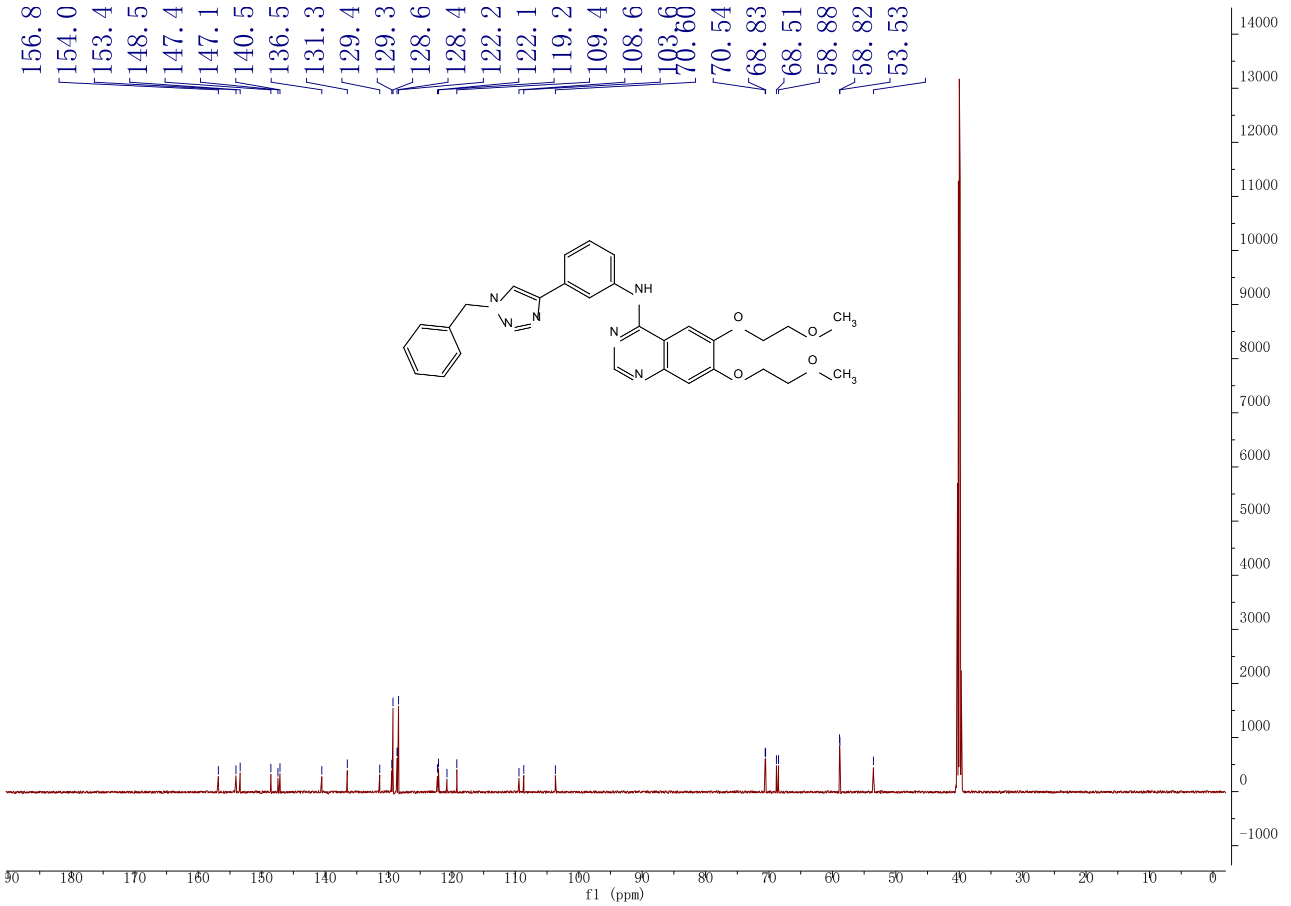

Figure S1-3. HR MS of compound e1

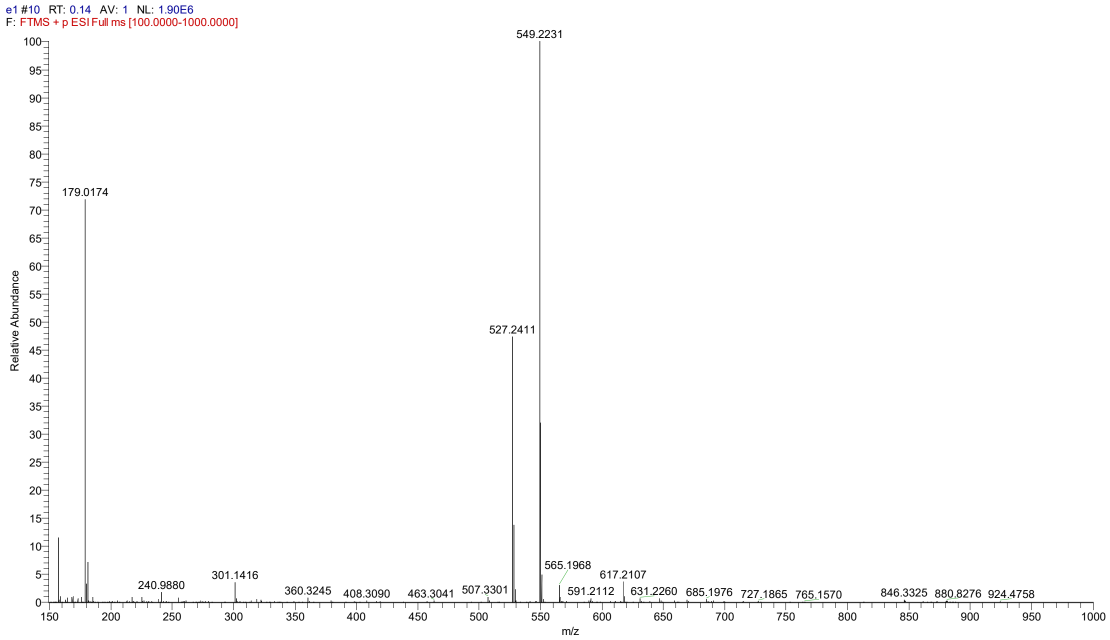

Figure S2-1. ^{1}H NMR spectrum (600 MHz, DMSO-d$_6$) of compound e2

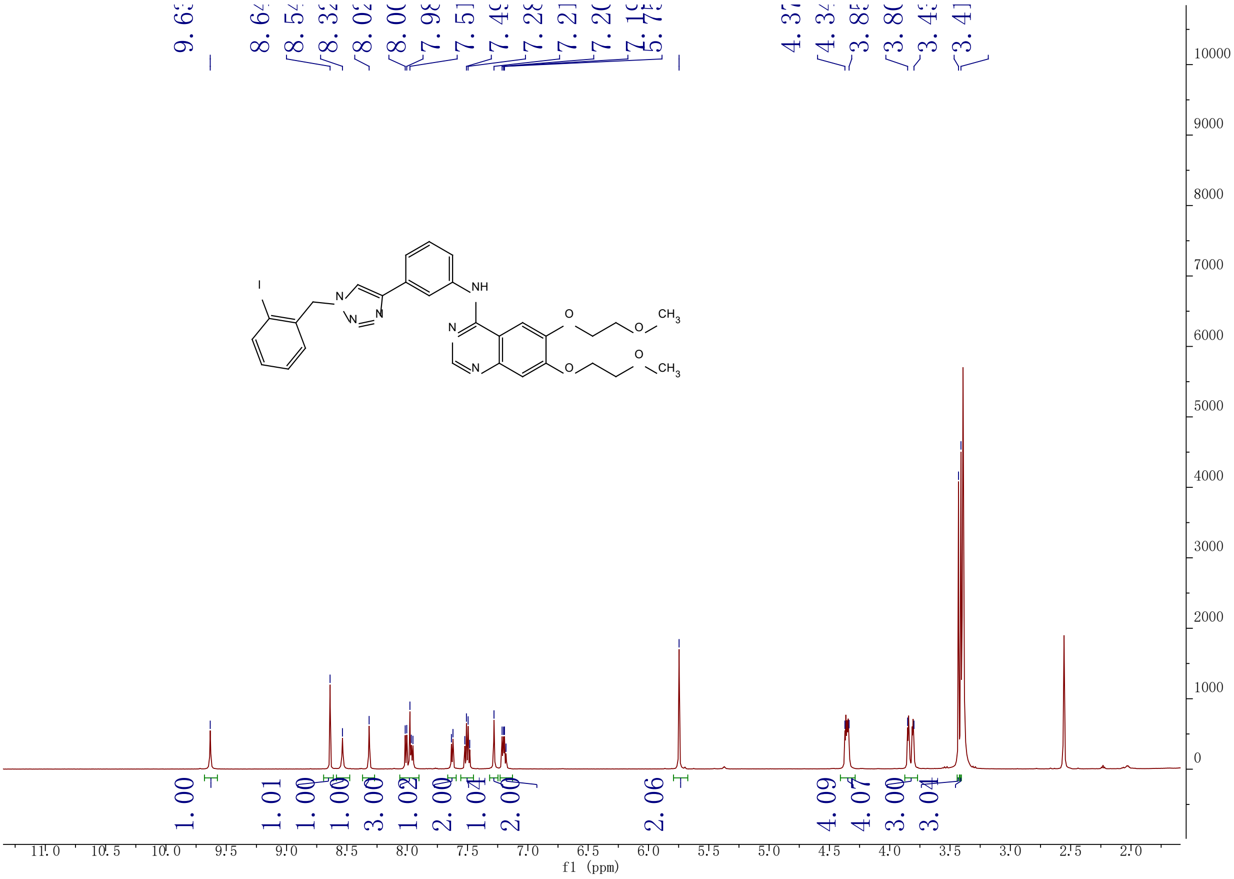

Figure S2-2. ^{13}C NMR spectrum (150 MHz, DMSO-d_6) of compound e2

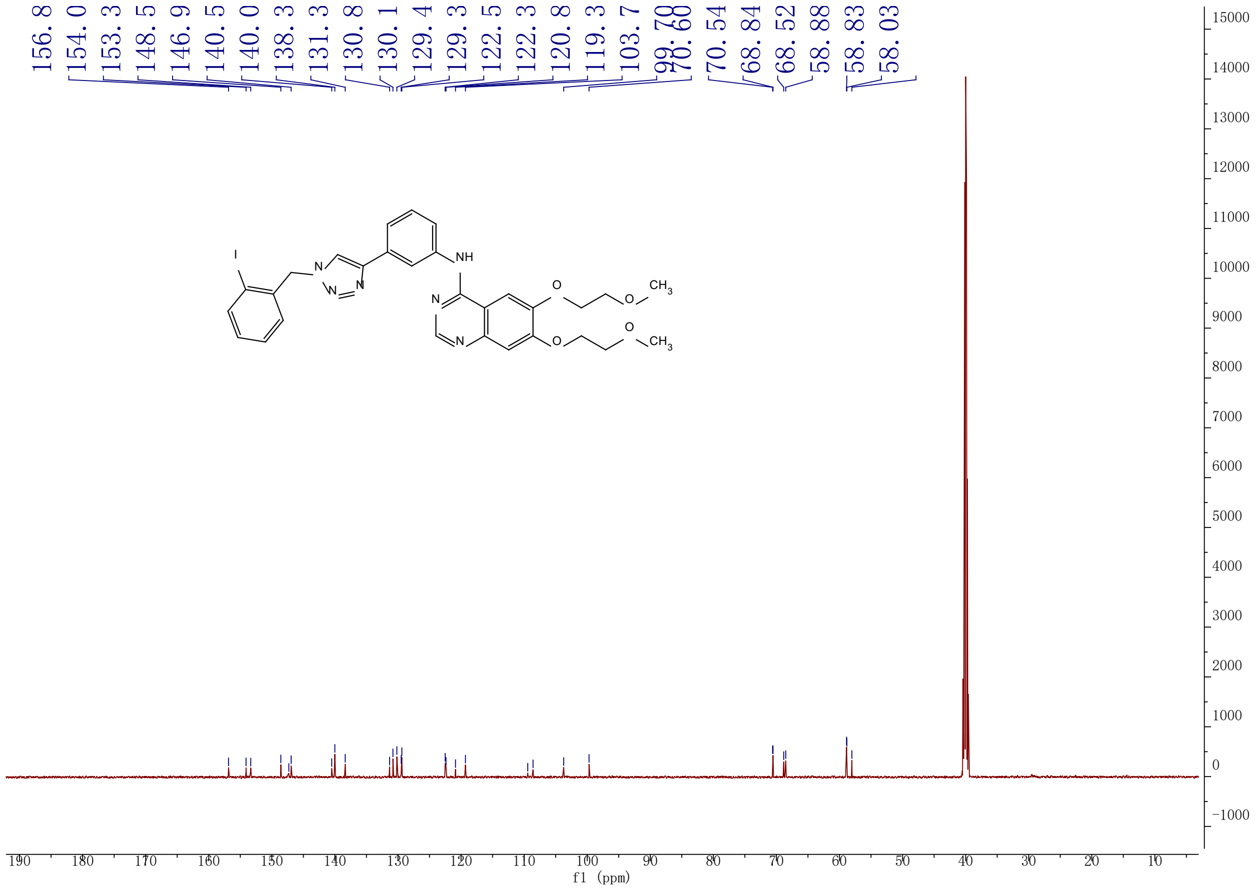

Figure S2-3. HR MS of compound e2

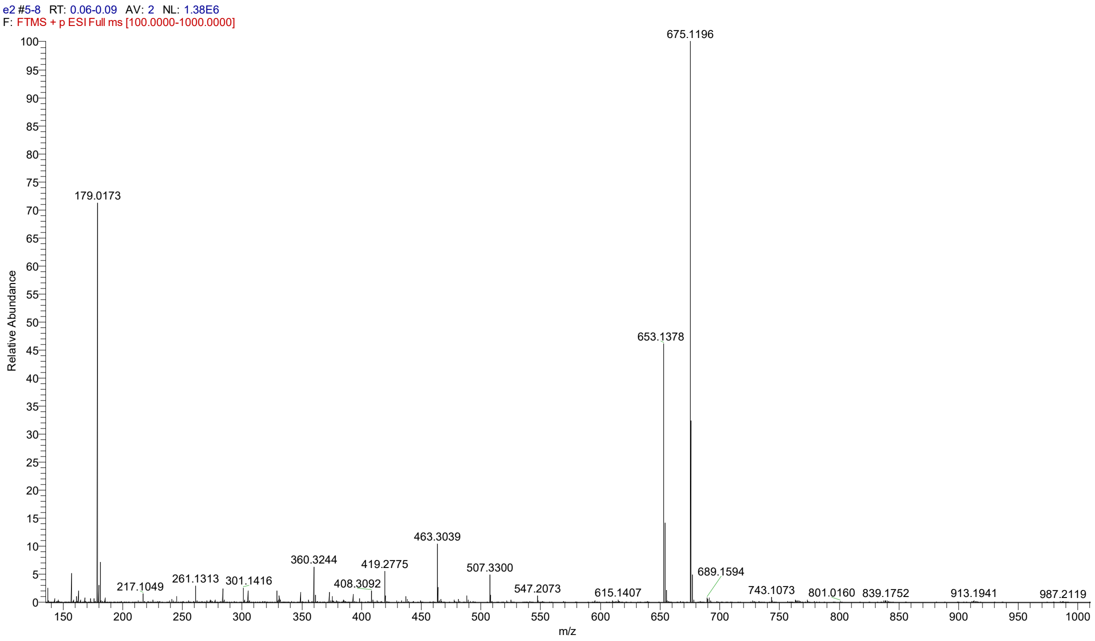

Figure S3-1. ^{1}H NMR spectrum (600 MHz, DMSO-d$_6$) of compound e3

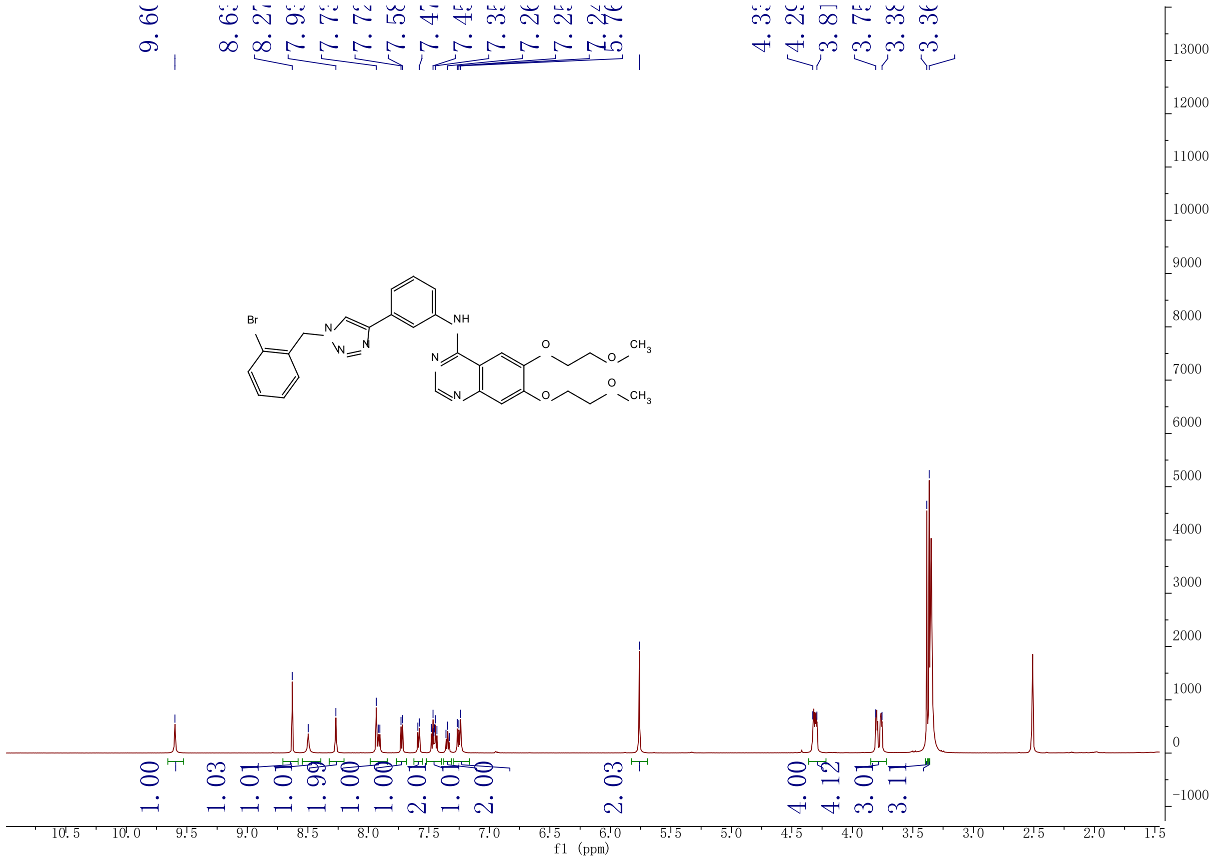

Figure S3-2. ^{13}C NMR spectrum (150 MHz, DMSO-d_6) of compound e3

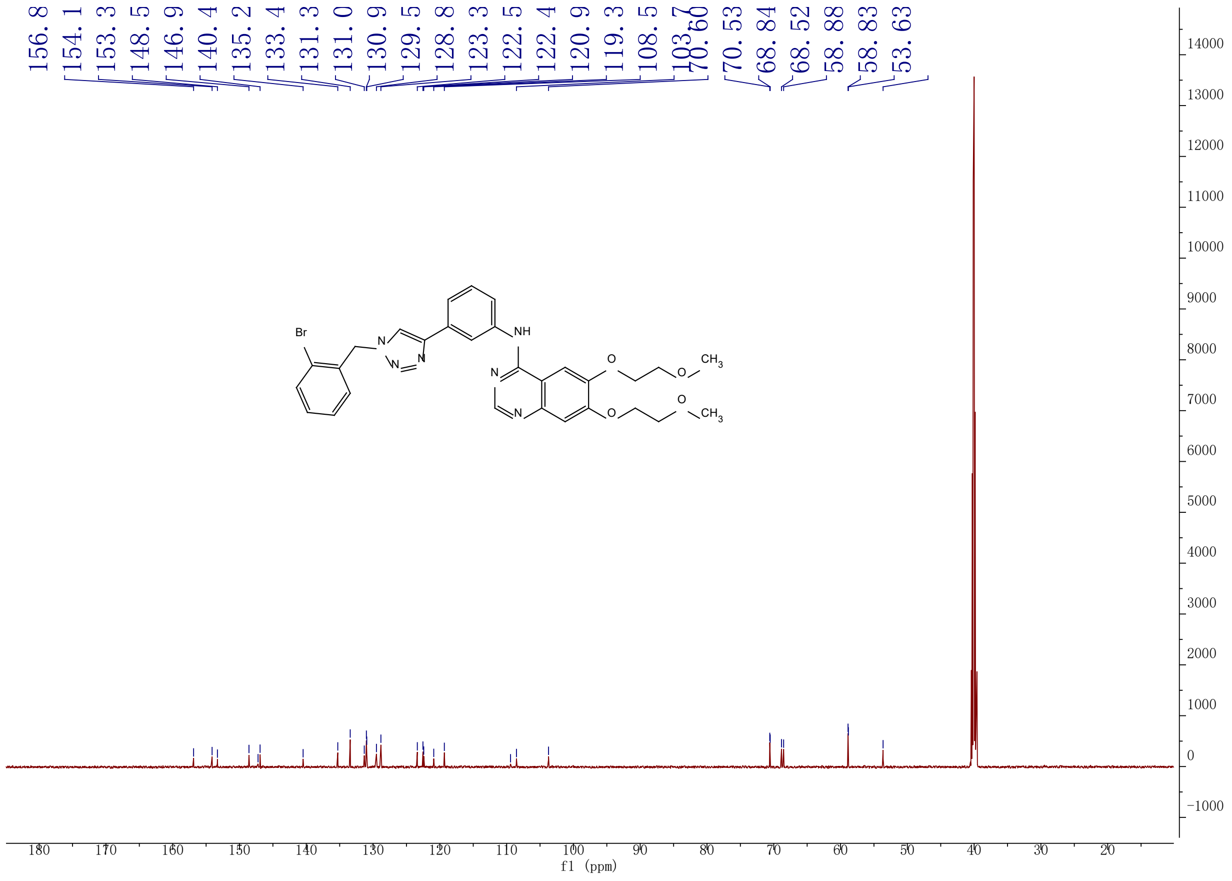

Figure S3-3. HR MS of compound e3

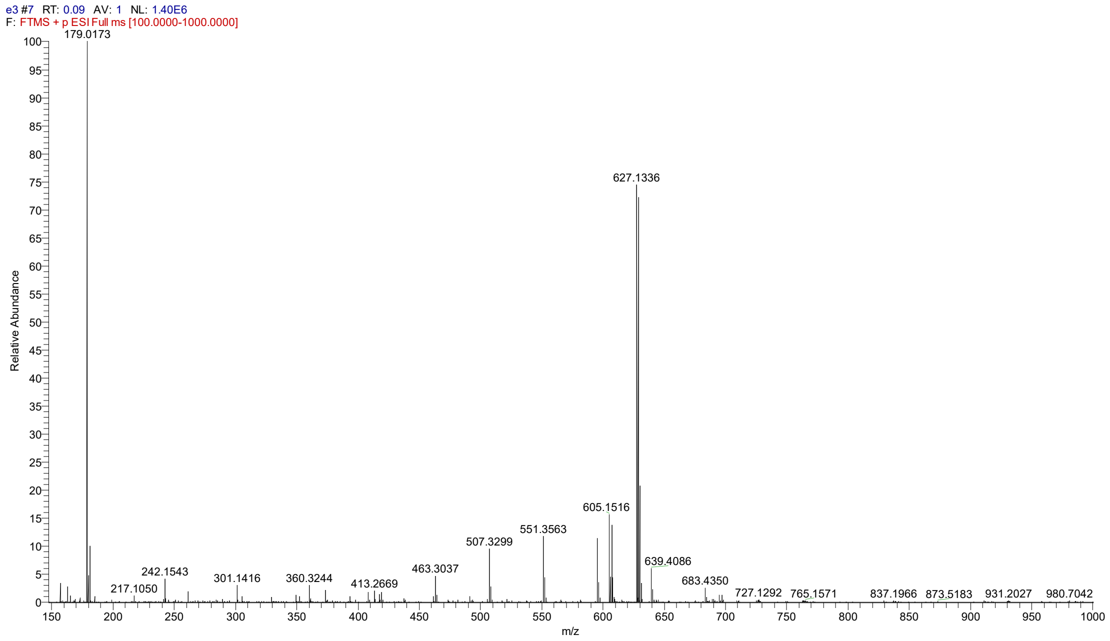

Figure S4-1. ^{1}H NMR spectrum (600 MHz, DMSO-d$_6$) of compound e4

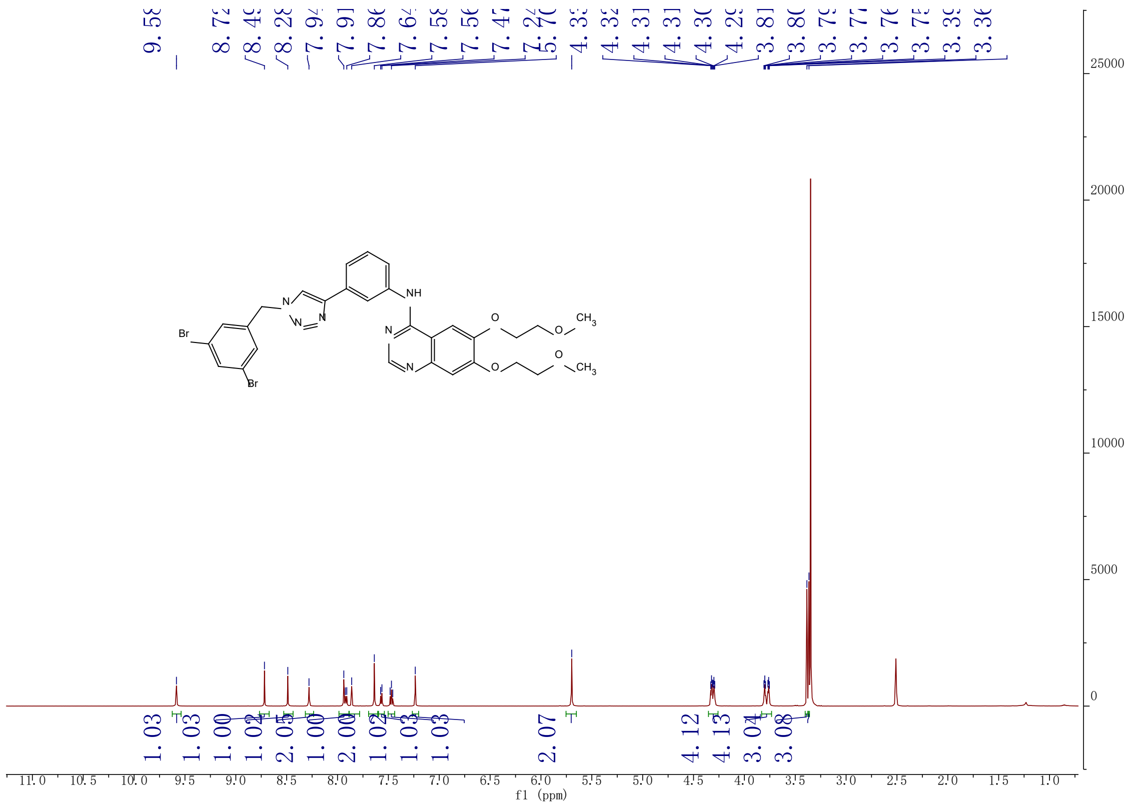

Figure S4-2. ^{13}C NMR spectrum (150 MHz, DMSO-d_6) of compound e4

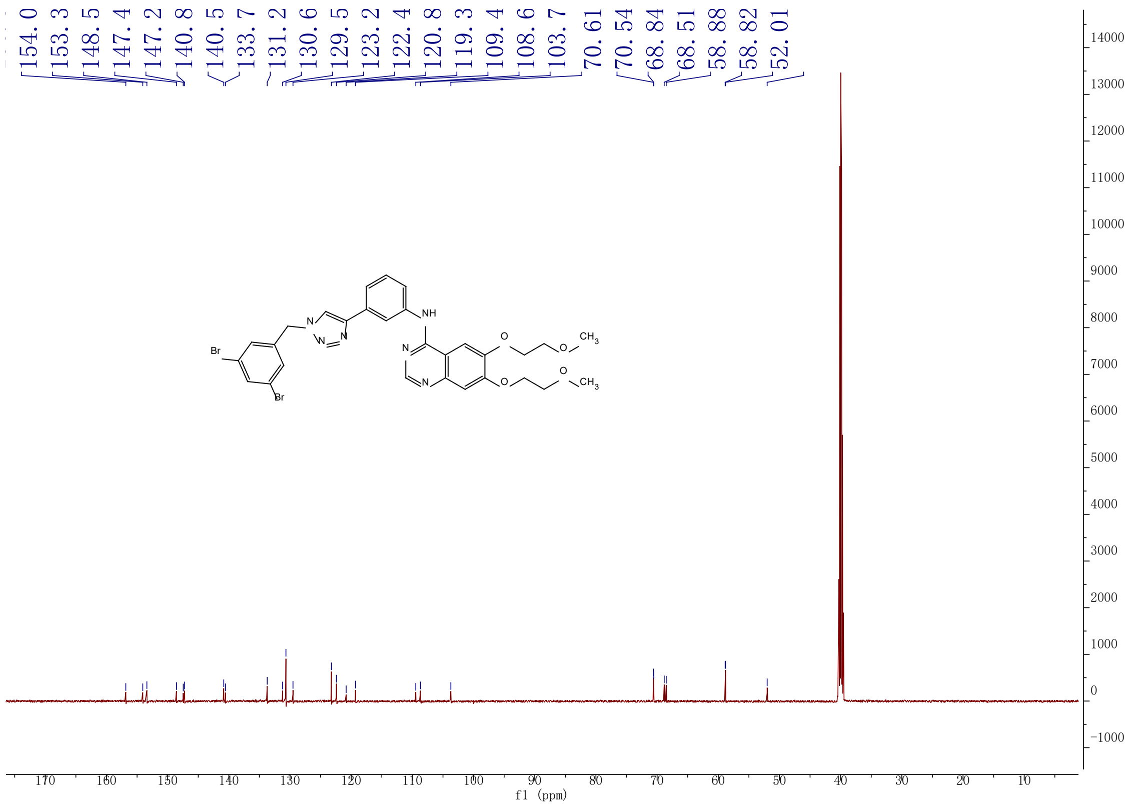

Figure S4-3. HR MS of compound e4

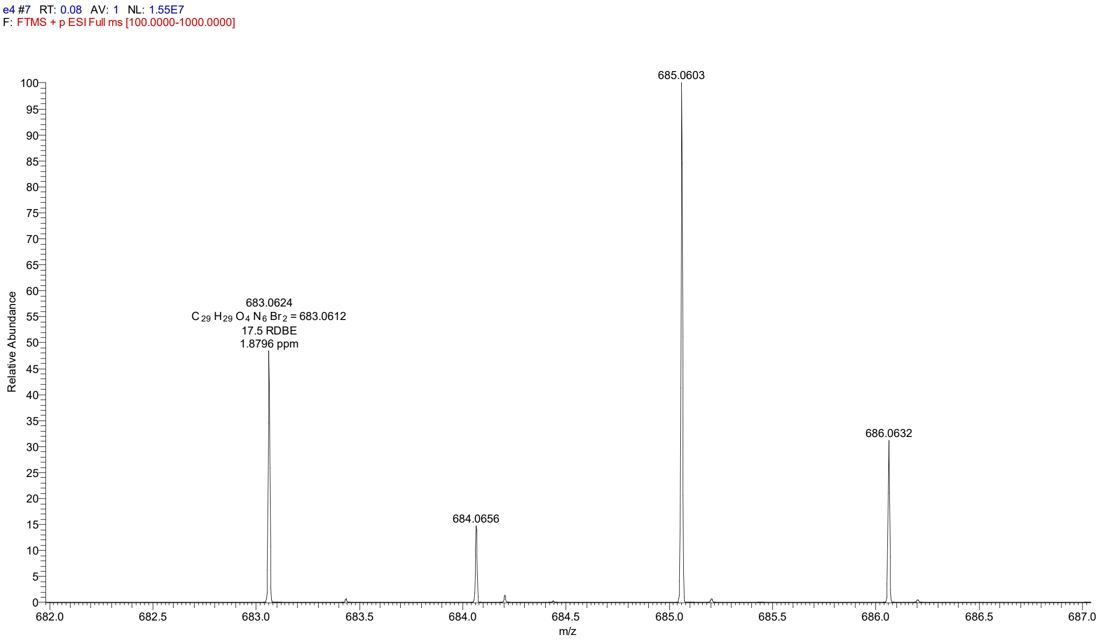

Figure S5-1. ^{1}H NMR spectrum (600 MHz, DMSO-d$_6$) of compound e5

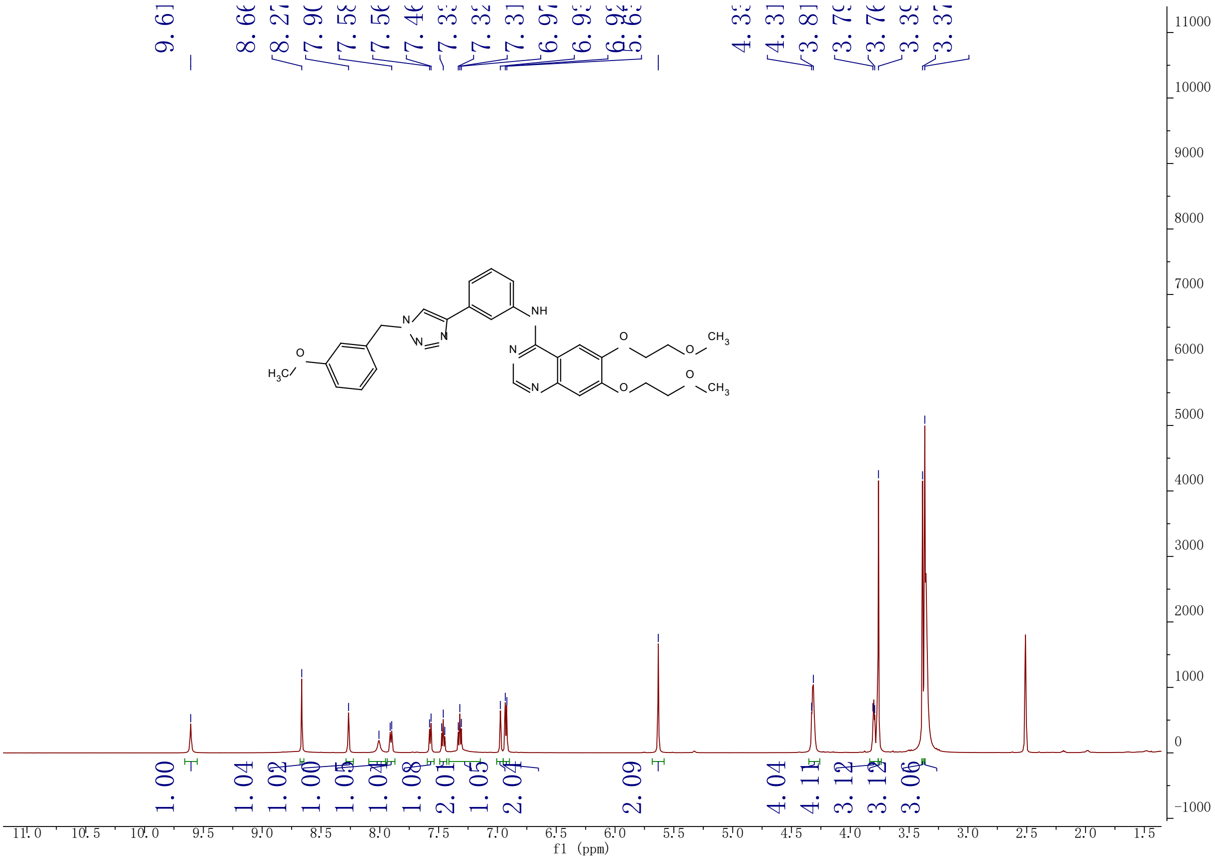

Figure S5-2. ^{13}C NMR spectrum (150 MHz, DMSO-d_6) of compound e5

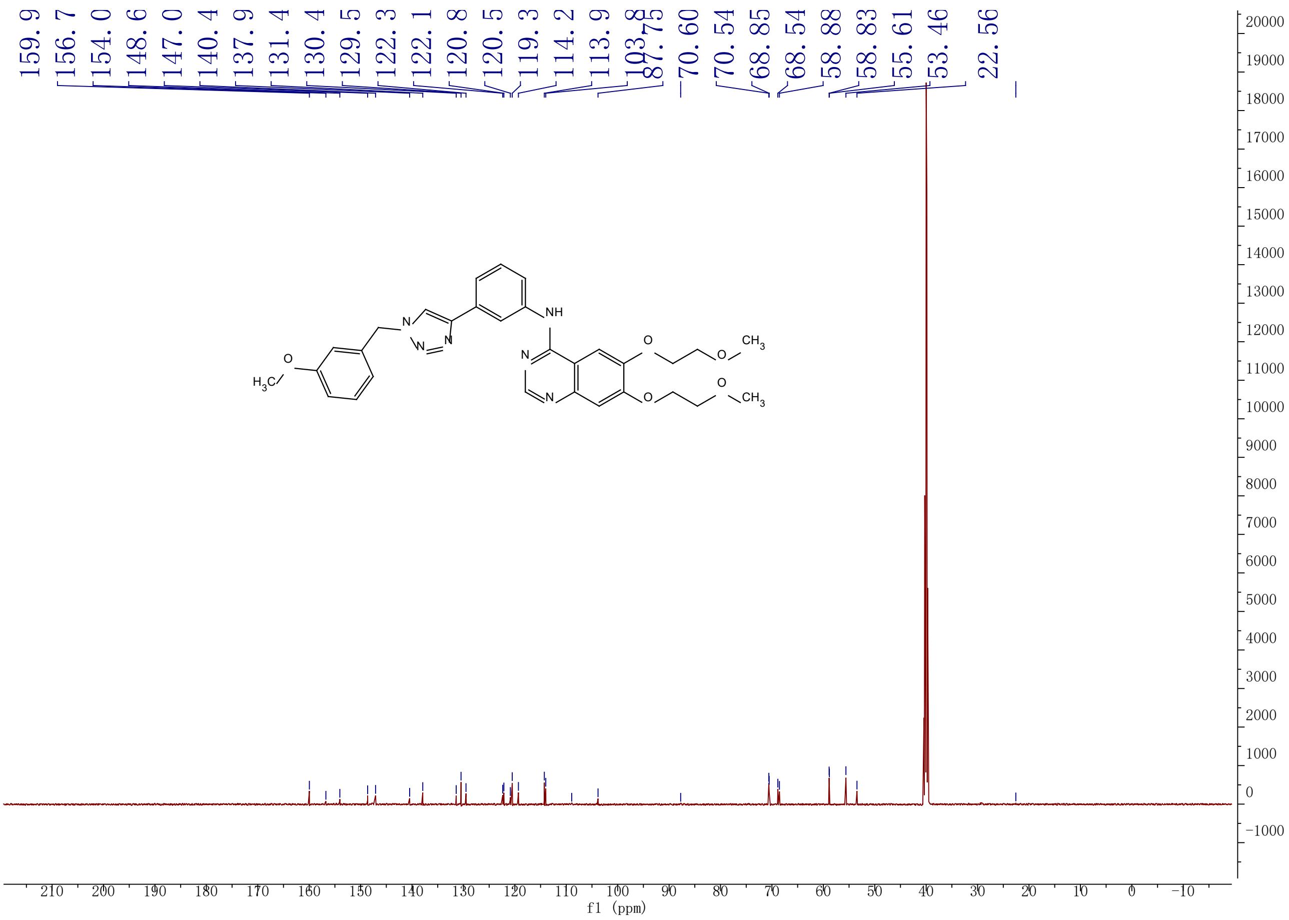

Figure S5-3. HR MS of compound e5

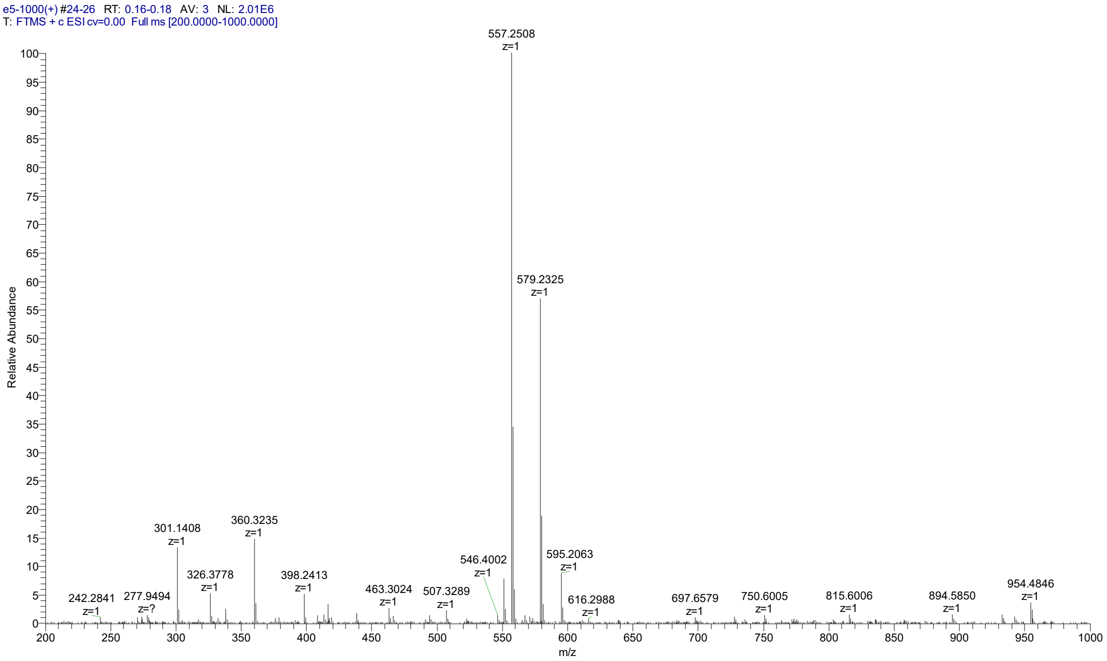

Figure S6-1. ^{1}H NMR spectrum (600 MHz, DMSO-d$_6$) of compound e6

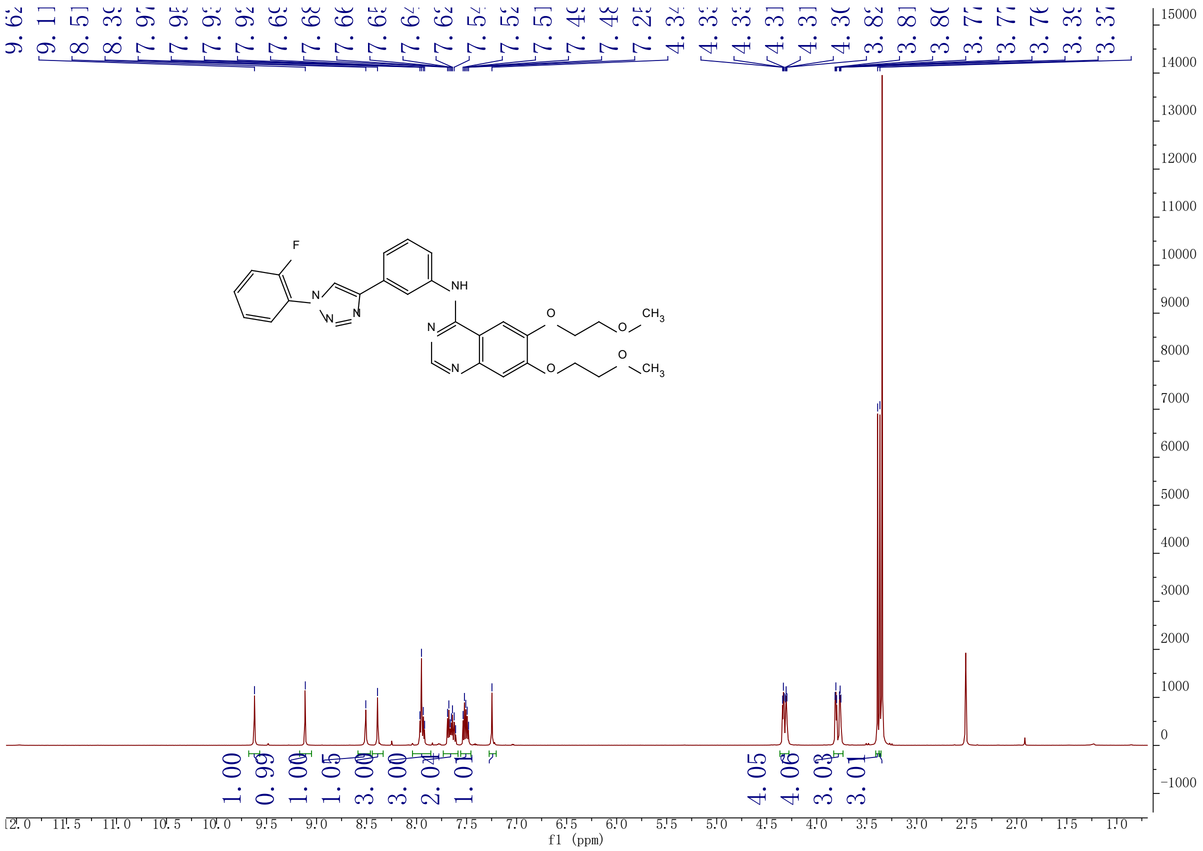

Figure S6-2. ^{13}C NMR spectrum (150 MHz, DMSO-d_6) of compound e6

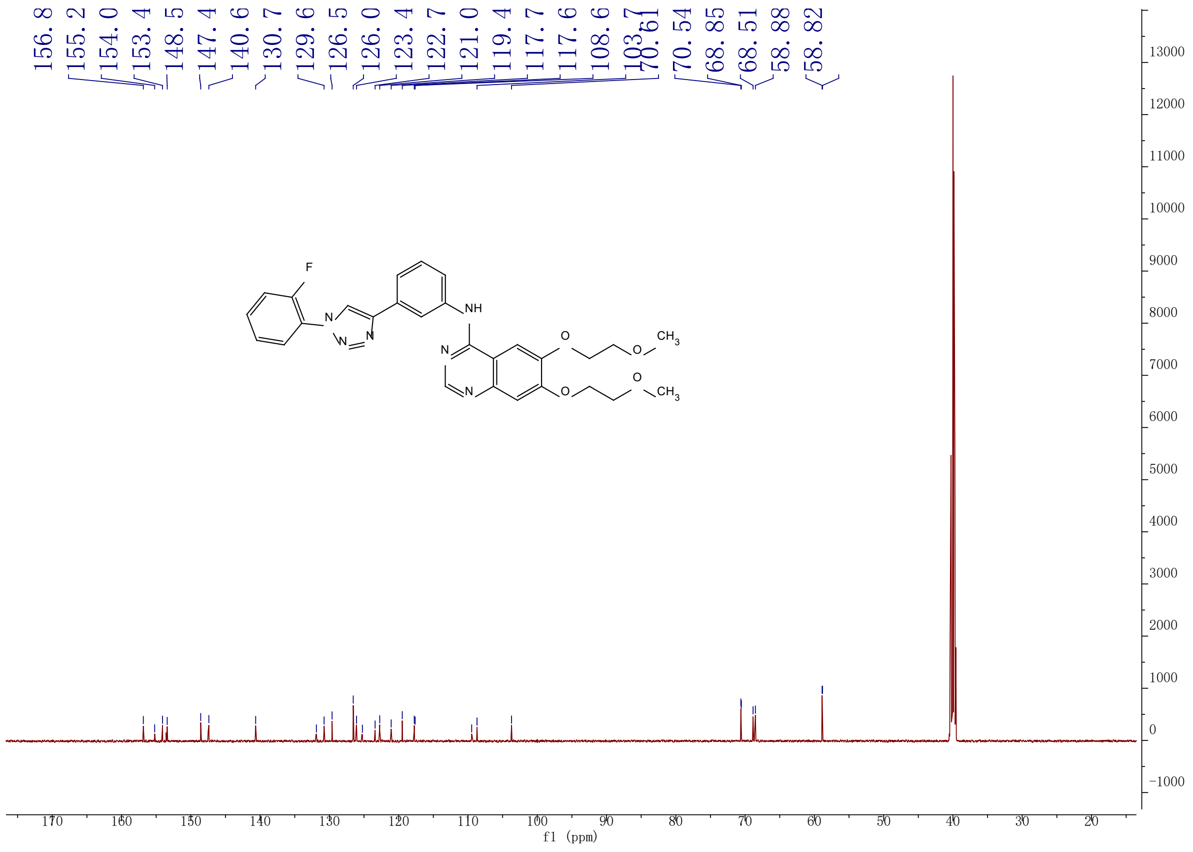

Figure S6-3. HR MS of compound e6

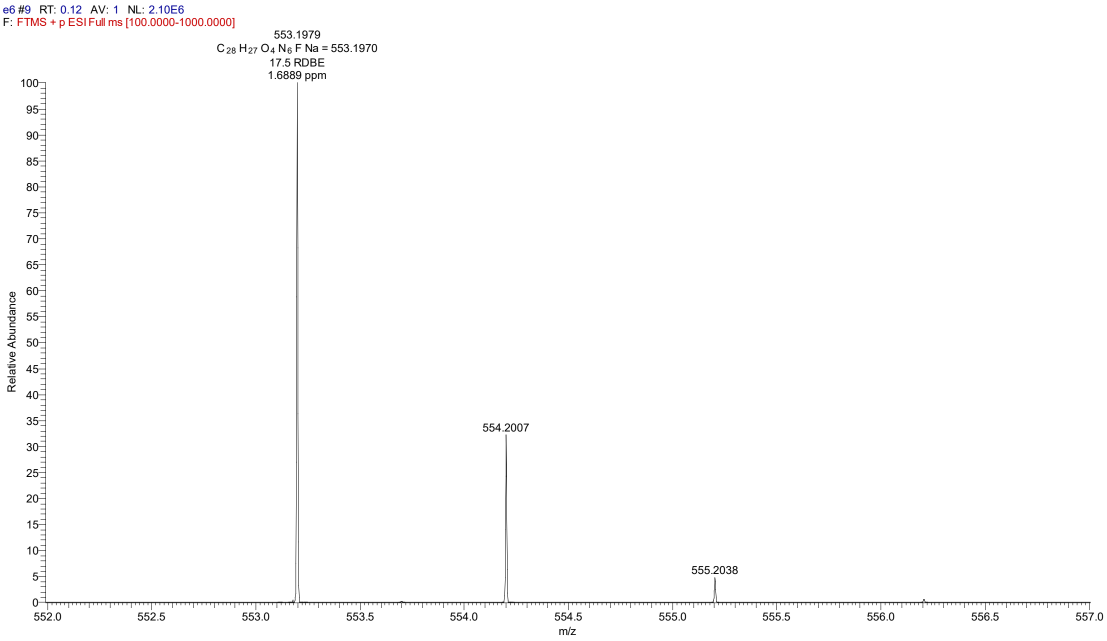

Figure S7-1. ^{1}H NMR spectrum (600 MHz, DMSO-d$_6$) of compound e7

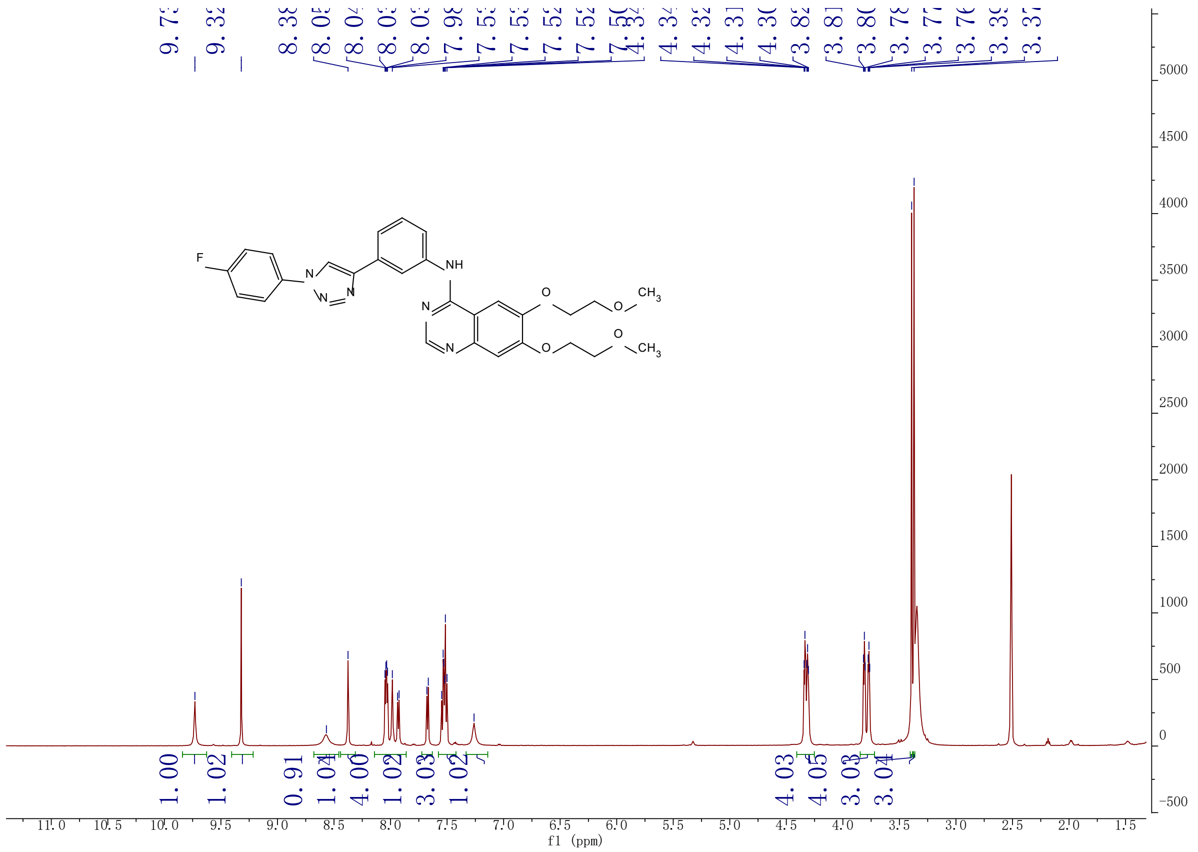

Figure S7-2. ^{13}C NMR spectrum (150 MHz, DMSO-d_6) of compound e7

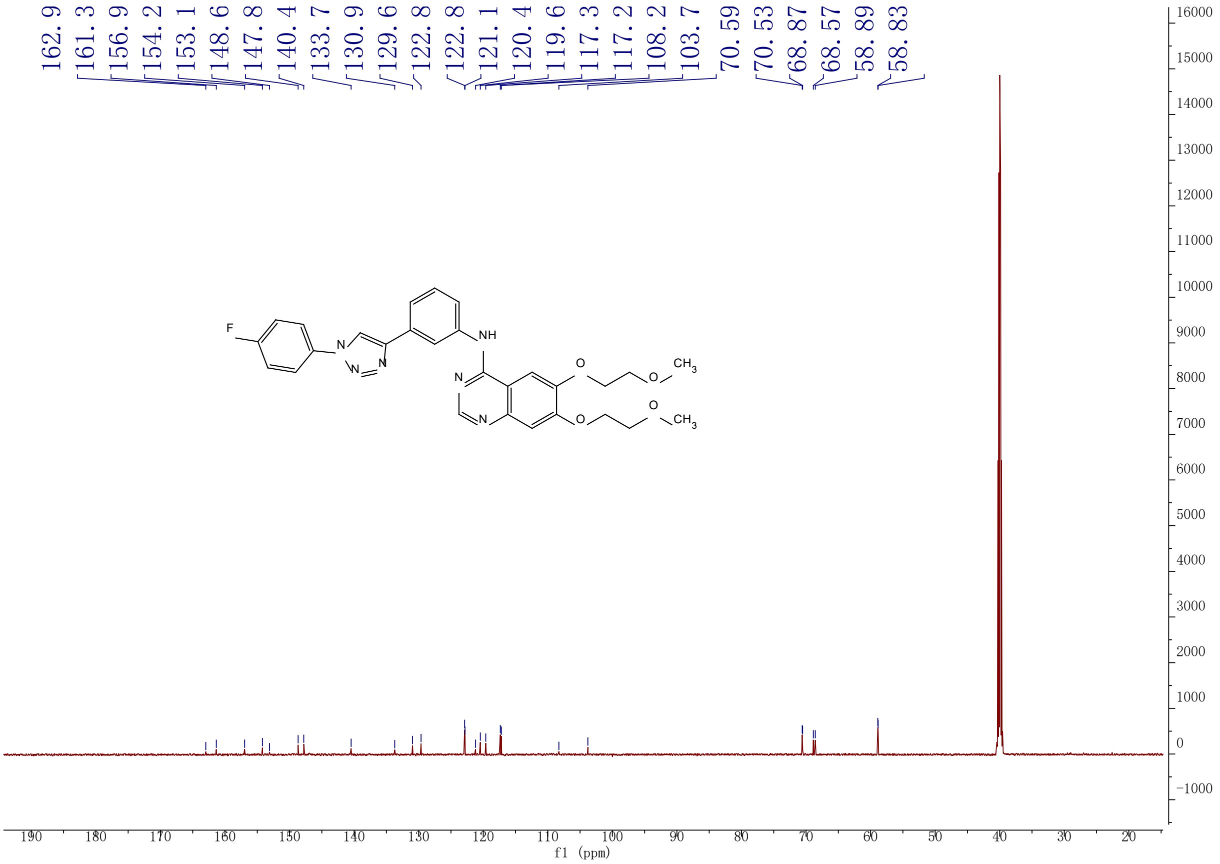

Figure S7-3. HR MS of compound e7

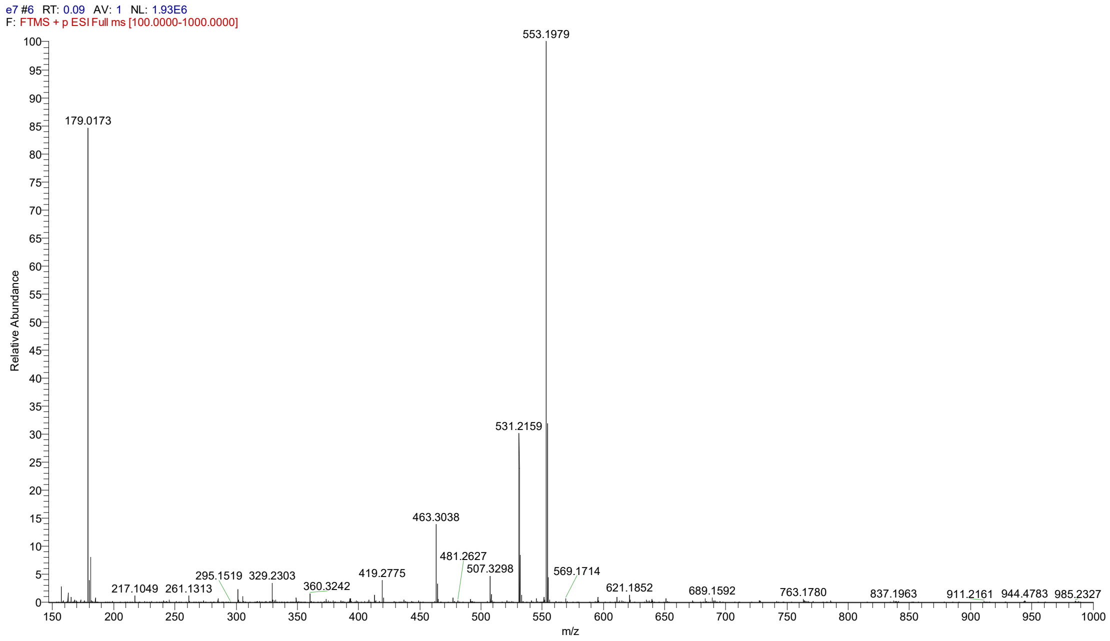

Figure S8-1. ^{1}H NMR spectrum (600 MHz, DMSO-d$_6$) of compound e8

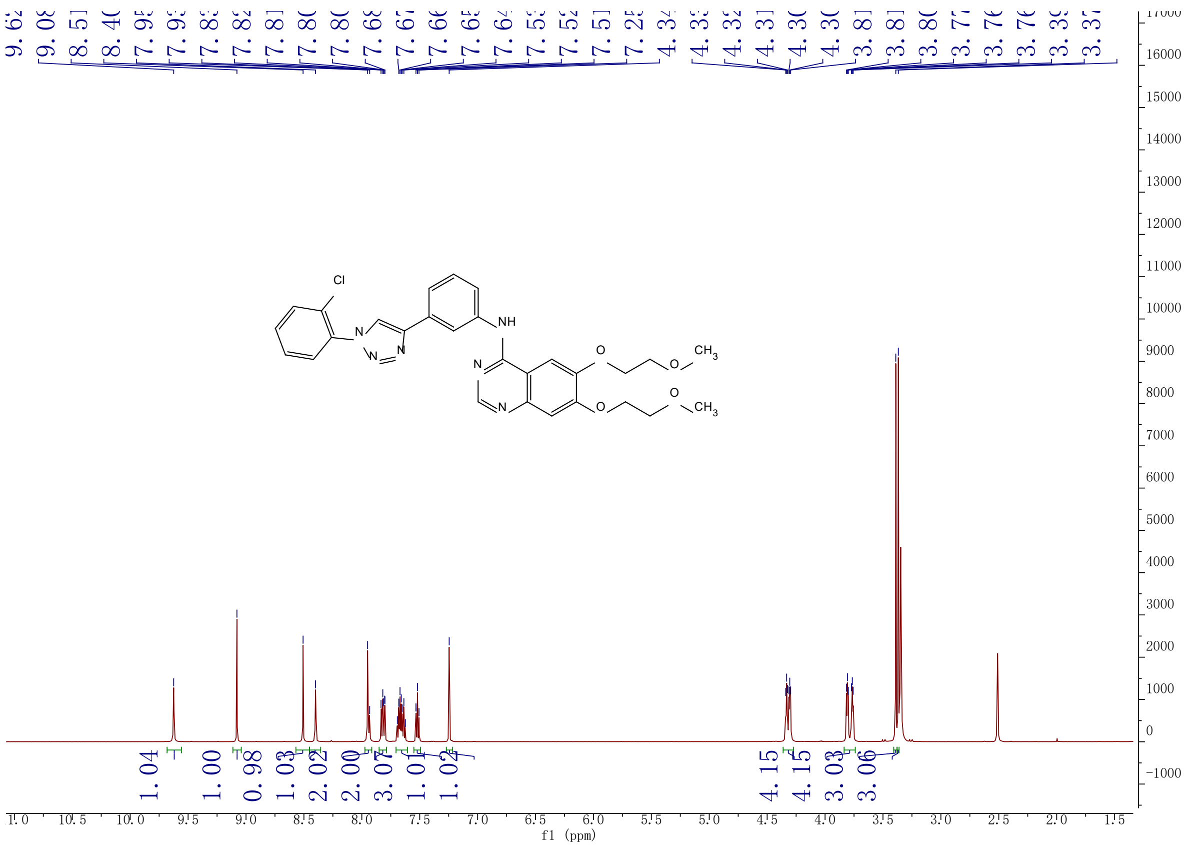

Figure S8-2. ^{13}C NMR spectrum (150 MHz, DMSO-d_6) of compound e8

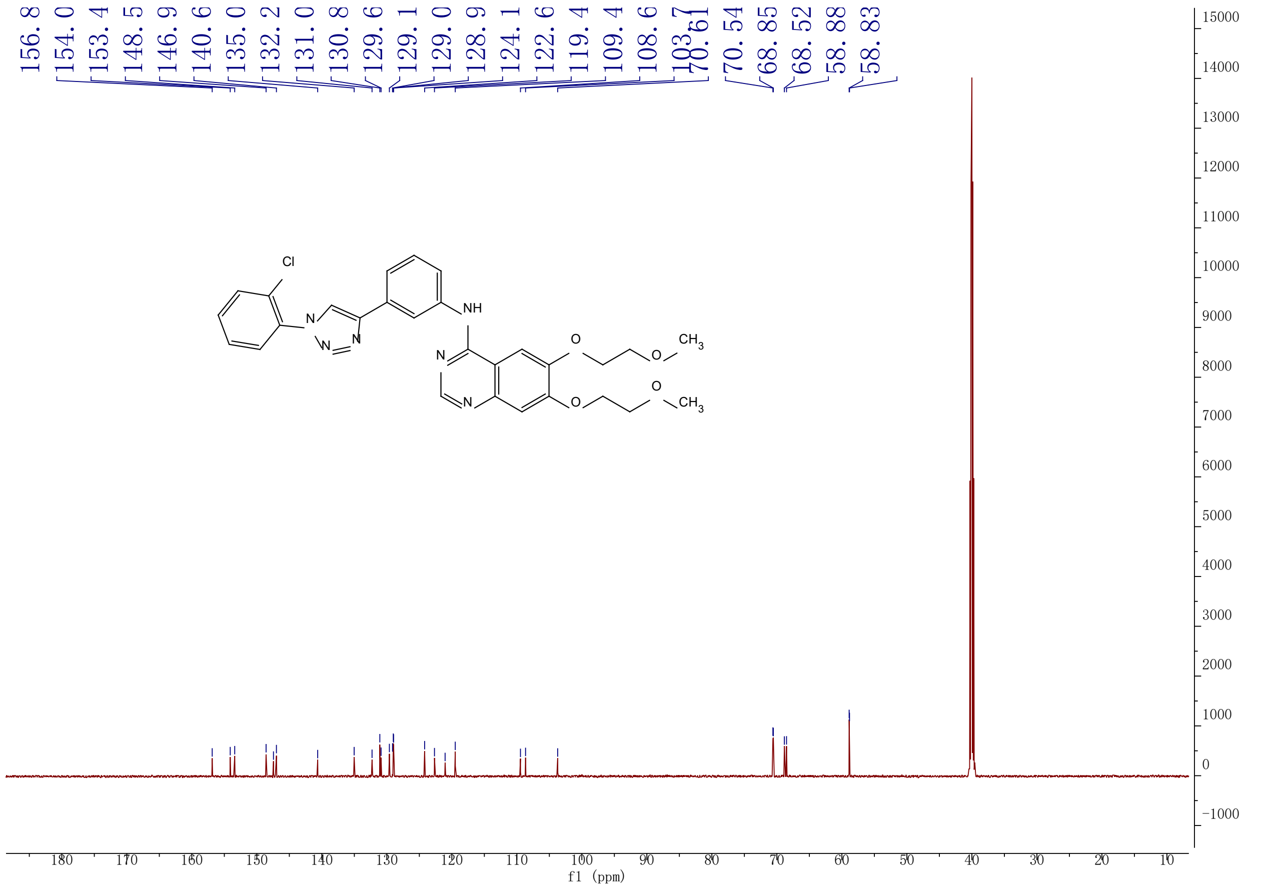

Figure S8-3. HR MS of compound e8

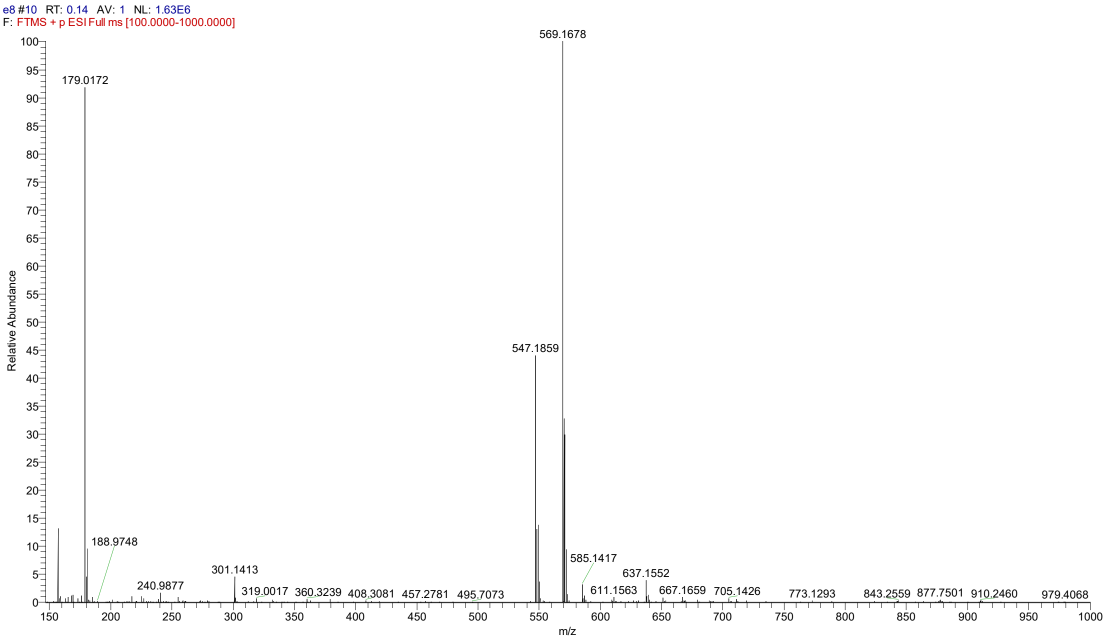

Figure S9-1. ^{1}H NMR spectrum (600 MHz, DMSO-d$_6$) of compound e9

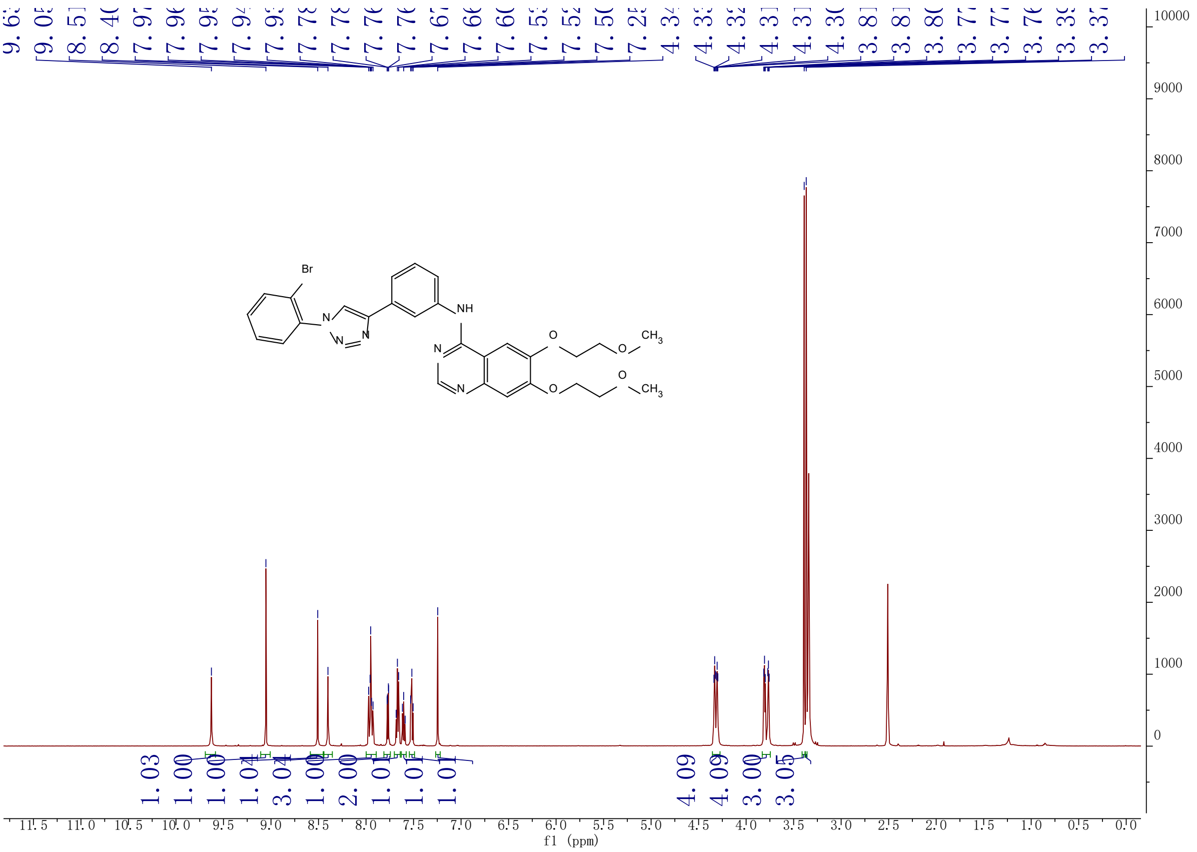

Figure S9-2. ^{13}C NMR spectrum (150 MHz, DMSO-d$_6$) of compound e9

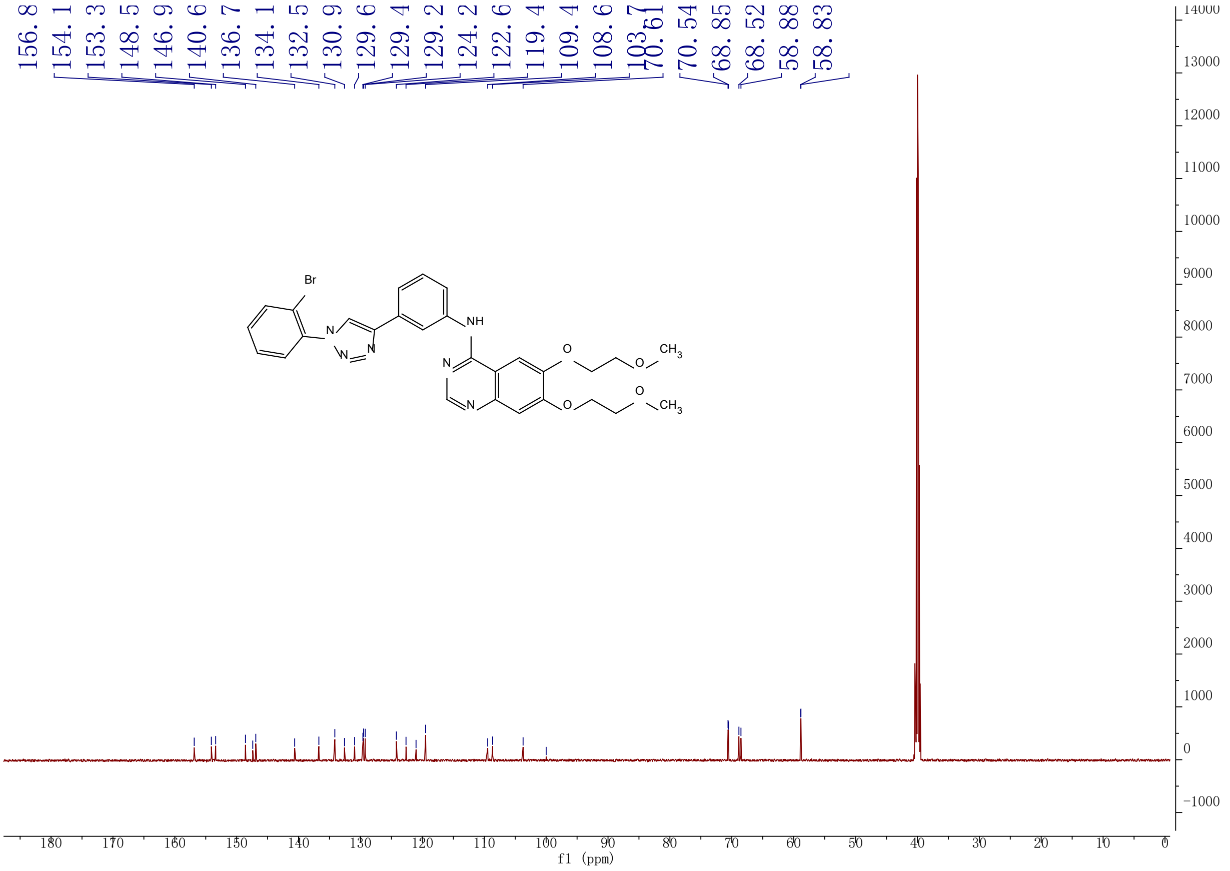

Figure S9-3. HR MS of compound e9

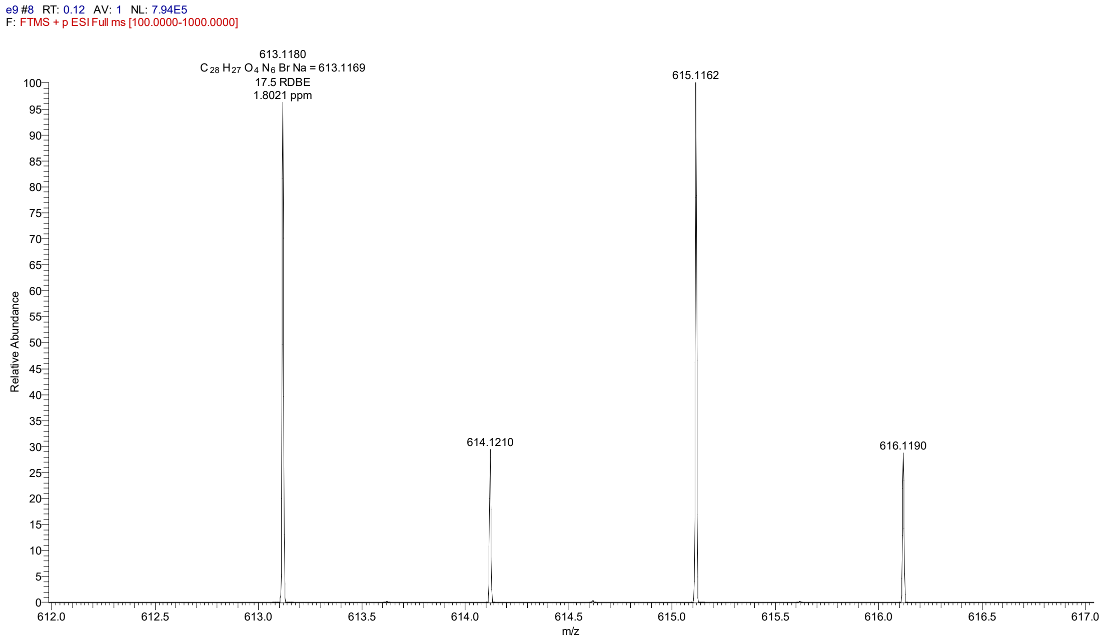

Figure S10-1. [1]**H NMR spectrum (600 MHz, DMSO-d$_6$) of compound e10**

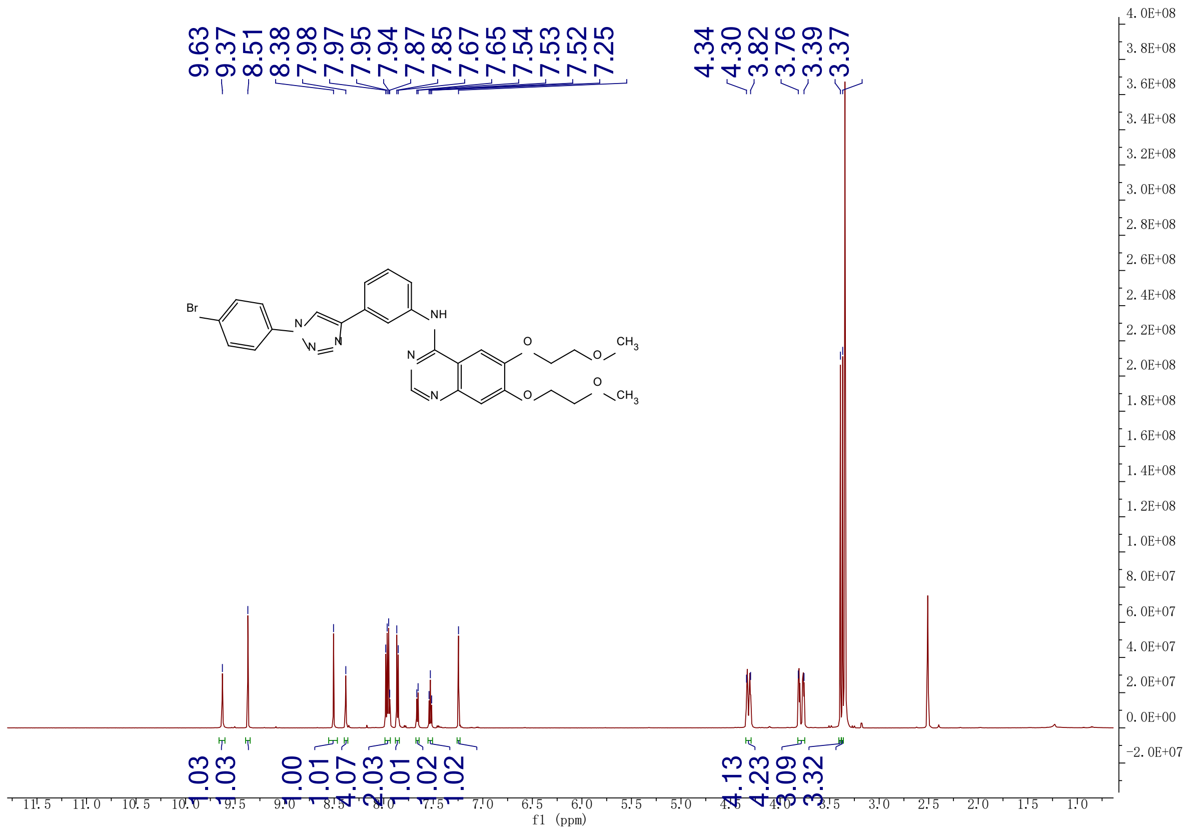

Figure S10-2. ^{13}C NMR spectrum (150 MHz, DMSO-d_6) of compound e10

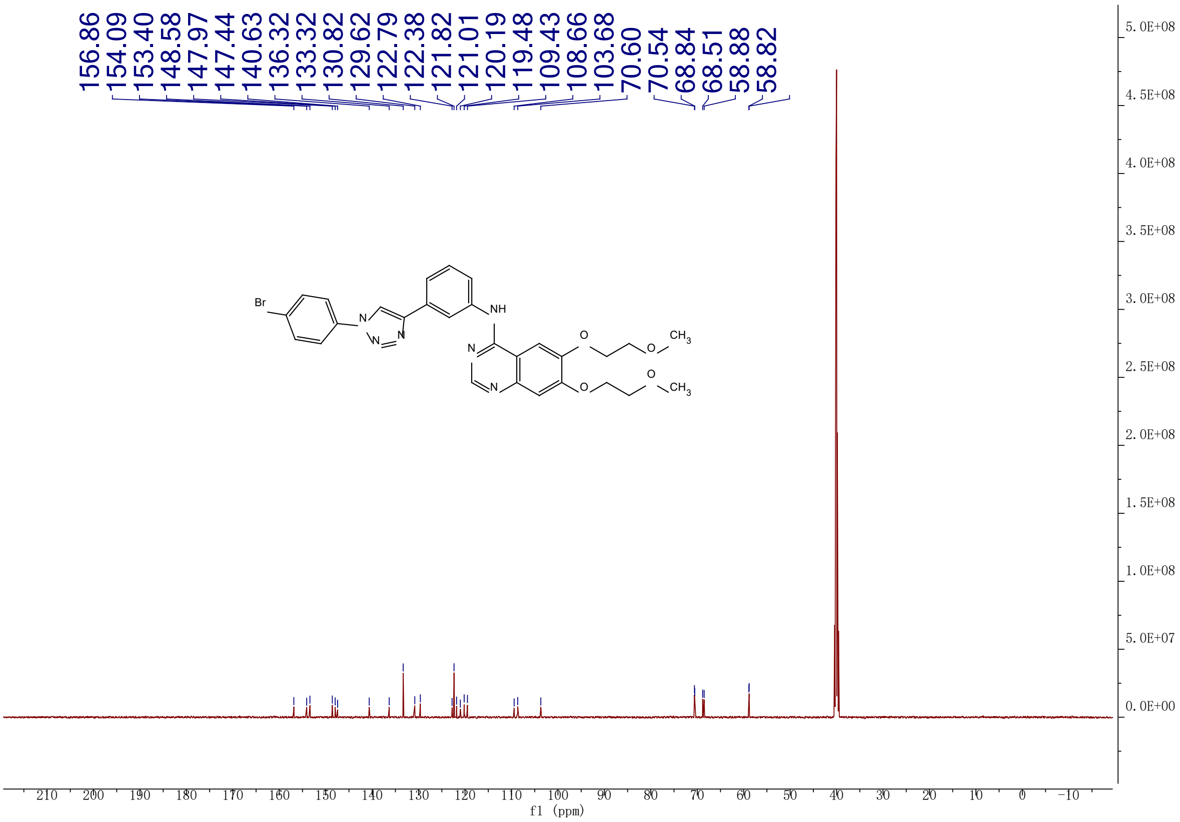

Figure S10-3. HR MS of compound e10

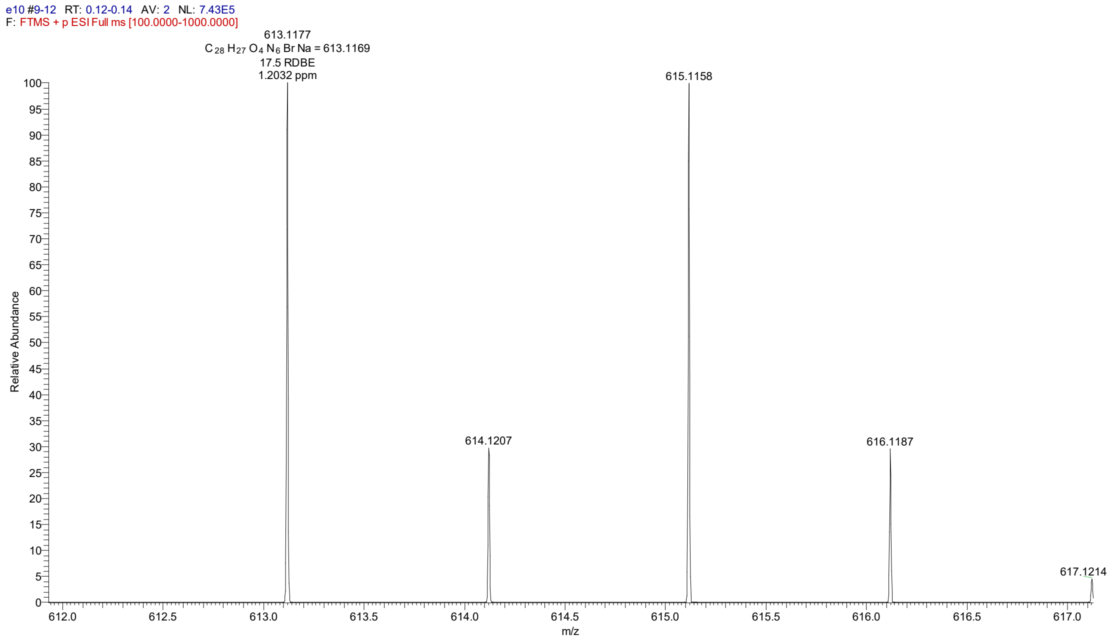

Figure S11-1. ^{1}H NMR spectrum (600 MHz, DMSO-d$_6$) of compound e11

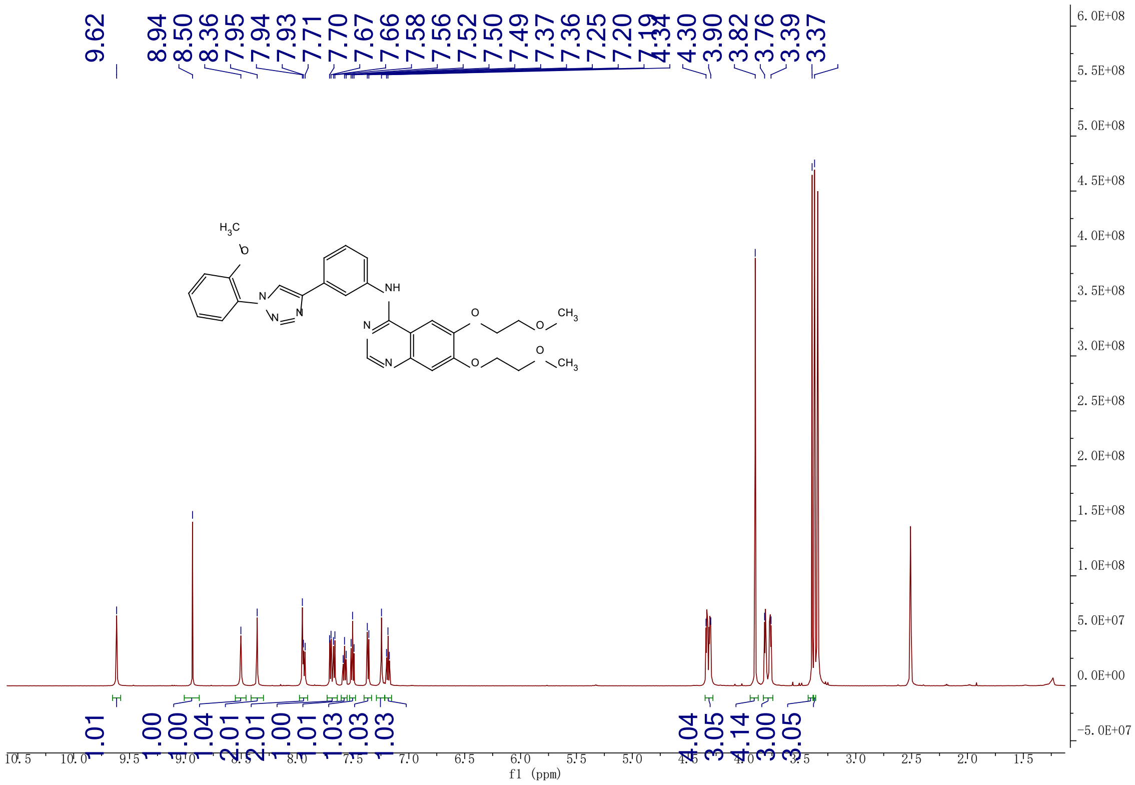

Figure S11-2. ^{13}C NMR spectrum (150 MHz, DMSO-d$_6$) of compound e11

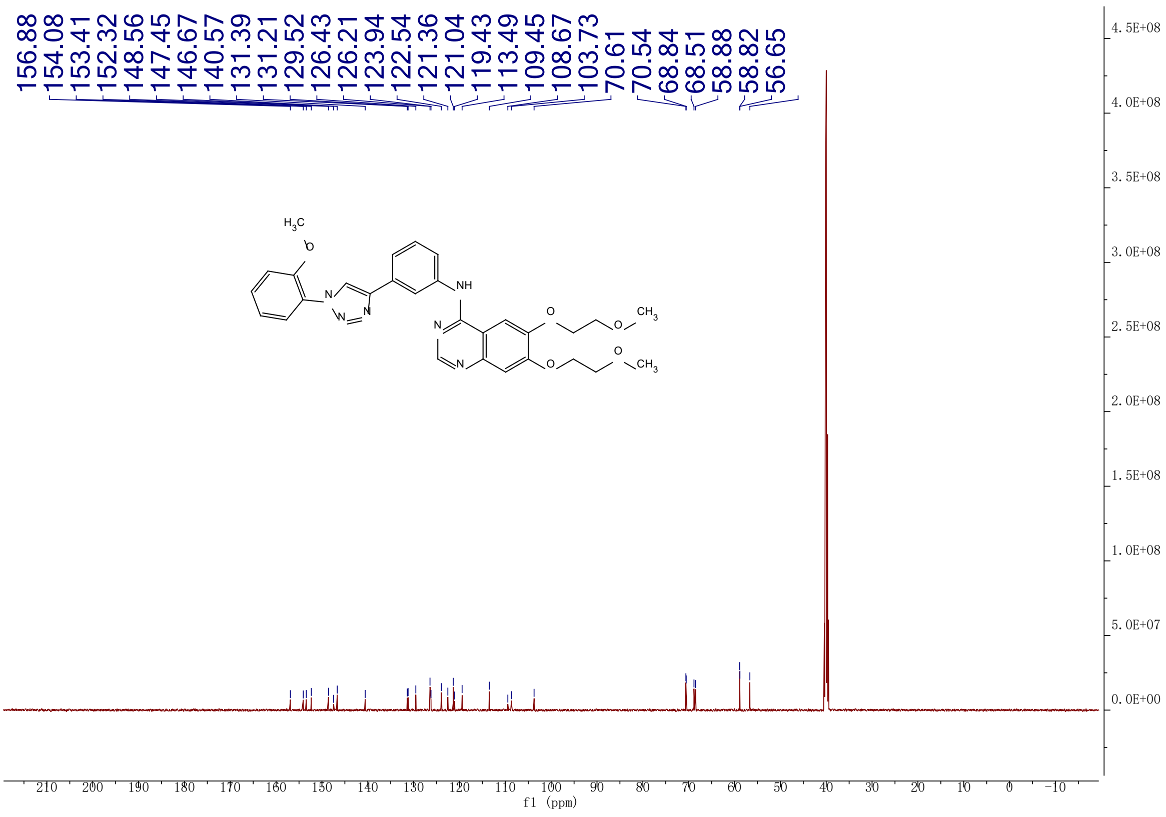

Figure S11-3. HR MS of compound e11

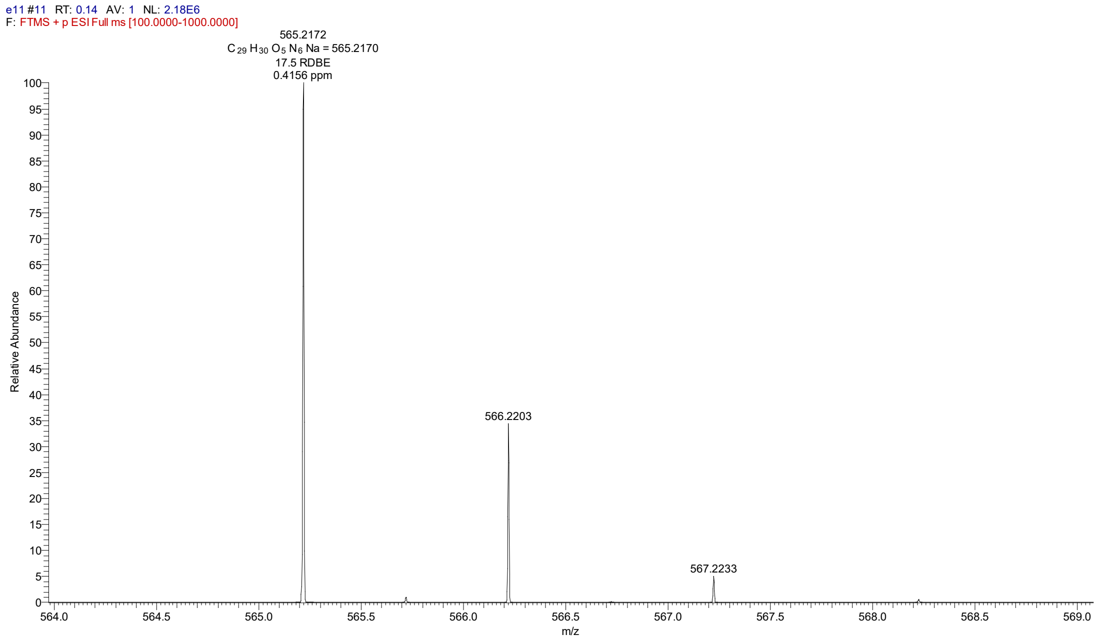

Figure S12-1. ^{1}H NMR spectrum (600 MHz, DMSO-d_6) of compound e12

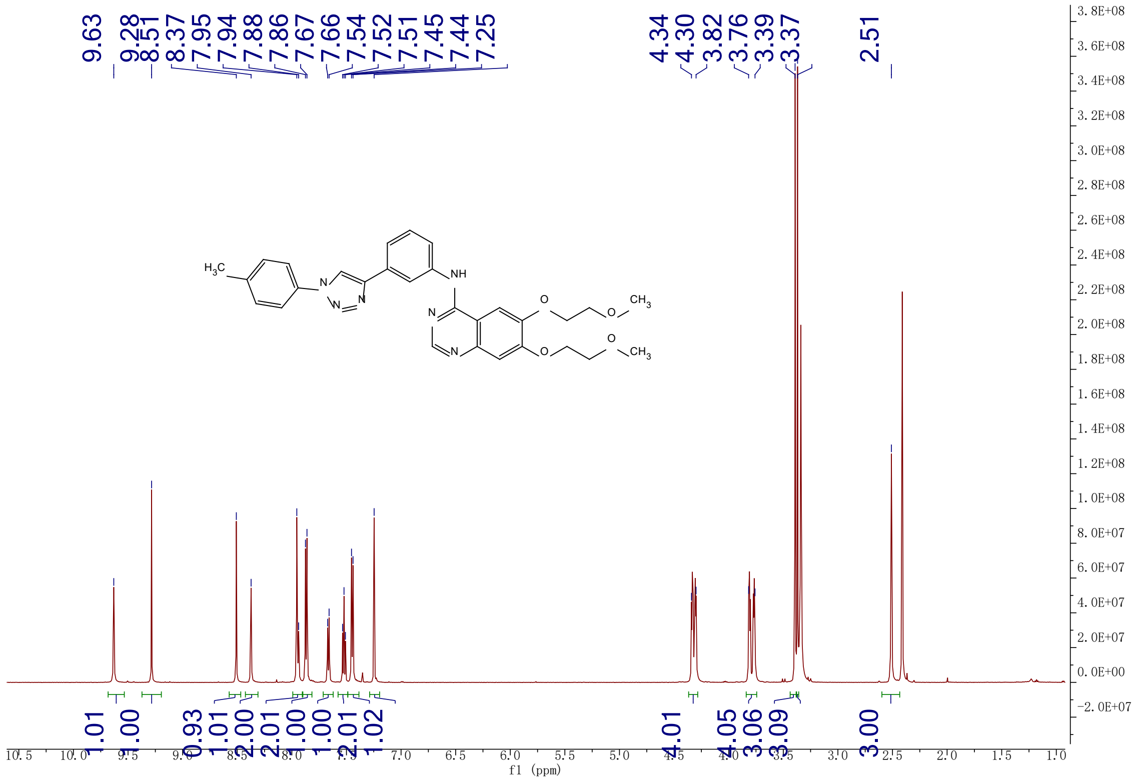

Figure S12-2. [12]C NMR spectrum (150 MHz, DMSO-d$_6$) of compound e12

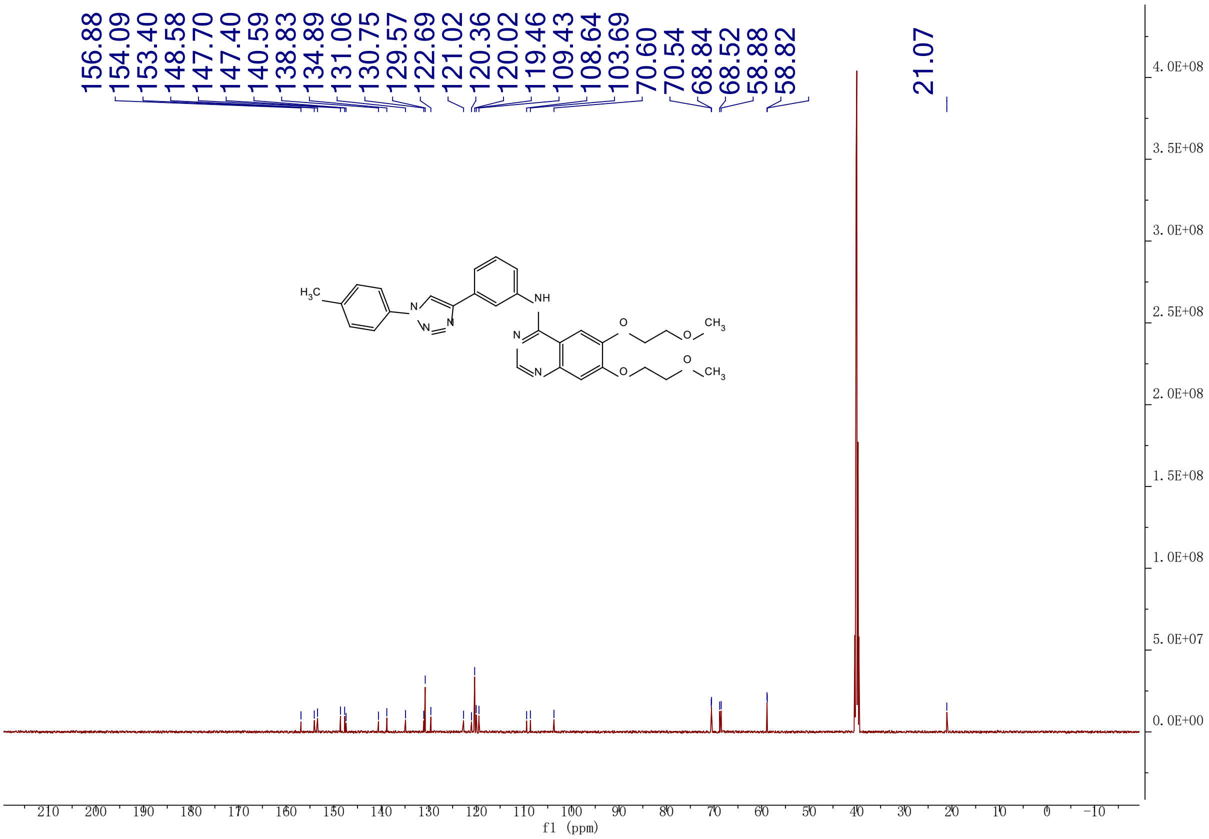

Figure S12-3. HR MS of compound e12

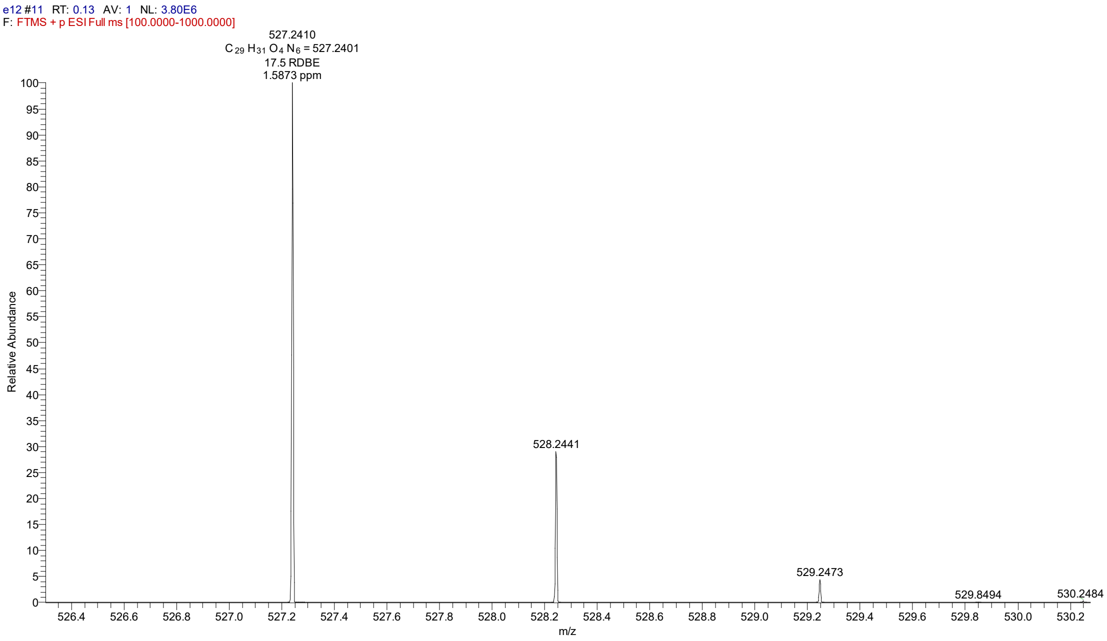

Figure S13-1. ^{1}H NMR spectrum (600 MHz, DMSO-d6) of compound a13

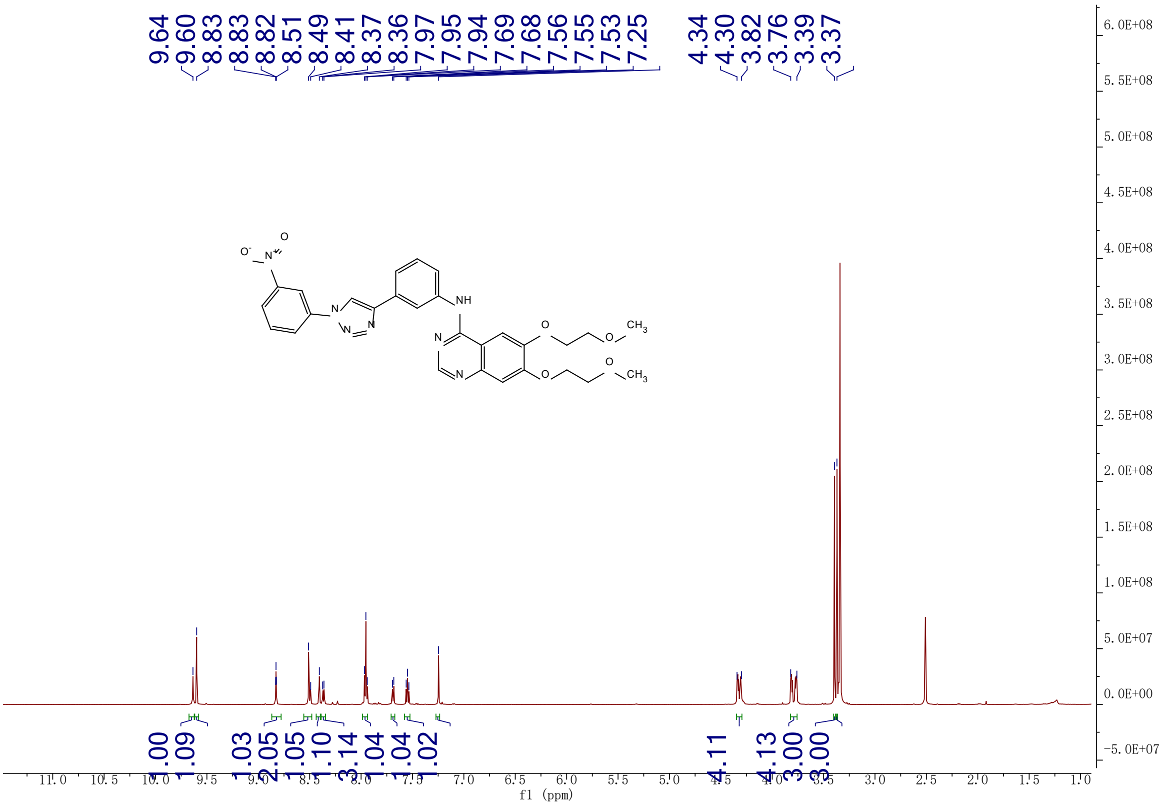

Figure S13-2. ^{13}C NMR spectrum (150 MHz, DMSO-d$_6$) of compound e13

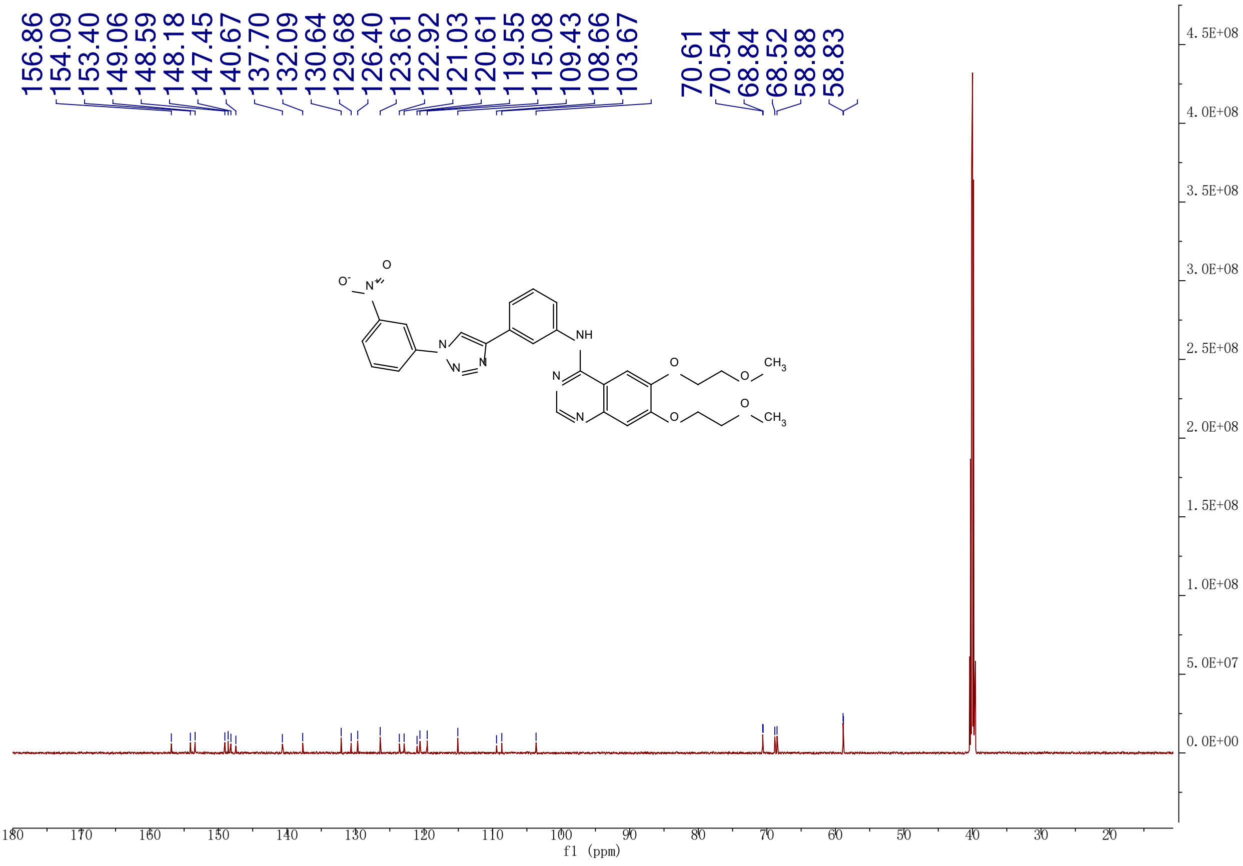

Figure S13-3. HR MS of compound e13

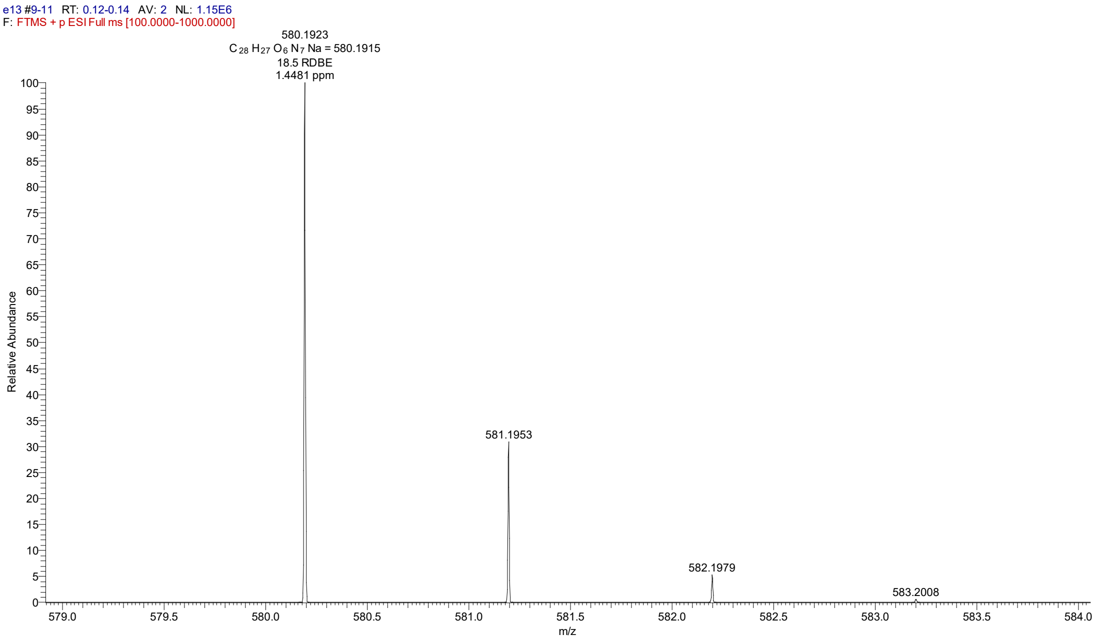

Figure S14-1. ^{1}H NMR spectrum (600 MHz, DMSO-d_6) of compound e14

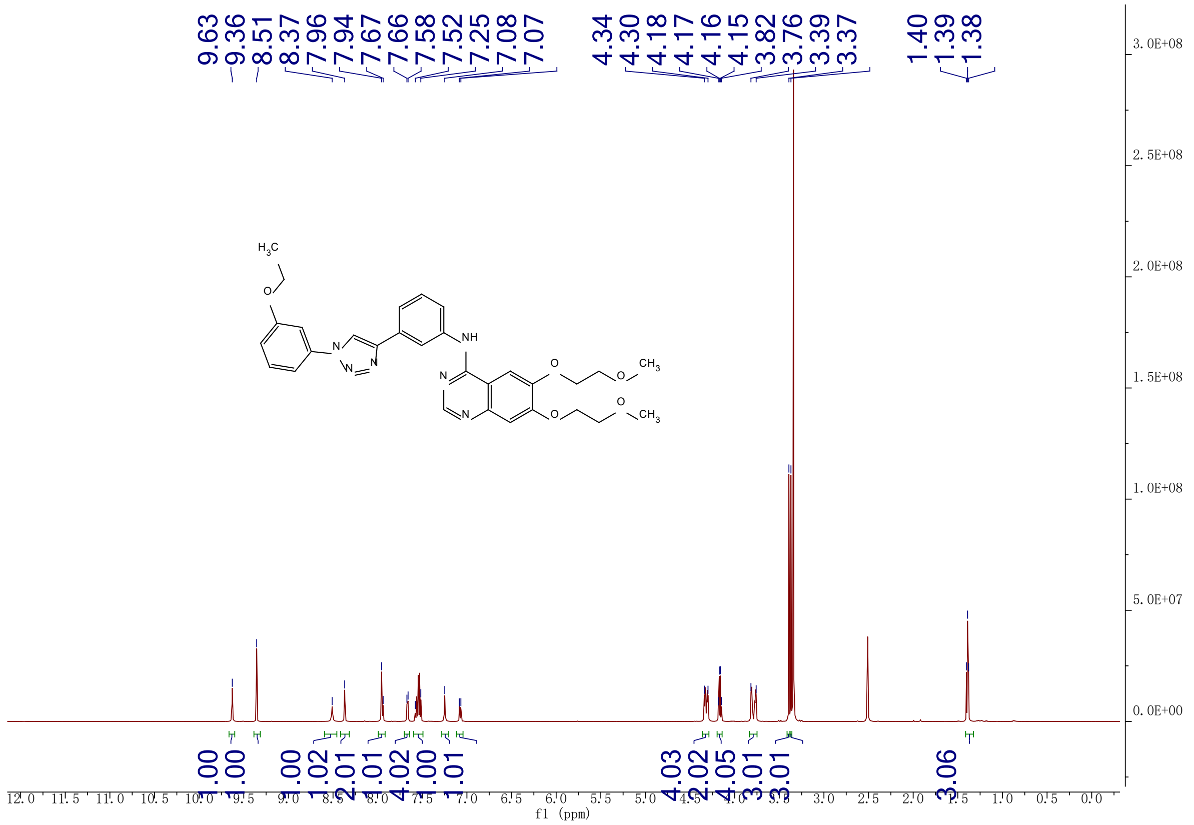

Figure S14-2. ^{13}C NMR spectrum (150 MHz, DMSO-d_6) of compound e14

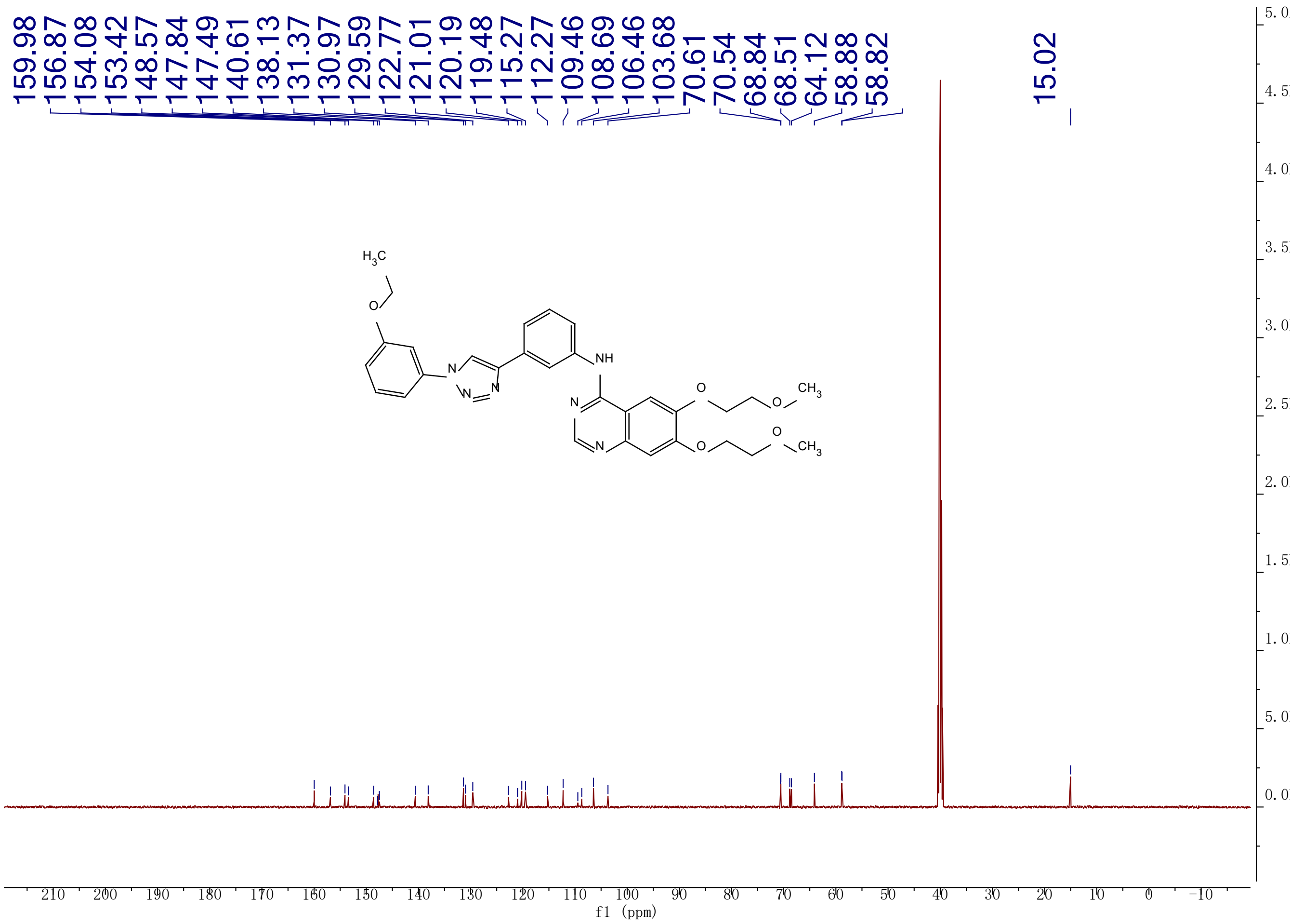

Figure S14-3. HR MS of compound e14

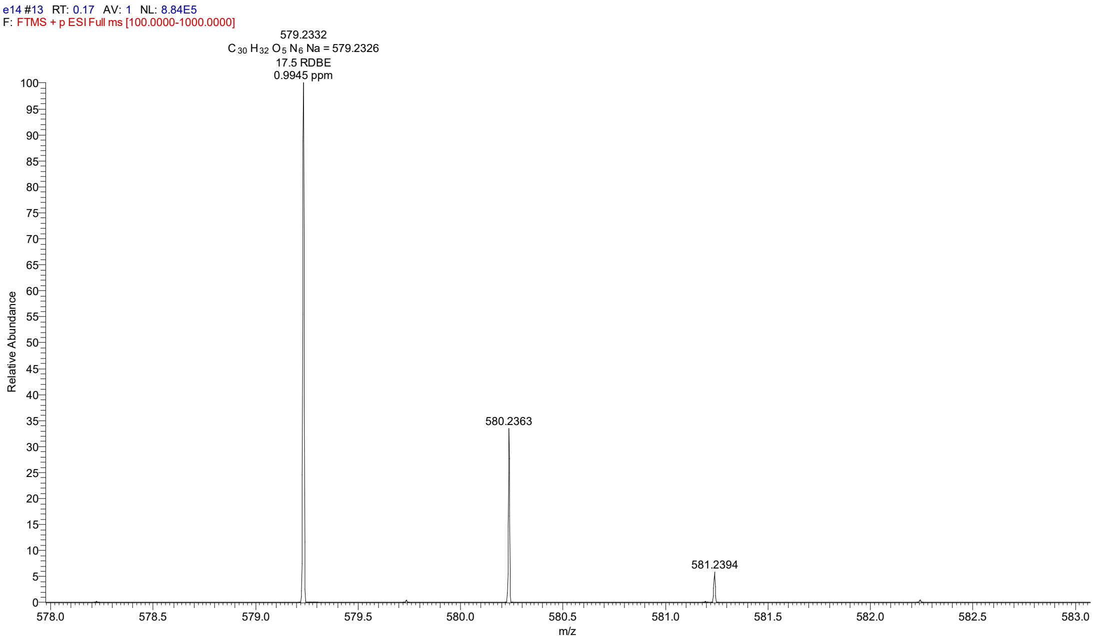

Figure S15-1. ^{1}H NMR spectrum (600 MHz, DMSO-d$_6$) of compound e15

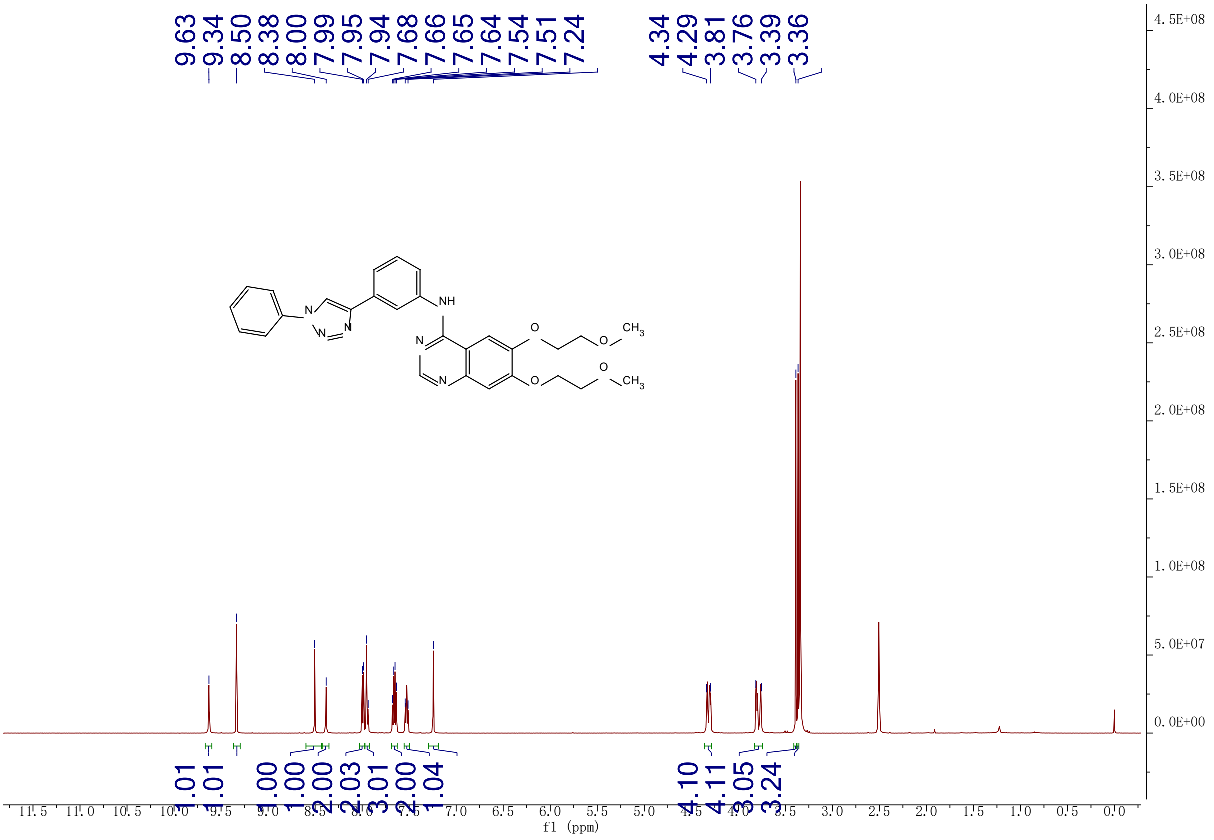

Figure S15-2. ^{13}C NMR spectrum (150 MHz, DMSO-d_6) of compound e15

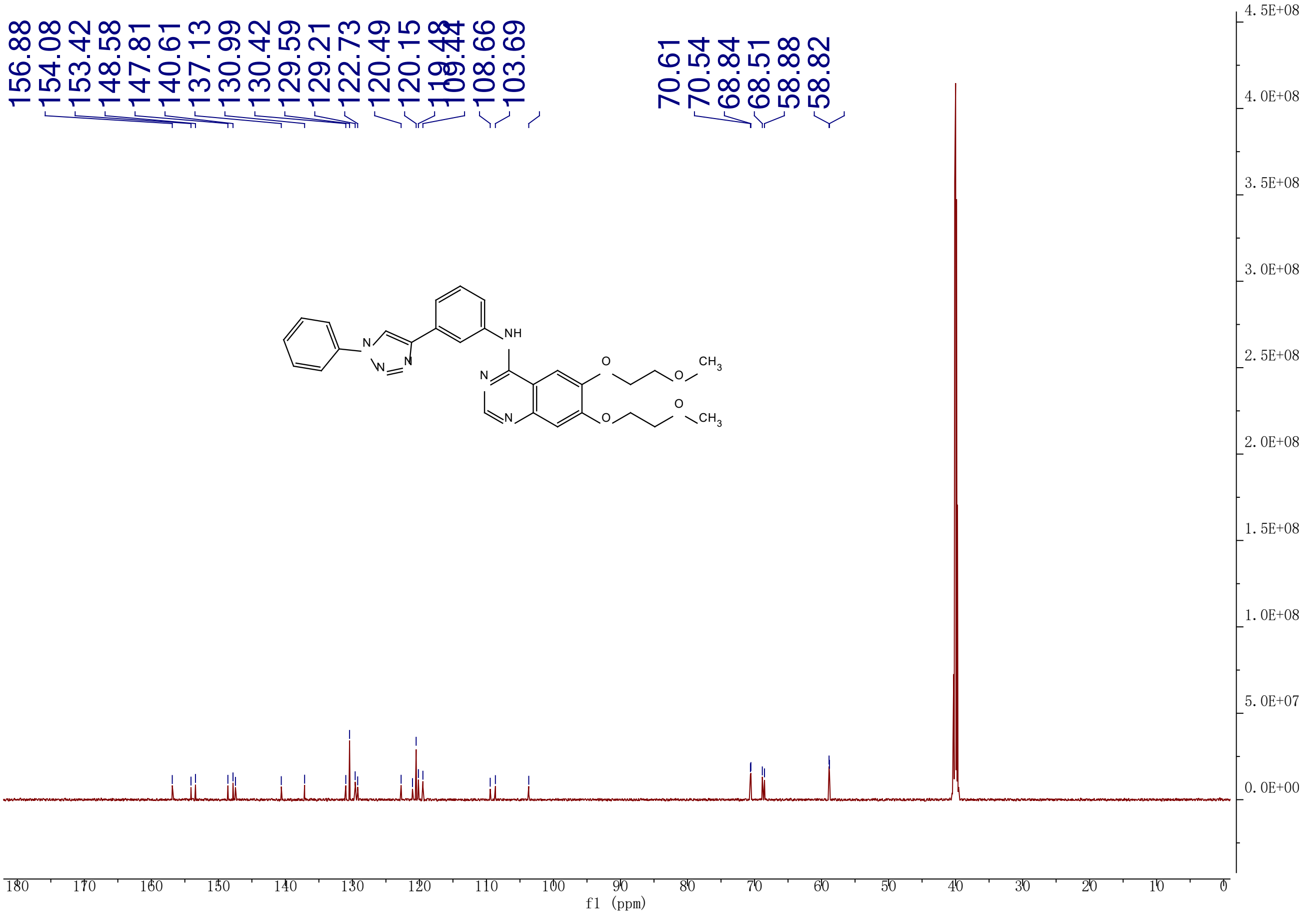

Figure S15-3. HR MS of compound e15

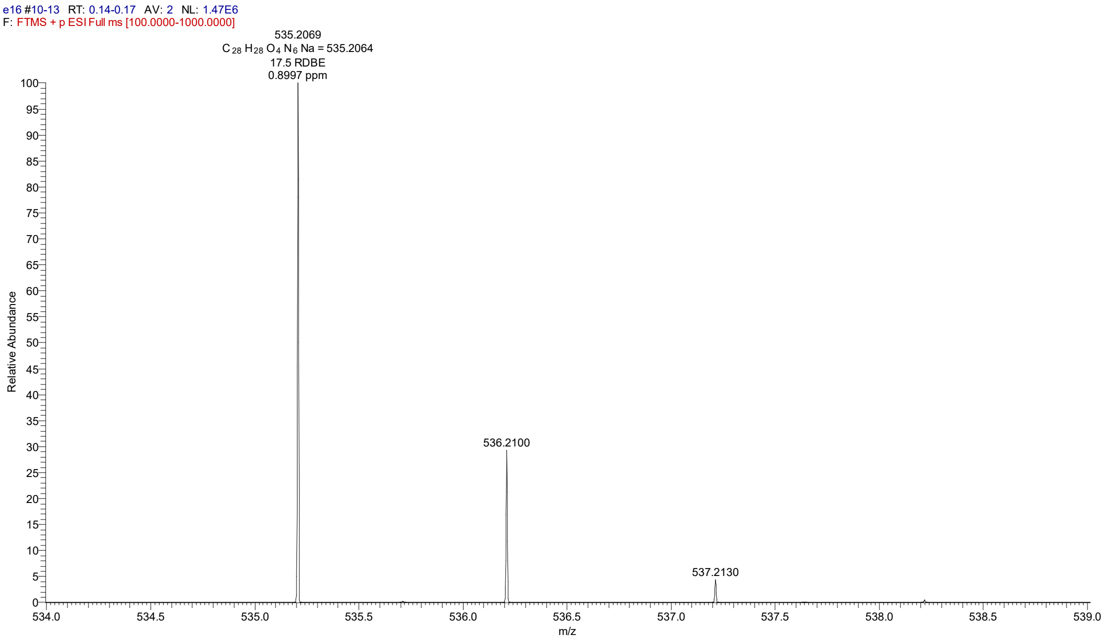

Figure S16-1. ^{1}H NMR spectrum (400 MHz, DMSO-d$_6$) of compound e16

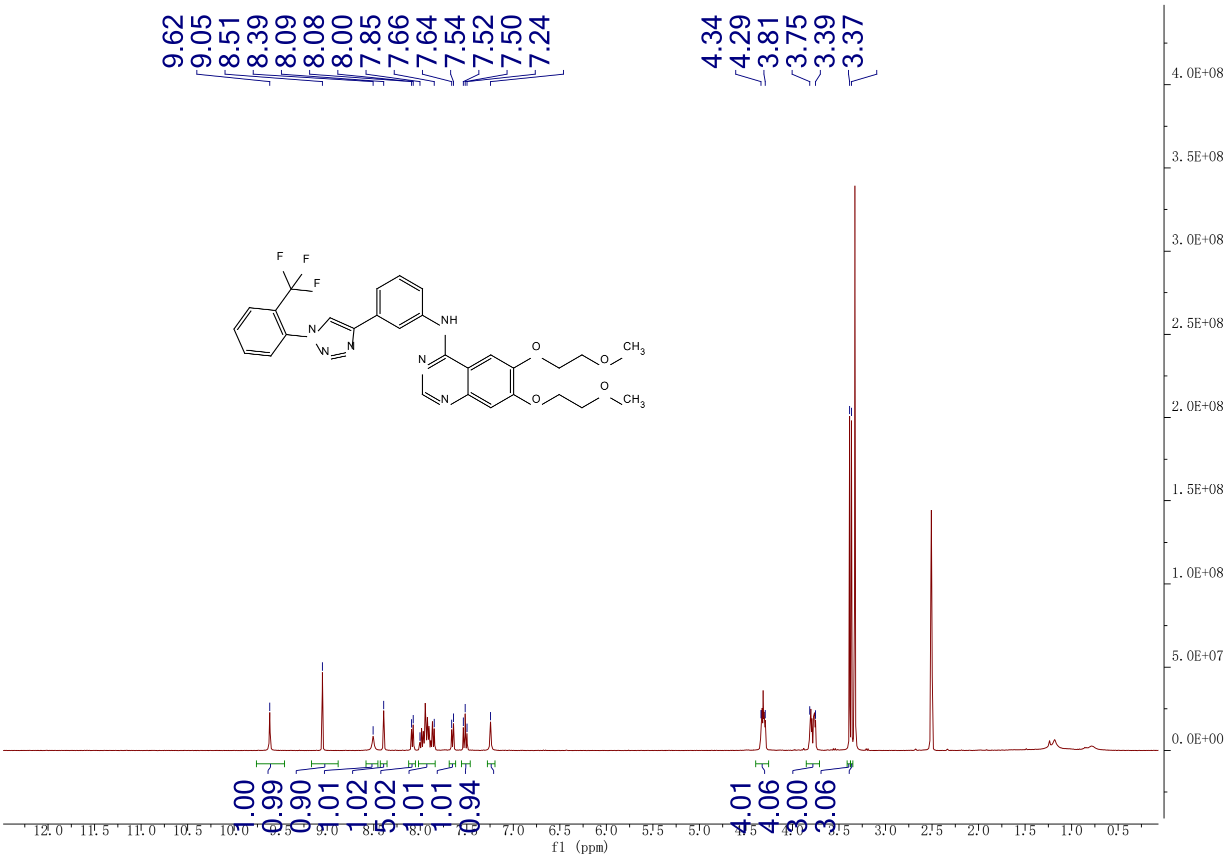

Figure S16-2. ^{13}C NMR spectrum (100 MHz, DMSO-d_6) of compound e16

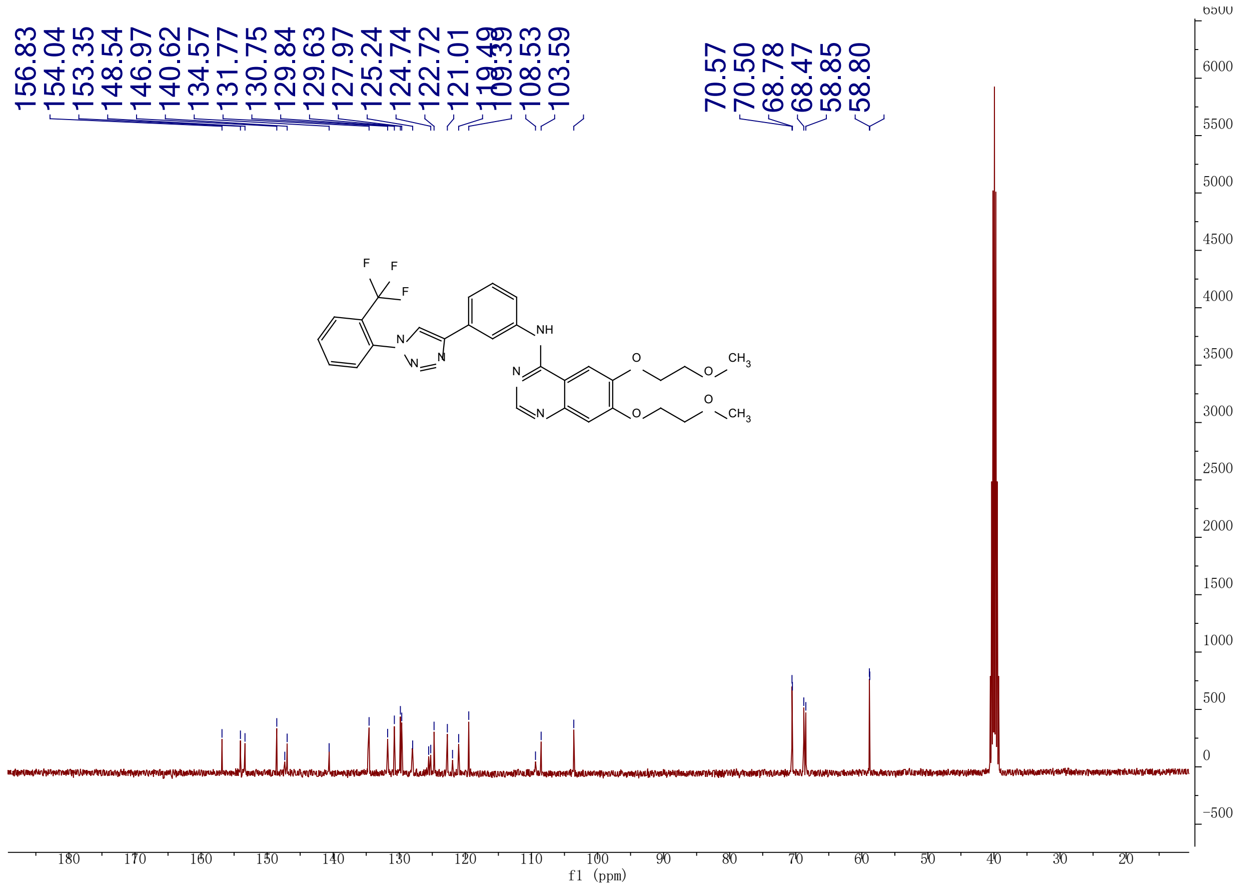

Figure S16-3. HR MS of compound e16

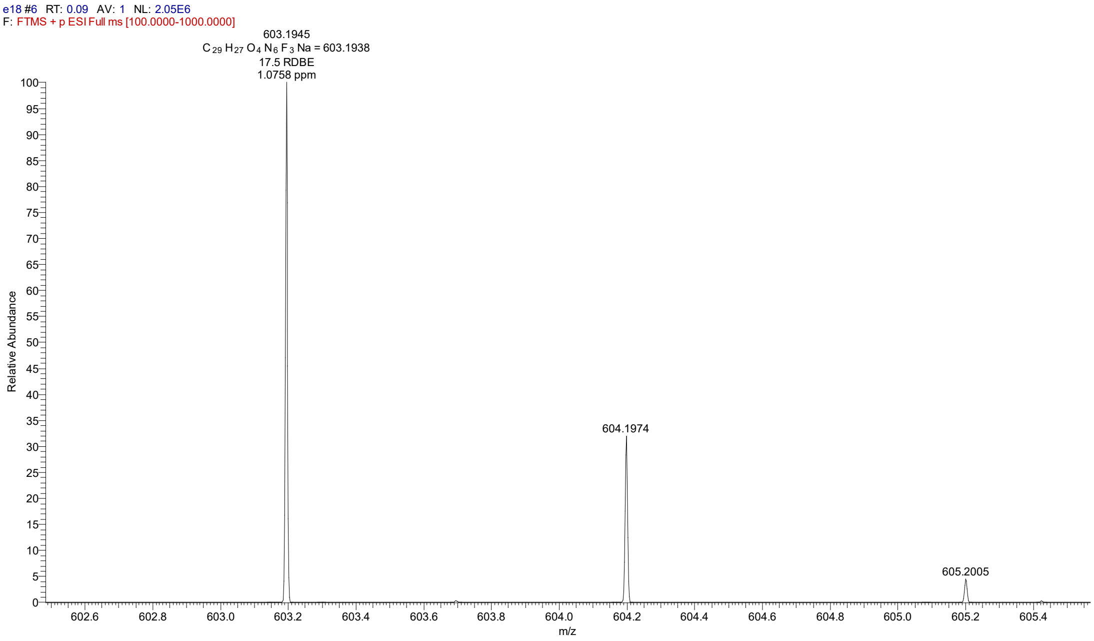

Figure S17-1. ^{1}H NMR spectrum (400 MHz, DMSO-d$_6$) of compound e17

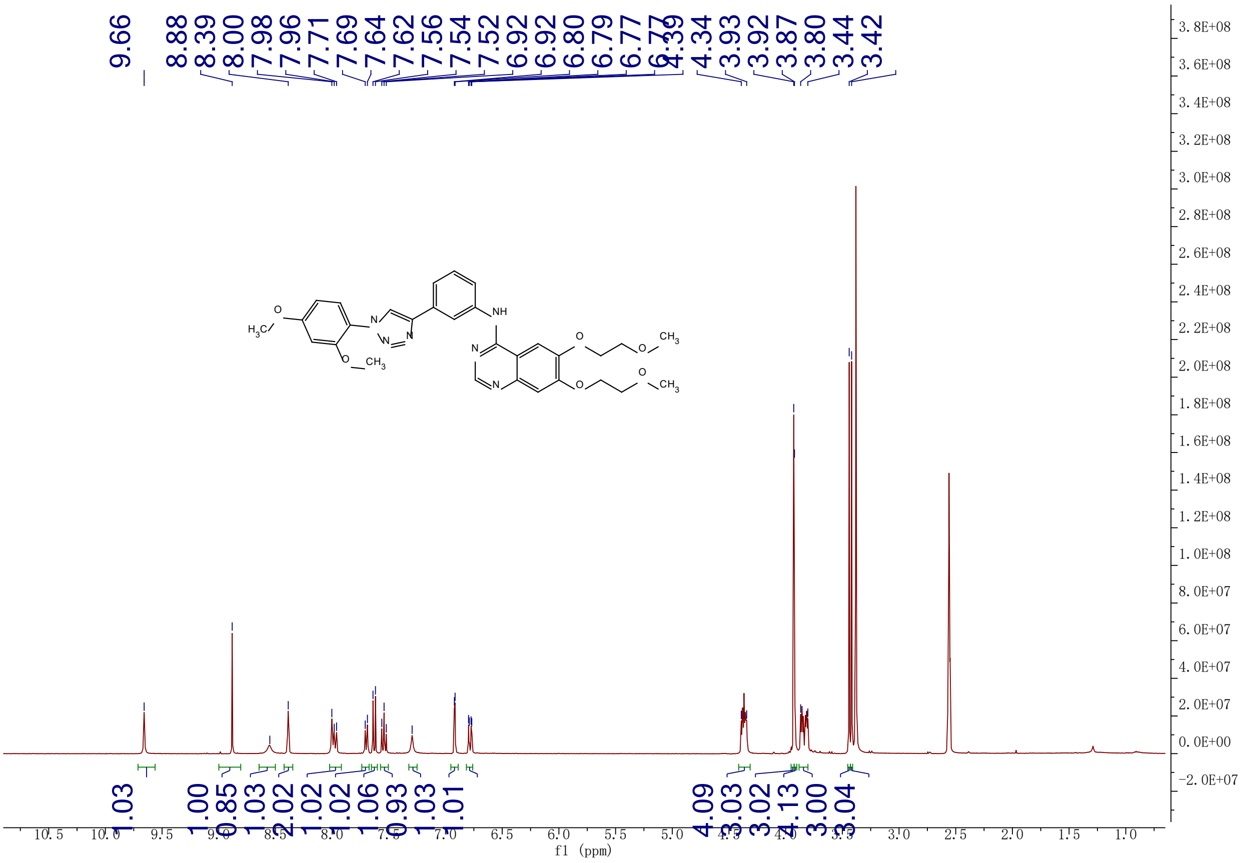

Figure S17-2. ^{13}C NMR spectrum (100 MHz, DMSO-d$_6$) of compound e17

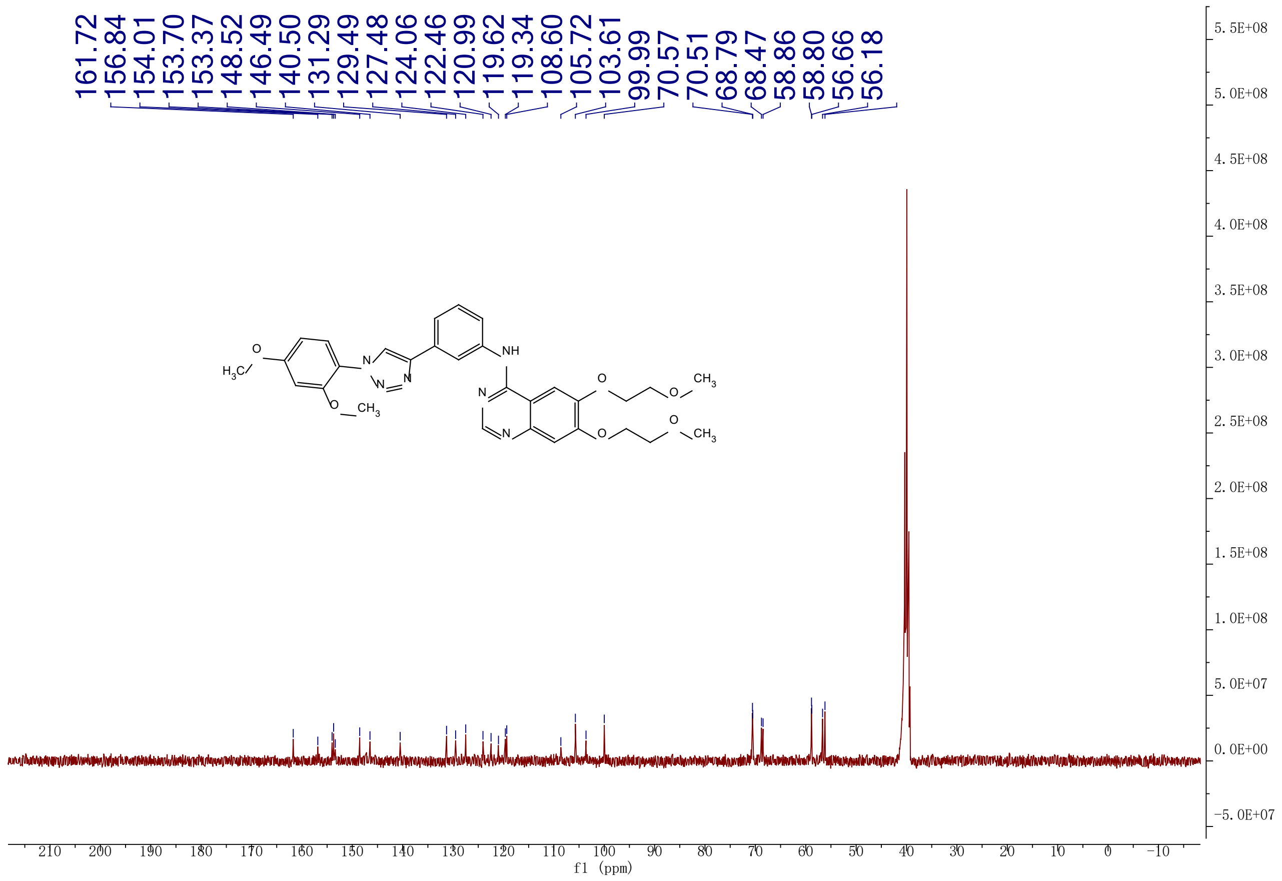

Figure S17-3. HR MS of compound e17

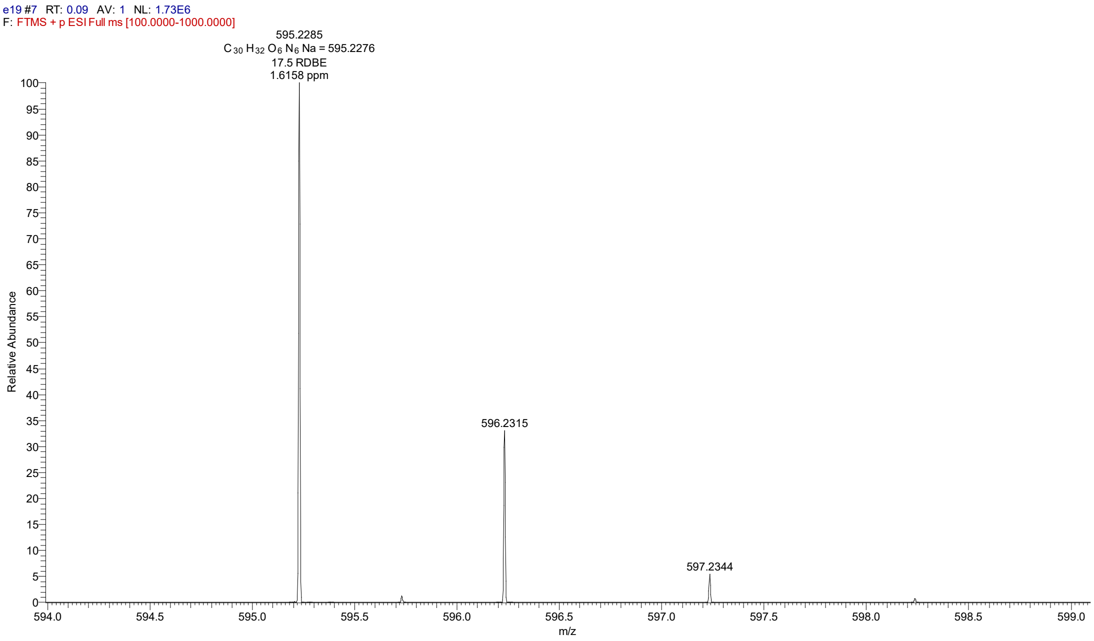

Figure S18-1. ^{1}H NMR spectrum (400 MHz, DMSO-d6) of compound e18

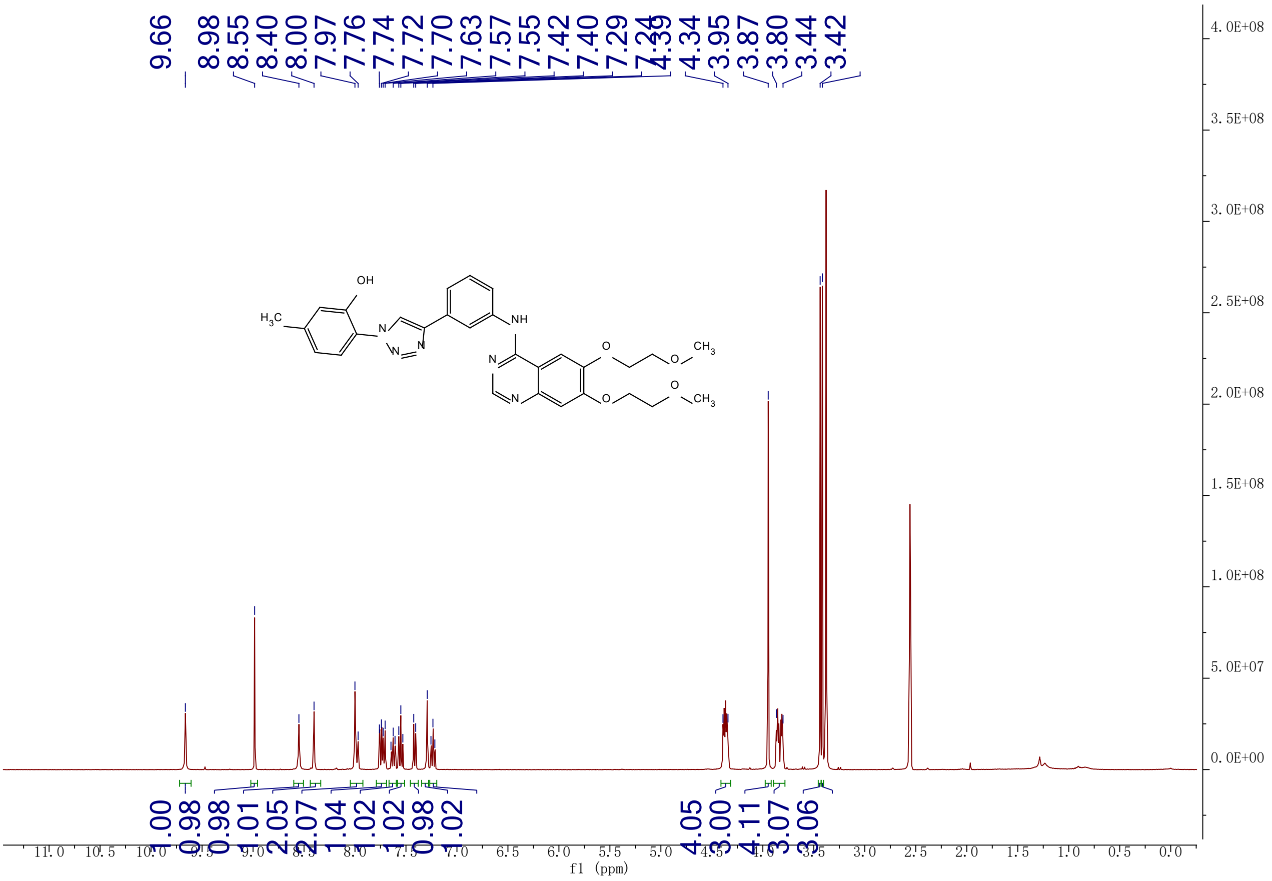

Figure S18-2. ^{13}C NMR spectrum (100 MHz, DMSO-d$_6$) of compound e18

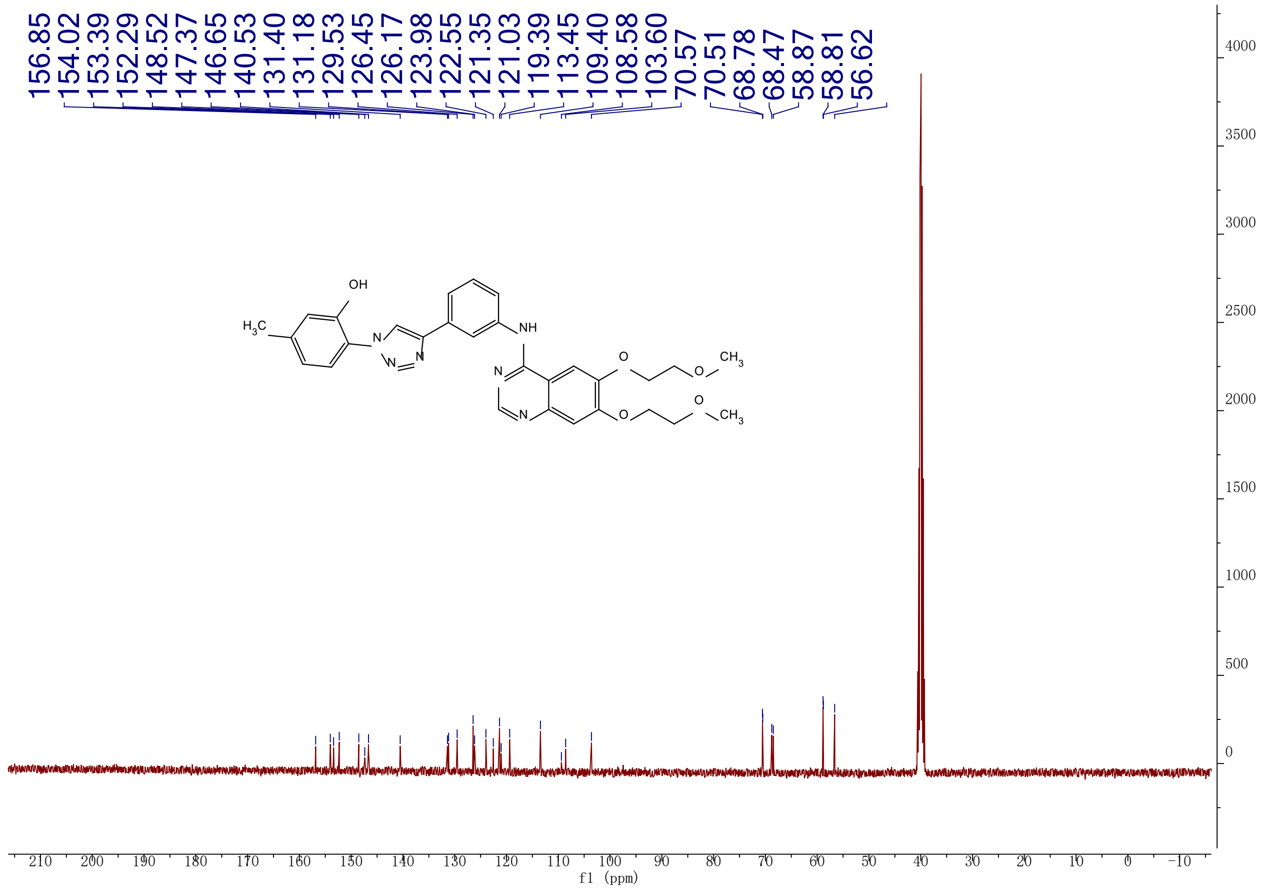

Figure S18-3. HR MS of compound e18

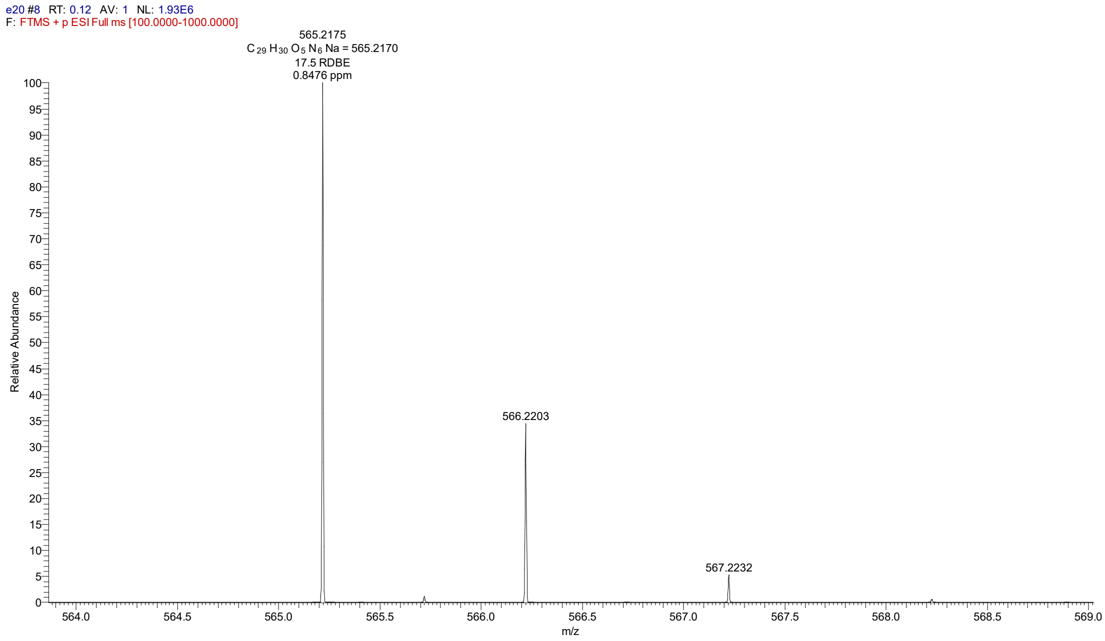

Figure S19-1. ¹H NMR spectrum (400 MHz, DMSO-d_6) of compound e19

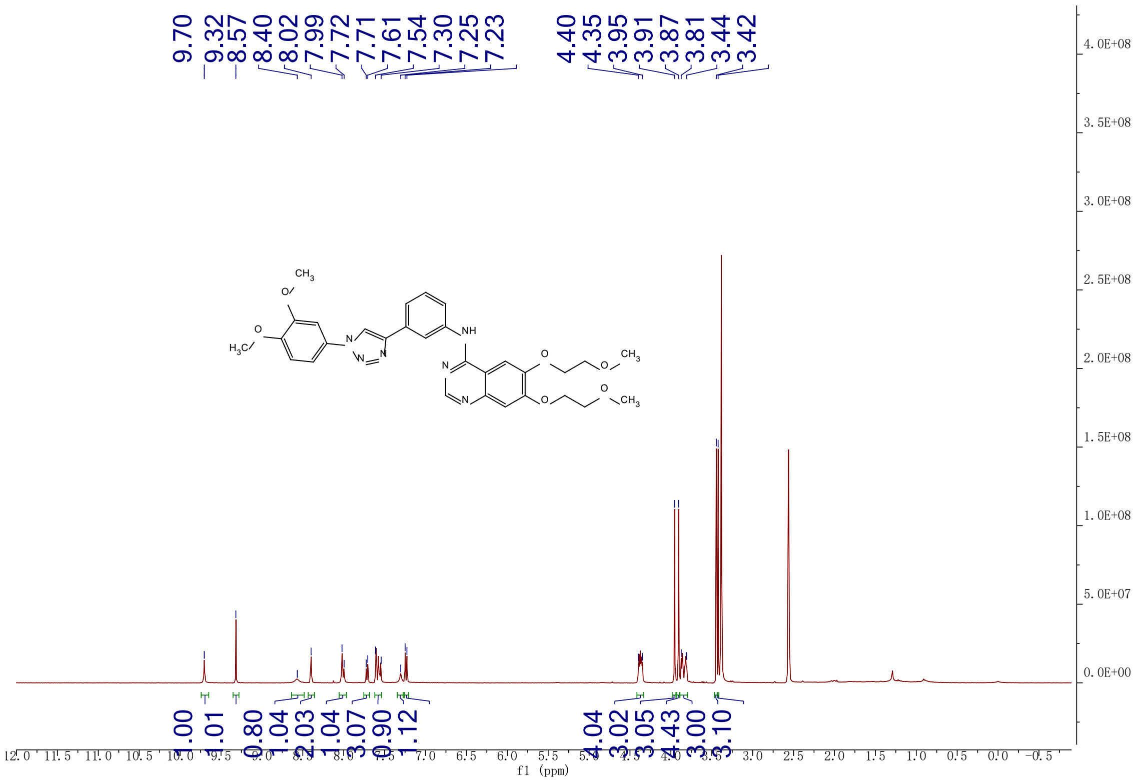

Figure S19-2. ^{13}C NMR spectrum (100 MHz, DMSO-d$_6$) of compound e19

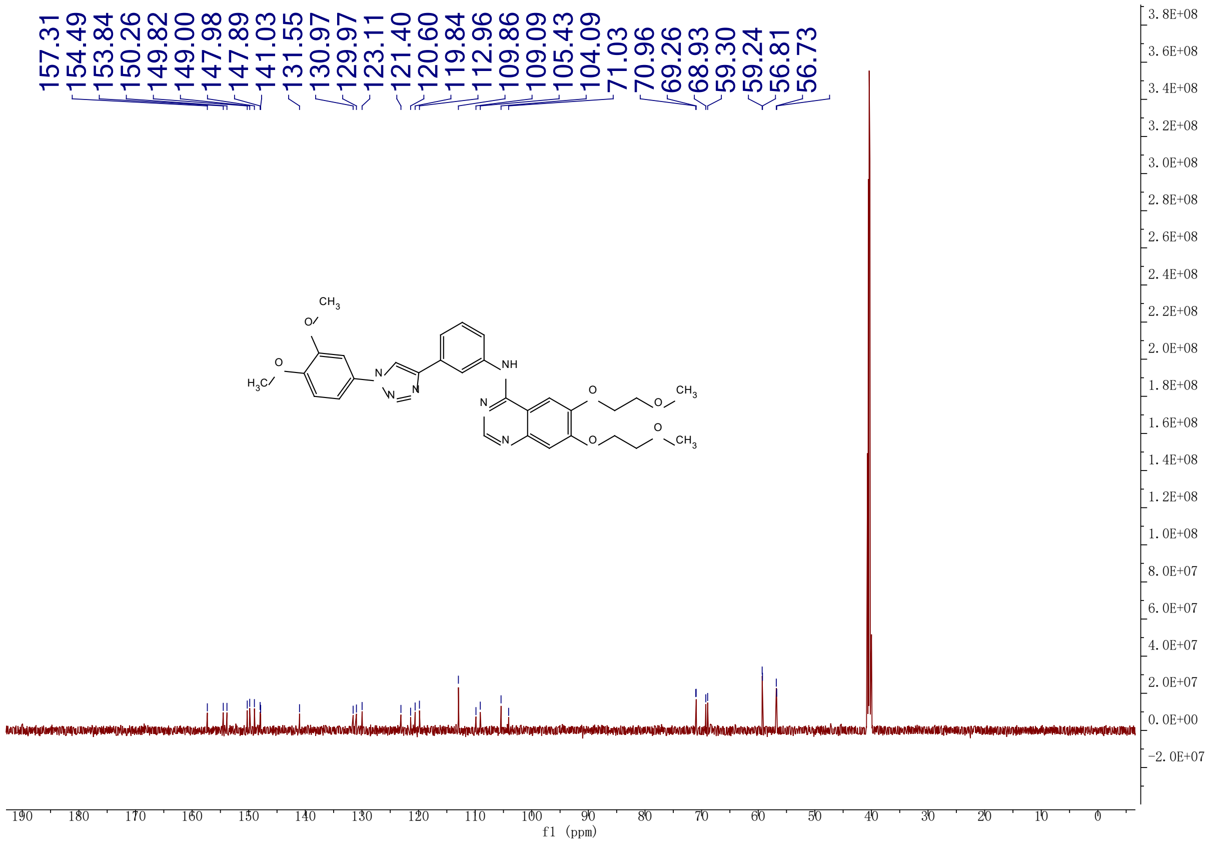

Figure S19-3. HR MS of compound e19

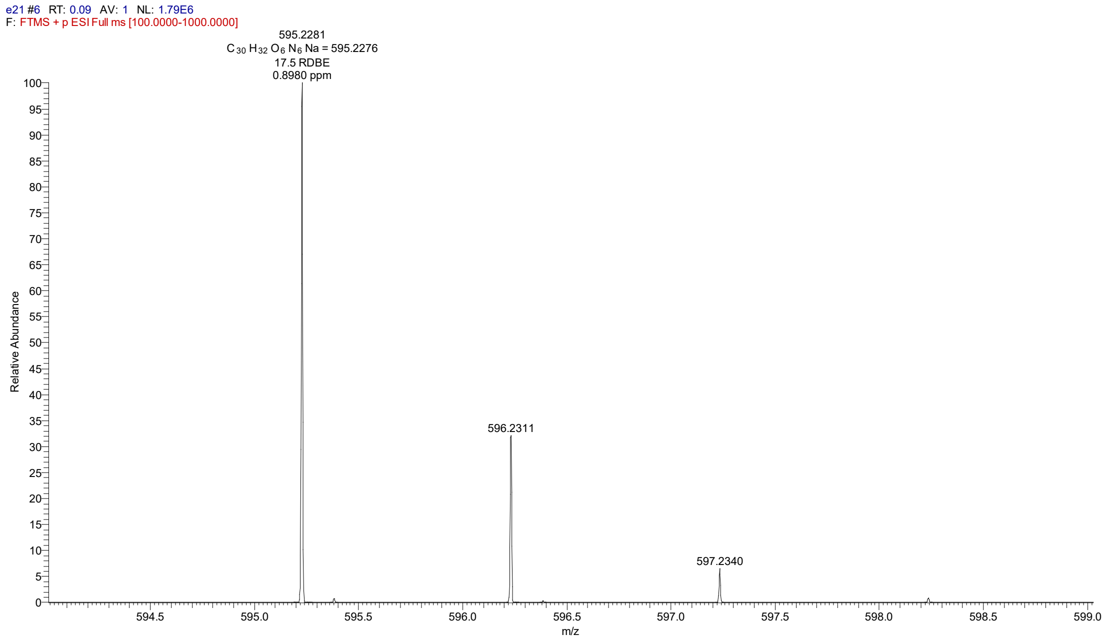

Figure S20-1. ^{1}H NMR spectrum (600 MHz, DMSO-d_6) of compound e20

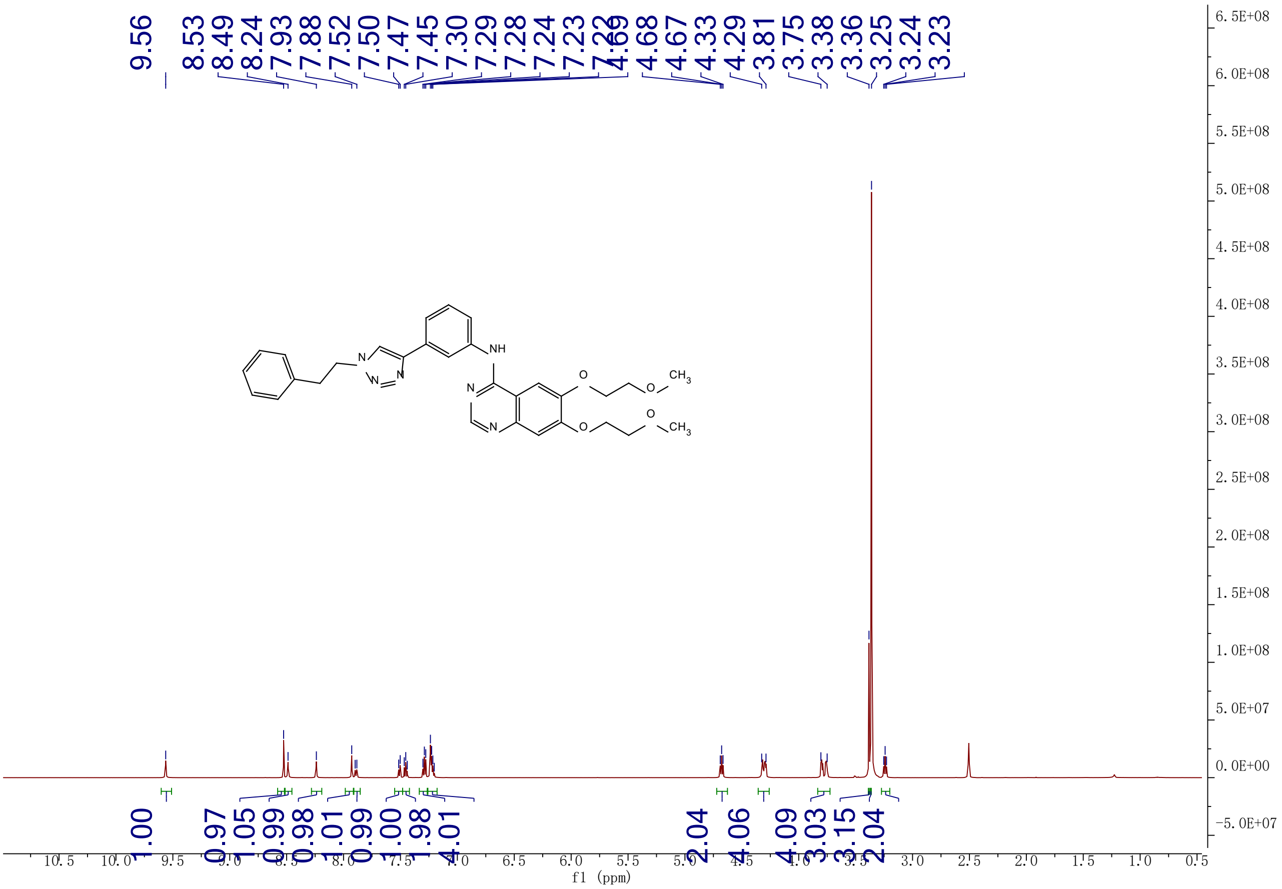

Figure S20-2. ^{13}C NMR spectrum (150 MHz, DMSO-d_6) of compound e20

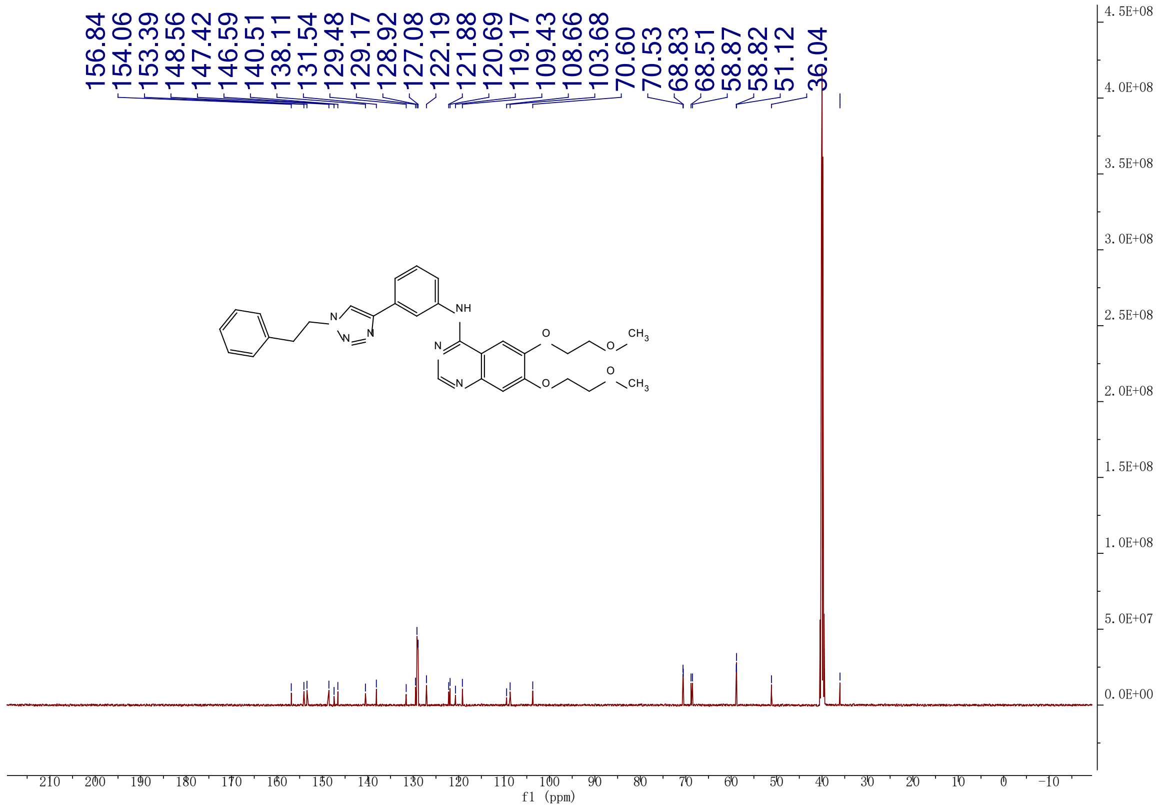

Figure S20-3. HR MS of compound e20

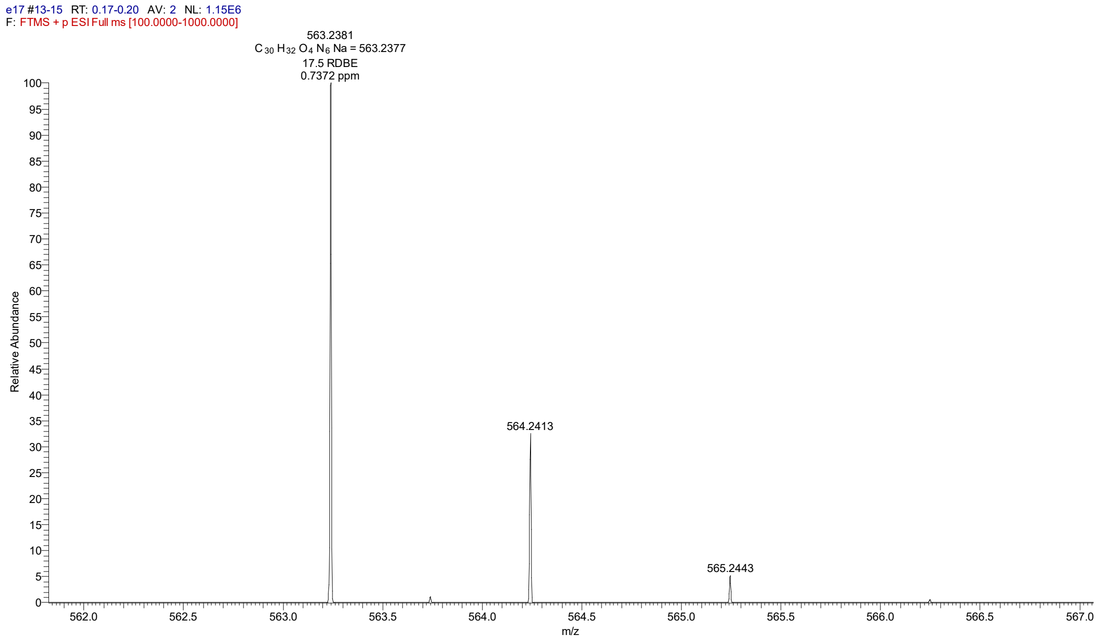

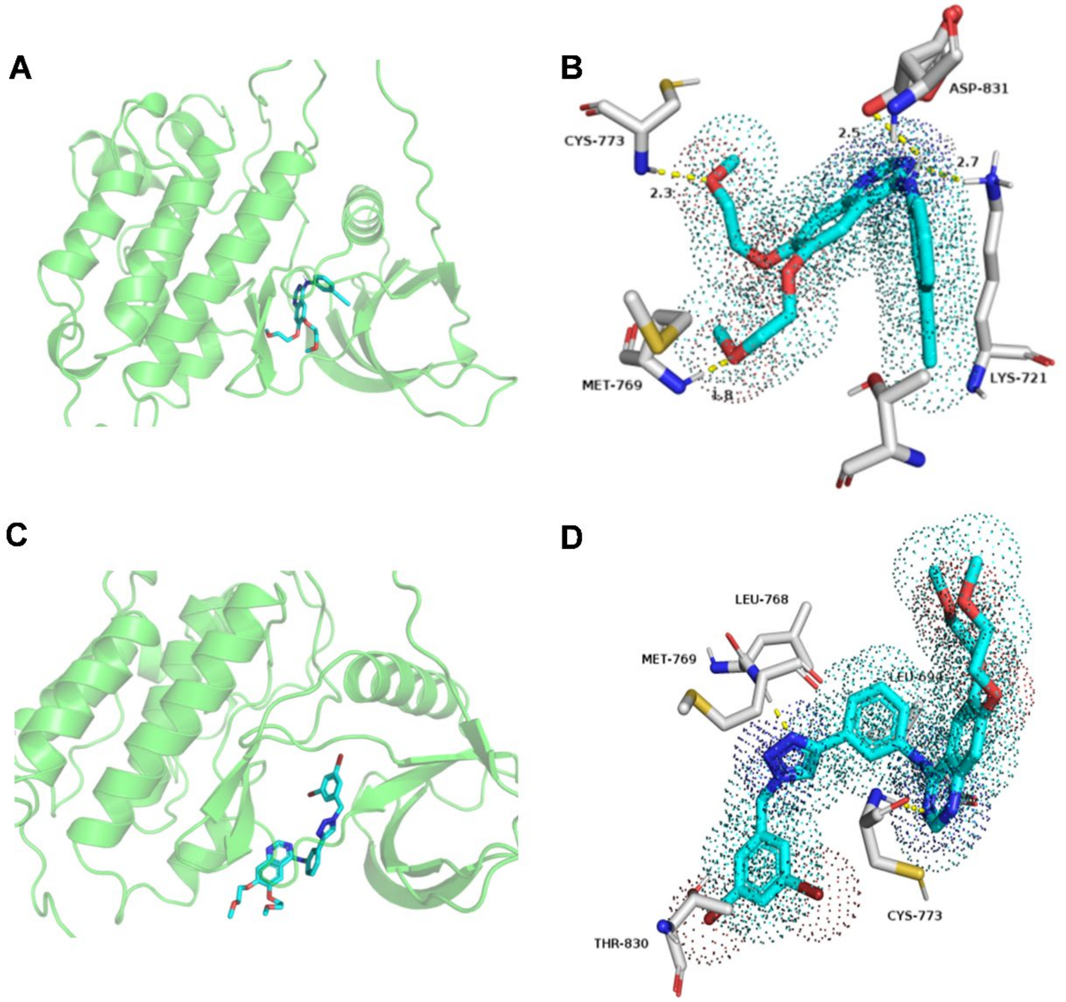

Fig. S21. The binding modes of Erlotinib and compound e4 in complex with EGFR (PDB: 1M17). (A) and (B) The binding mode of Erlotinib in the ATP binding site of EGFR. (C) and (D) The binding mode of compound e4 in the ATP binding site of EGFR.

We conducted molecular docking studies to explore the potential binding modes of compound e4 in the active site of EGFR. The docking studies states that the binding energy of erlotinib is − 7.4 kcal/mol, and Elotinib formed hydrogen bonds with the target amino acid residues including LYS721, MET769, CYS773 and ASP831, and form hydrophobic interactions with THR766. Results show that e4 binds to EGFR in the ATP binding site mainly composed of hydrophobic residues with docking score of -9.6 kcal/mol. We found that the 3,5-dibromobenzy group introduced to the N3 position of 1,2,3-triazole could occupy the hydrophobic pocket containing LEU 694 and THR 830, and the backbone amino group of MET 769 formed a hydrogen bond with one amino of the 1,2,3-triazole group. The amino group of CYS 773 formed hydrogen

bonds with the quinazoline.

The docking results show that e4 has a desirable binding affinity with EGFR but a different binding mode from that of Erlotinib. However, our results show that the EGFR inhibitory activities of Erlotinib is better, but e4 demonstrates preferable anticancer activities in vitro. Thus, more mechanisms about the anticancer activities of these new compounds should be further studied.

Methods

In silico docking was carried out using AutoDock 4.2 [1]. Crystal structure of human epidermal growth factor receptor (EGFR) (PDB ID: 1M17) were downloaded from the protein data bank (https://www.rcsb.org/). Pymol (The PyMOL Molecular Graphics System) programs was used to remove all waters, ligands and co-factors. AutoDock Tools[2] was used to add hydrogens, calculate Gasteiger charges, and generate PDBQT files of compounds and receptor. The grid of 54, 54, and 54 points in x, y, and z directions of EGFR were built with a grid spacing of 0.375 Å and a distance-dependent function of the dielectric constant were used for the calculation of the energetic map. The default settings were used for all other parameters. Lamarckian genetic algorithm method [3] was employed for docking simulations. The standard docking procedure was used for a rigid protein and a flexible ligand whose torsion angles were identified (for 200 independent runs per ligand).

Reference

1. Rizvi, S. M.; Shakil, S.; Haneef, M., A simple click by click protocol to perform docking: AutoDock 4.2 made easy for non-bioinformaticians. **2013,** (1611-2156 (Print)).
2. Morris, G. M.; Huey R Fau - Lindstrom, W.; Lindstrom W Fau - Sanner, M. F.; Sanner Mf Fau - Belew, R. K.; Belew Rk Fau - Goodsell, D. S.; Goodsell Ds Fau - Olson, A. J.; Olson, A. J., AutoDock4 and AutoDockTools4: Automated docking with selective receptor flexibility. **2009,** (1096-987X (Electronic)).
3. Morris, G. M.; Goodsell, D. S.; Halliday, R. S.; Huey, R.; Hart, W. E.; Belew, R. K.; Olson, A. J., Automated docking using a Lamarckian genetic algorithm and an empirical binding free energy function. *Journal of Computational Chemistry* **1998,** *19* (14), 1639-1662.
