# Supplementary material for: Discovery of a Series of 1,2,3-Triazole-Containing Erlotinib Derivatives With Potent Anti-Tumor Activities Against Non-Small Cell Lung Cancer
Source: Front Chem. 2022 Jan 7;9:789030. doi: 10.3389/fchem.2021.789030 (PMC8776995; doi:10.3389/fchem.2021.789030)
Supplement: Supplementary file 16 [file DataSheet12.zip › PC-9 Cell cycle-3/rpt_20200919 PC-9 24H_002_e12 12uM_007.fcs.pdf]

File analyzed: 20200919 PC-9 24H_002_e12 12uM_007.fcs
Date analyzed: 19-Sep-2020
Model: 1Dn0n_DSD
Analysis type: Manual analysis
Auto Linearity: No

Ploidy Mode: First cycle is diploid

Diploid: 100.00 %
Dip G1: 57.79 % at 59.89
Dip G2: 20.78 % at 116.79
Dip S: 21.43 % G2/G1: 1.95
%CV: 1.99

Total S-Phase: 21.43 %
Total B.A.D.: 0.00 % no aggs

Debris: 0.06 %
Aggregates: %
Modeled events: 9470
All cycle events: 9464
Cycle events per channel: 163
RCS: 2.307

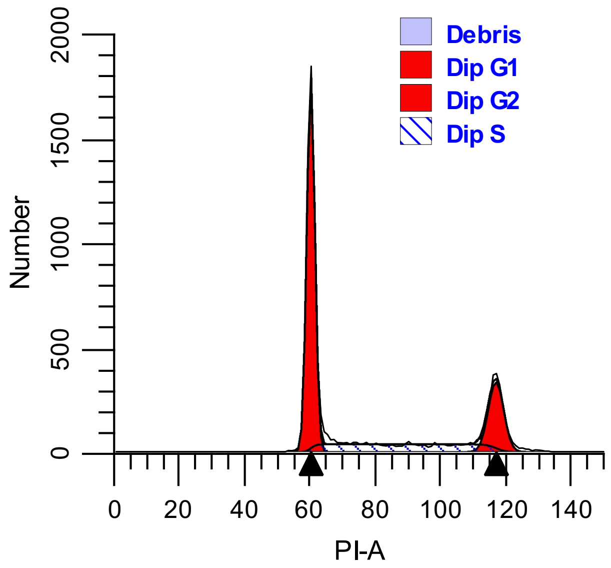

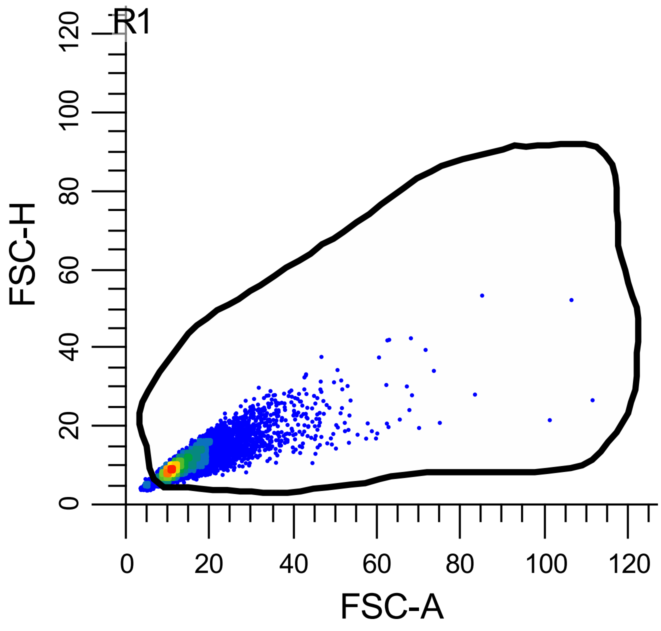

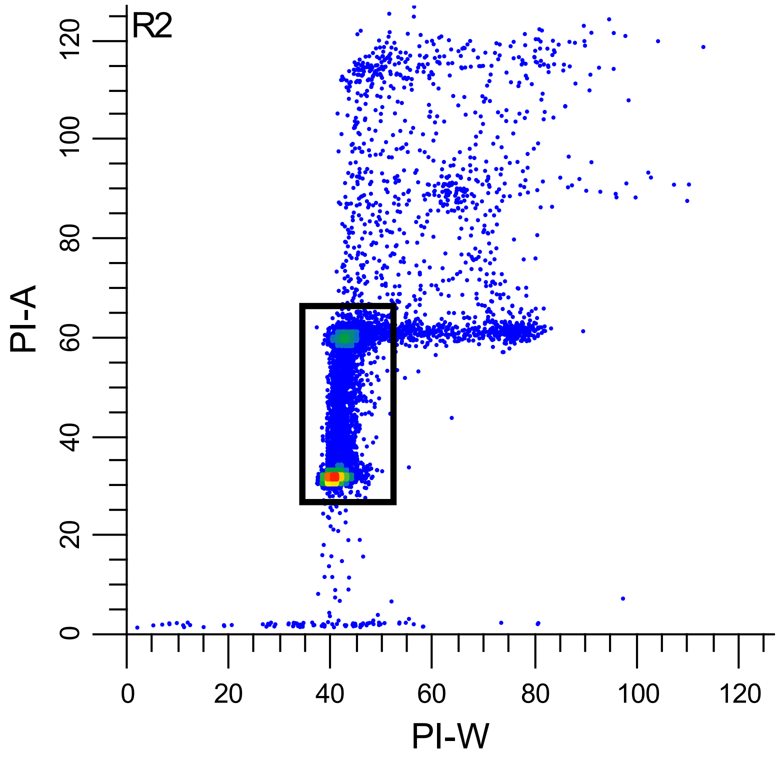

ModFit LT V5.0.9(Win)
