# Supplementary material for: Discovery of a Series of 1,2,3-Triazole-Containing Erlotinib Derivatives With Potent Anti-Tumor Activities Against Non-Small Cell Lung Cancer
Source: Front Chem. 2022 Jan 7;9:789030. doi: 10.3389/fchem.2021.789030 (PMC8776995; doi:10.3389/fchem.2021.789030)
Supplement: Supplementary file 22 [file DataSheet7.zip › H460 Cell cycle-1/rpt_20191202 H460 12h_460 E4 8UM_003.fcs.pdf]

File analyzed: 20191202 H460 12h_460 E4 8UM_003.fcs
Date analyzed: 14-Jul-2020
Model: 1Dn0n_DSD
Analysis type: Manual analysis
Auto Linearity: No

Ploidy Mode: First cycle is diploid

Diploid: 100.00 %
Dip G1: 59.66 % at 53.99
Dip G2: 11.77 % at 104.74
Dip S: 28.56 %   G2/G1: 1.94
%CV: 2.06

Total S-Phase: 28.56 %
Total B.A.D.: 0.00 %   no aggs

Debris: 0.07 %
Aggregates: %
Modeled events: 9553
All cycle events: 9546
Cycle events per channel: 184
RCS: 2.302

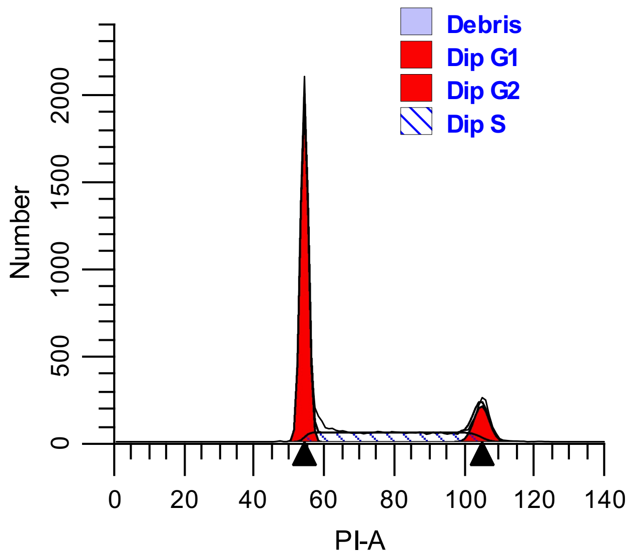

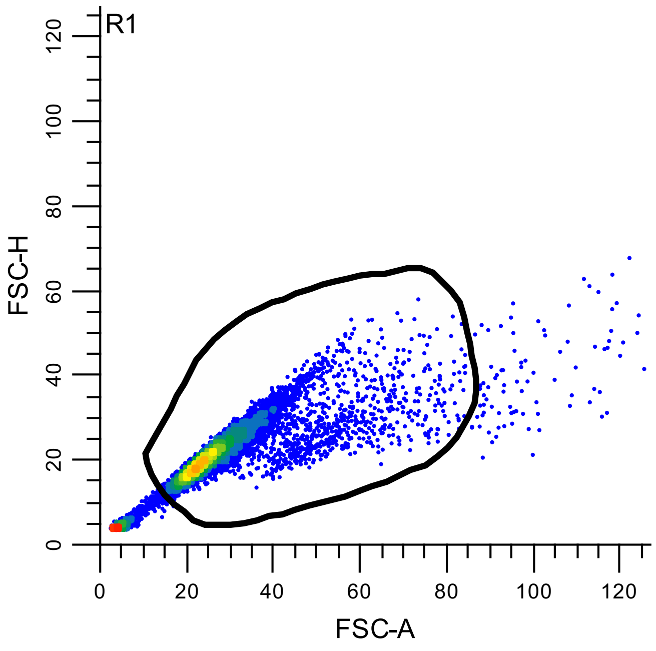

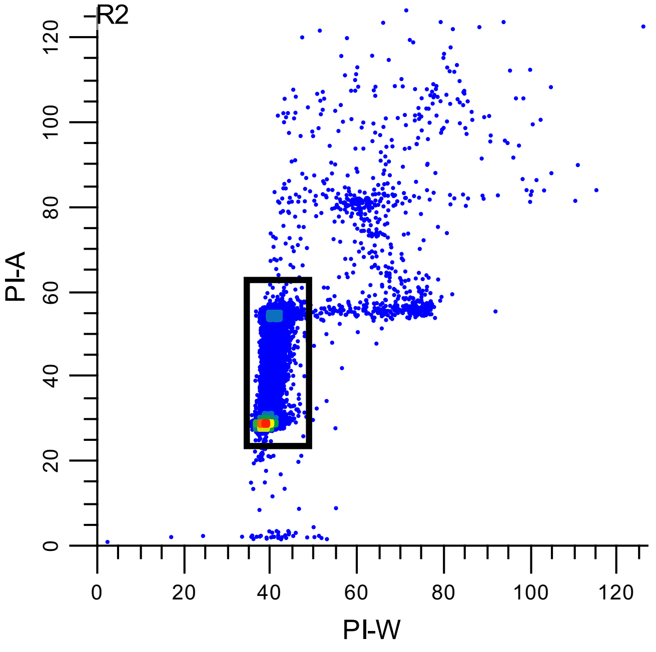

ModFit LT V5.0.9(Win)
